# Menin regulates YBX1 nucleus translocation to boost the HKDC1 transcription and affects pancreatic cancer glycolysis

**DOI:** 10.1016/j.isci.2025.113245

**Published:** 2025-08-07

**Authors:** Chenming Ni, Jiacheng Yang, Yebin Lu, Hongyun Ma, Hao Hu, Xiaohan Shi, Tianlin He, Yijie Zhang, Gang Jin, Peng Cheng

**Affiliations:** 1 Department of Hepatobiliary Pancreatic Surgery, Changhai Hospital Affiliated to Navy Medical University, Shanghai, China; 2 Central South University, Xiangya Hospital, Changsha, China

**Keywords:** Therapeutics, Mechanism of action, Human metabolism, Molecular interaction, Cancer

## Abstract

Pancreatic ductal adenocarcinoma (PDAC) has a bleak prognosis, often driven by aberrant metabolic reprogramming, particularly glycolysis. This study investigated Menin’s role in PDAC metabolism. We found that Menin overexpression significantly suppressed glycolytic markers and activity in PDAC cell lines, a suppression that was reversed by HKDC1 knockdown. Mechanistically, Menin interacts with YBX1, facilitating its nuclear translocation to enhance HKDC1 transcription. *In vivo*, Menin overexpression inhibited tumor growth and glycolysis in xenograft models. These findings indicate that Menin is a critical regulator of PDAC metabolism through the Menin-YBX1-HKDC1 axis, suggesting its potential as a therapeutic target for pancreatic cancer.

## Highlights

• Menin suppresses pancreatic cancer glycolysis *in vitro* and *in vivo*
• Menin interacts with YBX1 to regulate HKDC1 transcription
• Menin promotes YBX1 nucleus translocation to activate HKDC1
• This Menin-YBX1-HKDC1 axis is a potential PDAC therapeutic target

## Graphical abstract

**Figure:**
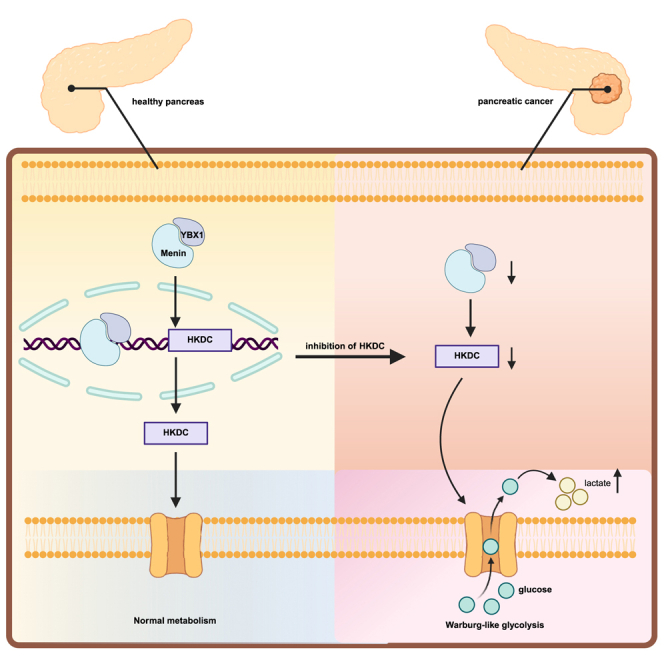

## Introduction

Pancreatic cancer remains one of the leading causes of cancer-related deaths worldwide, often presenting a bleak prognosis for those diagnosed, as evidenced by a survival rate of less than 10% over five years.^1^ Approximately 95% of these cases are tumors originating from the exocrine part of the pancreas, with pancreatic ductal adenocarcinoma (PDAC) being the predominant type. Despite advances in medical science, current treatment strategies—including chemotherapy, radiotherapy, and surgical interventions—provide limited benefits for PDAC patients.^2^^,^^3^ This situation highlights the urgent need to understand the intricate molecular mechanisms underlying PDAC, which could lead to the development of more effective treatments.

One of the defining characteristics of PDAC tumors is their metabolic reconfiguration, tailored to support incessant proliferation.^4^^,^^5^ Profound metabolic studies have spotlighted the Warburg effect, or aerobic glycolysis, as a central metabolic shift within pancreatic cancer tissues that potentiates their malignant trajectory.^6^ This inclination, while seemingly inefficient, rapidly churns out ATP, meeting the insatiable energy demands of these proliferating cells.^7^^,^^8^ Consequently, targeting the glycolysis pathway in PDAC is emerging as a promising therapeutic strategy.^9^^,^^10^ While many factors influencing PDAC glycolysis have been identified,^11^^,^^12^ the practical implementation of this approach requires further supportive data and more efficient targets.

In our previous research, we identified a notable downregulation of Menin during pancreatic carcinogenesis, and observed that its overexpression significantly hindered the growth of pancreatic cancer cells.^4^^,^^5^ Menin was also reported to play as a tumor suppressor in epigenetic modulation and acts as a bulwark against tumor progression in a range of malignancies, including leukemia, ovarian, breast, and endometrial cancers.^13^^,^^14^^,^^15^^,^^16^ Serving as a scaffold protein, Menin facilitates the interaction of diverse cellular factors and transitions dynamically between the nucleus and cytoplasm.^17^^,^^18^ And within the nuclear domain, it is intricately involved in transcriptional modulation, DNA replication, and DNA repair processes.^4^^,^^19^^,^^20^ However, the specific role Menin plays in PDAC metabolism remains to be comprehensively understood.

Y-box binding protein 1 (YBX1) represents a multifunctional RNA/DNA-binding protein that orchestrates diverse cellular processes, encompassing transcriptional regulation, translational control, mRNA splicing, and DNA damage response mechanisms.^21^ The functional outcomes of YBX1 are critically determined by its subcellular localization, with nucleocytoplasmic shuttling serving as a pivotal regulatory mechanism that dictates distinct gene expression programs.^22^ Under specific cellular conditions or stress stimuli, YBX1 undergoes nuclear translocation where it functions as a transcriptional regulator, capable of both activating and repressing target genes depending on the chromatin context and associated cofactors.^23^^,^^24^^,^^25^ This functional plasticity has positioned YBX1 as an important regulatory node in various pathological processes, though its precise role often depends on the cellular environment and the specific protein complexes within which it operates.^23^

Hexokinase domain-containing protein 1 (HKDC1) belongs to the hexokinase enzyme family, which catalyzes the initial phosphorylation of glucose to glucose-6-phosphate in cellular glucose metabolism. While structurally related to canonical hexokinases such as HK1 and HK2, HKDC1 exhibits distinct regulatory properties and tissue distribution patterns that suggest specialized metabolic functions.^26^ Recent investigations across various cancer types have identified HKDC1 as a significant player in metabolic reprogramming, with studies in gastric, lung, and colorectal malignancies demonstrating its involvement in cellular glucose utilization.^27^^,^^28^ However, the mechanistic basis of HKDC1 function appears to be highly context dependent, with emerging evidence suggesting that its metabolic impact may vary considerably based on the specific regulatory networks and protein interaction environments present in different cellular states.^29^ This regulatory complexity indicates that HKDC1’s role in cancer metabolism extends beyond simple enzymatic activity and likely involves sophisticated regulatory mechanisms that remain incompletely understood.^30^

This study aims to confirm the function of Menin on PDAC glycolysis and investigate the downstream molecular mechanism. Our findings indicate that Menin associates with YBX1, subsequently enhancing the transcription of HKDC1, a known regulator of cancer glycolysis.^31^^,^^32^^,^^33^ Collectively, the data suggest that Menin modulates both glucose and lipid metabolism to maintain the energy equilibrium within PDAC cells, presenting a potential therapeutic target for PDAC.

## Results

### *Menin* suppressed the glycolysis of PDAC *in vitro*

**Figure 1:**
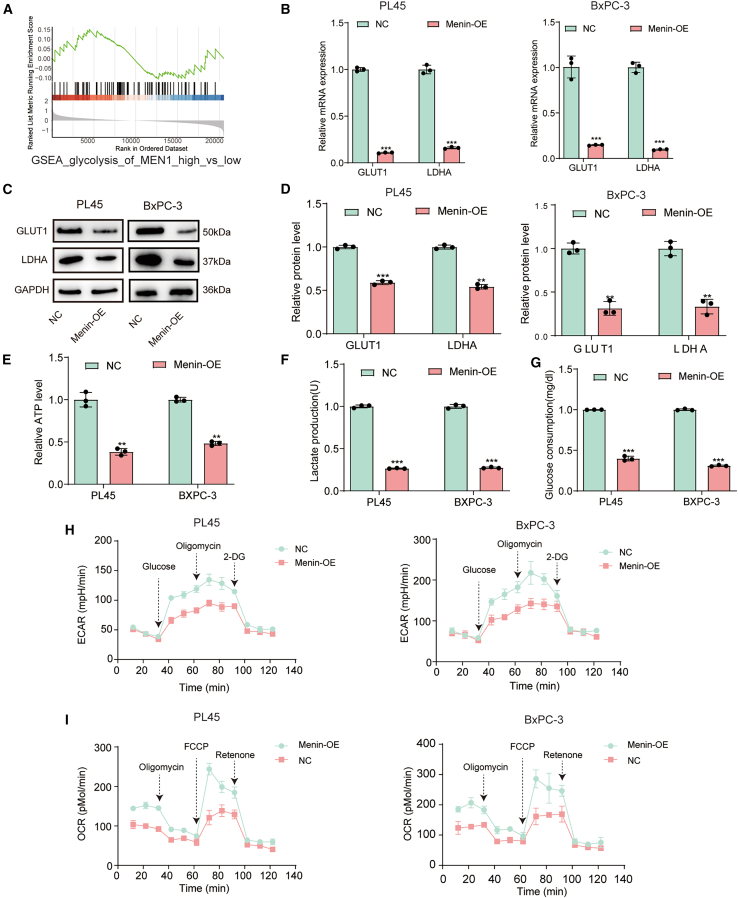
Menin suppressed the glycolysis of PDAC *in vitro* (A) Gene set enrichment analysis (GSEA) based on the TCGA database to explore the relationship between Menin expression and glycolysis pathway. (B–D) RT-qPCR and western blot were utilized for detecting the glycolysis-associated protein GLUT1 and LDHA in Menin-modulated PL45 and Bx-PC3 cells. (E–G) Relative ATP, extracellular lactatequantification, and total glucose consumption of Menin-overexpression and control vector-transfected PL45 and Bx-PC3 cells. (H) Glucose stress test via measuring the extracellular acidification rate (ECAR) in Menin-overexpression and control vector-transfected PL45 and Bx-PC3 cells. 2-DG: 2-deoxyglucose. (I) Real-time analysis of oxygen consumption rate (OCR) in Menin-overexpression and control vector-transfected PL45 and Bx-PC3 cells. FCCP: carbonyl cyanide p-(trifluoromethoxy) phenylhydrazone. ∗∗∗*p* < 0.001 when comparing Menin-OE plasmid transfected cells to control vector-transfected cells. Error bars represent mean ± SD. Each experiment was conducted in biological triplicates for each condition.

Building upon our previous characterization of *Menin*’s relationship with *Dnmt1* in Hedgehog-mediated pancreatic cancer growth regulation, we investigated Menin’s role in PDAC metabolism. Comparative analysis of *Menin* expression levels in TCGA datasets revealed significant enrichment of the glycolysis pathway in low *Menin*-expressing samples (Figure 1A). Examination of clinicopathological correlations demonstrated that *Menin* expression did not significantly associate with patient age (*p* = 0.68), gender (*p* > 0.99), histological grade (*p* = 0.51), or tumor stage (*p* = 0.45), suggesting that while expression differences between groups were significant (*p* < 0.001), baseline *Menin* mRNA levels do not directly correlate with these specific progression indicators (Table 1). *In vitro* validation confirmed robust Menin upregulation at both mRNA and protein levels in *Menin*-OE groups compared to controls (Figures S1A and S1B), with subsequent western blot analysis revealing marked downregulation of glycolytic markers GLUT1 and LDHA following Menin overexpression (Figures 1B–1D).

**Table 1:** Association between MEN1 expression and clinicopathological features in PDAC patients

Characteristic	N	Overall *N* = 146^a^	MEN1_High *N* = 73^a^	MEN1_Low *N* = 73^a^	*p*-value^b^
Age	146	64.62 (10.83)	65.10 (10.34)	64.14 (11.34)	0.68
Gender	146				>0.99
FEMALE		68/146 (47%)	34/73 (47%)	34/73 (47%)	
MALE		78/146 (53%)	39/73 (53%)	39/73 (53%)	
Grade	146				0.51
G1		21/146 (14%)	13/73 (18%)	8/73 (11%)	
G2		83/146 (57%)	39/73 (53%)	44/73 (60%)	
G3		41/146 (28%)	20/73 (27%)	21/73 (29%)	
G4		1/146 (0.7%)	1/73 (1.4%)	0/73 (0%)	
Stage	145				0.45
IA		3/145 (2.1%)	2/72 (2.8%)	1/73 (1.4%)	
IB		9/145 (6.2%)	3/72 (4.2%)	6/73 (8.2%)	
IIA		23/145 (16%)	11/72 (15%)	12/73 (16%)	
IIB		104/145 (72%)	55/72 (76%)	49/73 (67%)	
III		3/145 (2.1%)	0/72 (0%)	3/73 (4.1%)	
IV		3/145 (2.1%)	1/72 (1.4%)	2/73 (2.7%)	
(Missing)		1	1	0	
MEN1	146	9.62 (0.33)	9.88 (0.19)	9.36 (0.21)	<0.001

a Mean (SD); n/N (%).

b Wilcoxon rank-sum test; Fisher’s exact test.

Functional metabolic assessments demonstrated that *Menin* overexpression significantly reduced cellular ATP levels, lactate production, and glucose consumption in both cell lines (Figures 1E–1G). Extracellular flux analysis further revealed that *Menin* overexpression substantially decreased glycolytic capacity and rate in both PL45 and BxPC-3 cells (Figure 1H), while concurrently altering basal and maximal oxygen consumption rates compared to vector controls (Figure 1I). Collectively, these findings indicate Menin is a regulator of glycolytic metabolism in pancreatic cancer cells.

### *Menin* regulates PDAC proliferation and migration via glycolysis

**Figure 2:**
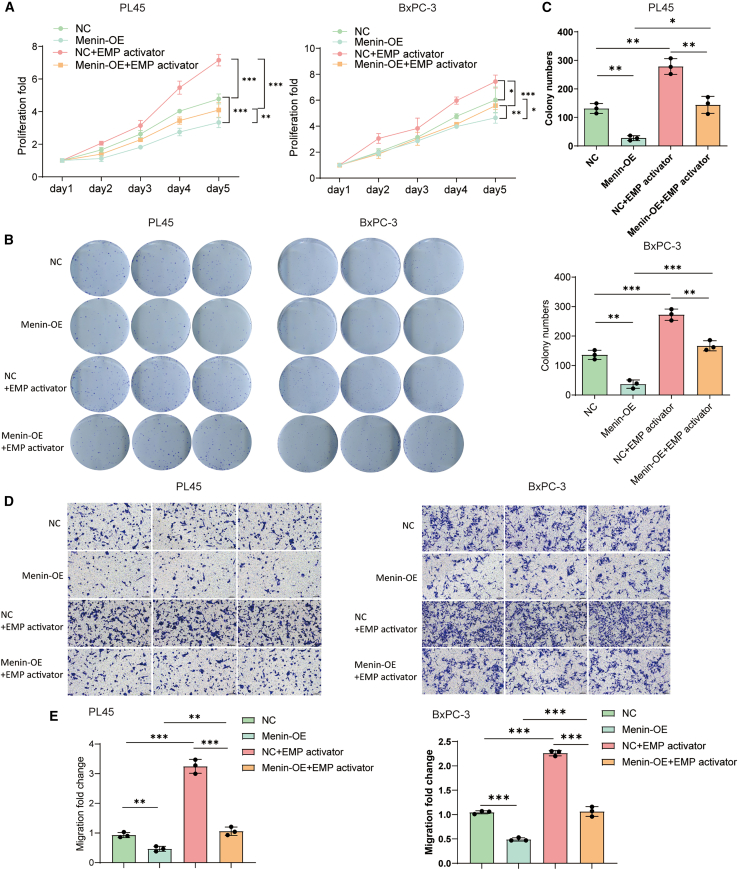
Menin regulates proliferation, colony formation and migration via glycolysis (A) CCK-8 assays show the 5-day proliferation curves of PL45 and BxPC-3 cells stably over-expressing Menin or a negative-control vector (NC) cultured with or without the glycolysis activator EMP; values are mean ± SD. (B) Representative crystal-violet–stained colonies formed by PL45 and BxPC-3 cells. (C) Quantification of colony numbers from (B); data are mean ± SD. (D) Representative crystal-violet–stained Transwell images of migrated PL45 and BxPC-3 cells obtained under the indicated conditions; scale bars, 100 μm. (E) Quantification of relative cell migration (fold change) from (D); values are mean ± SD. ∗*p* < 0.05, ∗∗*p* < 0.01, and ∗∗∗*p* < 0.001.

To investigate the consequences of aberrant glycolysis caused by *Menin*, proliferation, colony formation, and migration ability of PL45 and BxPC-3 cell lines were estimated after overexpression of *Menin* and/or adding EMP activator, a glycolysis activator. Proliferation rate of cell lines were significantly decreased after *Menin* overexpression but restored by EMP activator (Figure 2A). Consistently, *Menin* overexpression decreased colony number, while after adding EMP activator colony formation was also restored (Figures 2B and 2C). Migration ability regulated by glycolysis and *Menin* was also analyzed. As expected, EMP activator disrupted the function of *Menin* in migration (Figures 2D and 2E). These results indicate that *Menin* regulates progression phenotypes of PDAC via glycolysis.

### Menin regulates glycolysis via affecting HKDC1 gene expression

**Figure 3:**
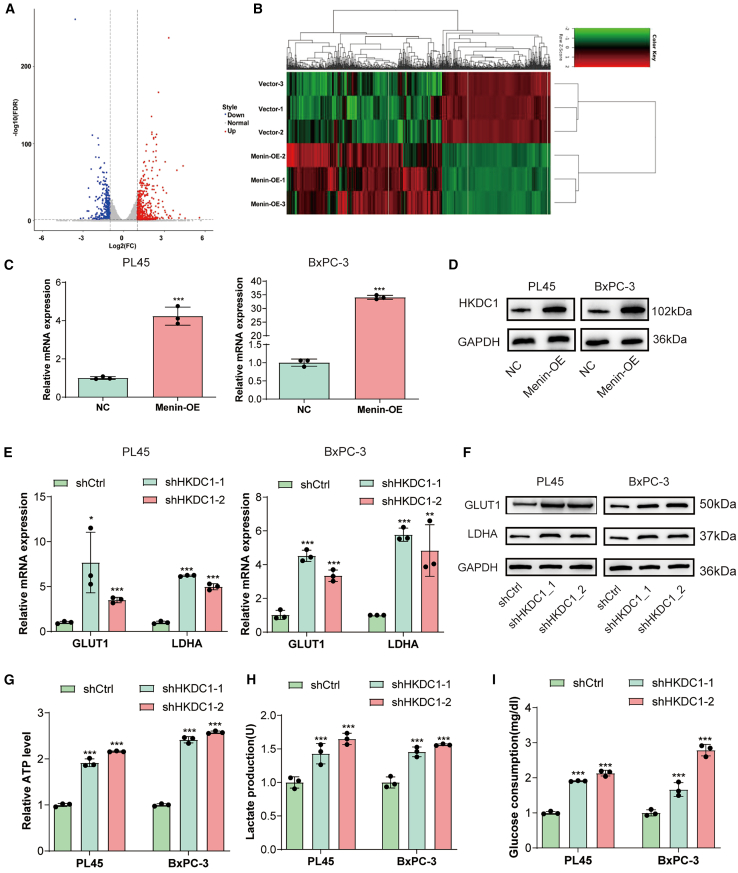
Menin regulates glycolysis via affecting HKDC1 gene expression (A) Volcano plot illustrating upregulated and downregulated genes in Menin-overexpressing (Menin-OE) PL45 cells in comparison to the vector control group. (B) Heatmap representation of differentially expressed genes from the RNA-seq analysis. (C and D) Measurement of HKDC1 expression after Menin-overexpression in PL45 and BxPC-3 cells detected by RT-qPCR and western blot. (E and F) Expression levels (both mRNA and protein) of GLUT1 and LDHA in PL45 and BxPC-3 cells following HKDC1 silencing, as observed through RT-qPCR and western blot. (G–I) ATP, lactate secretion, and glucose consumption levels in HKDC1-silenced versus control PL45 and Bx-PC3 cell. ∗∗∗*p* < 0.001. Error bars represent mean ± SD. Each experiment was conducted in biological triplicates for each condition.

To understand the broader transcriptional ramifications of Menin overexpression, we undertook RNA sequencing (RNA-seq) analysis using *Menin*-overexpressed PL45 cells alongside their control counterparts. This comprehensive analysis pinpointed a total of 413 genes that were significantly influenced by *Menin* overexpression: 252 were upregulated, while 161 faced downregulation (Figures 3A and 3B). The most prominently upregulated genes included *AKR1B10*, *ARSB*, *HKDC1*, *FBP1*, and *TYMP*. Subsequent validation via RT-qPCR affirmed the augmented mRNA levels of *HKDC1*, *FBP1*, and *TYMP* in the *Menin-OE* cohort relative to the vector control group (Figure S2A). Given existing literature highlighting the intimate association of *HKDC1* with glycolytic regulation, we earmarked *HKDC1* as the potential mediator through which Menin orchestrates its influence on glycolysis.

Compared to the vector groups, there was a notable upregulation of HKDC1 at both mRNA and protein levels (*p* < 0.001, Figures 3C and 3D) in Menin-OE group. To delve deeper into HKDC1’s role in PDAC cell glycolysis, its expression was suppressed in PL-45 and Bx-PC3 cells through transfection with HKDC1 shRNA. Validated by RT-qPCR and western blot analyses, a significant decrease in HKDC1 was observed in the shHKDC1 group relative to the control shRNA group (*p* < 0.001, Figures S2B and S2C). Intriguingly, upon HKDC1 suppression, there was a concurrent rise in the expression of GLUT1 and LDHA (Figures 3E and 3F) in both cell lines. Moreover, enhanced ATP levels, lactate production, and glucose consumption were evident following HKDC1 knockdown (Figures 3G–3I). Collectively, Menin regulates HKDC1 to affect glycolysis.

### HKDC1 manipulation restores glycolysis regulated by Menin

**Figure 4:**
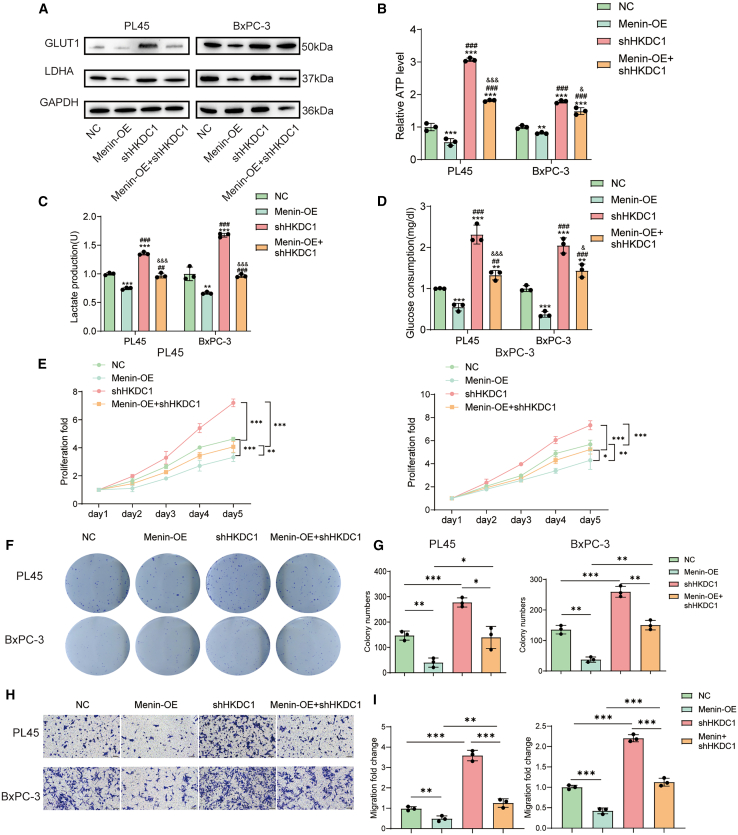
HKDC1 knock-down rescues Menin-suppressed glycolysis and the associated oncogenic phenotypes (A) Western-blot analysis of GLUT1 and LDHA protein levels in PL45 and BxPC-3 cells stably expressing a negative control (NC), Menin over-expression (Menin-OE), HKDC1 short hairpin RNA (shHKDC1), or Menin-OE + shHKDC1; GAPDH is the loading control. (B–D) Quantification of relative cellular ATP levels (B), lactate production (C), and glucose consumption (D) under the indicated conditions. (E) Five-day CCK-8 proliferation curves of PL45 and BxPC-3 cells in the same groups. (F) Representative crystal-violet-stained colonies produced by PL45 and BxPC-3 cells. (G) Quantification of colony numbers from (F). (H) Representative crystal-violet-stained Transwell micrographs of migrated PL45 and BxPC-3 cells; scale bars, 100 μm. (I) Quantification of relative cell migration (fold change) from (H). All quantitative data are mean ± SD. ∗∗*p* < 0.01 and ∗∗∗*p* < 0.001 versus the Menin-OE group.

Furthermore, the simultaneous overexpression of *Menin* and knockdown of *HKDC1* restored the expression levels of *GLUT1* and *LDHA* in both PL45 and Bx-PC3 cells (Figure 4A). In co-regulation group with overexpression of *Menin* and downregulation of *HKDC1*, there was an increase in cellular ATP, lactate levels, and glucose consumption compared to the group with only *Menin* overexpression (Figures 4B–4D). Moreover, knocking down of *HKDC1* also restored the suppressed proliferation, clone formation, and cell migration phenotypes (Figures 4F–4I). In summary, these results suggest that *Menin* regulated glycolysis can be restored by *HKDC1*. In summary, *HKDC1* manipulation restores the phenotypes caused by *Menin*.

### Menin binds transcription factor YBX1 to regulate glycolysis

**Figure 5:**
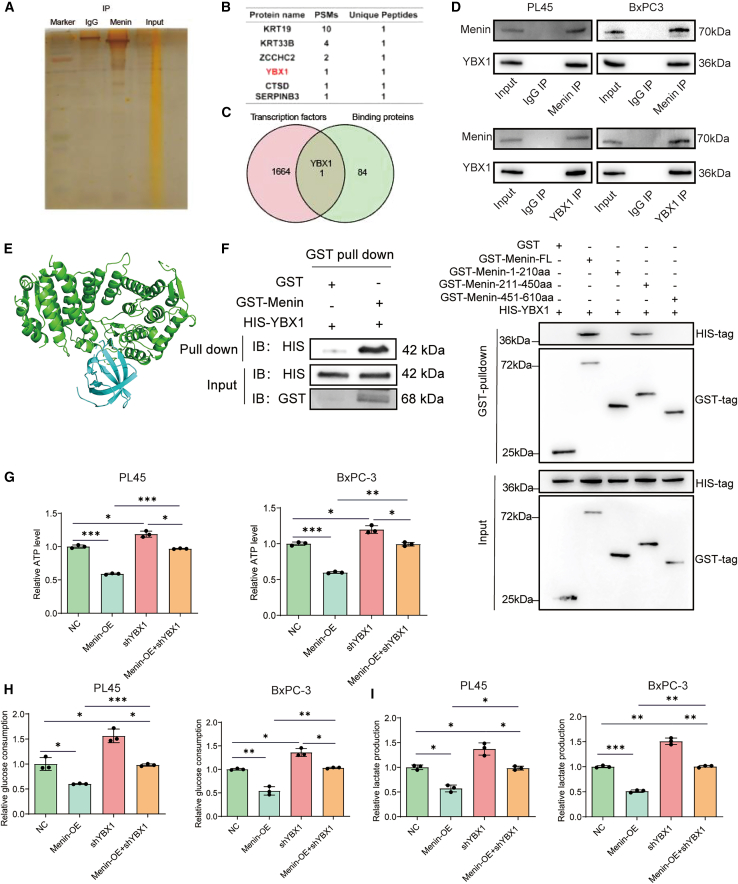
Menin binds transcription factor YBX1 to regulate glycolysis (A) Immunostaining of PL45 cell protein samples following immunoprecipitation with IgG and anti-Menin antibody. (B) Analysis of peptide segments from mass spectrometry to identify proteins interacting with Menin. (C) Venn diagram highlighting the overlap between proteins associated with Menin and potential transcription factors of HKDC1. (D) Co-immunoprecipitation confirmed the direct combination between Menin and YBX1 in PL45 and Bx-PC3 cells. (E) Predicted interaction of Menin and YBX1. F, GST pull-down assays demonstrating direct *in vitro* interaction between Menin and YBX1. Purified GST-Menin (but not GST alone) pulled down HIS-YBX1. (F) Further mapping using GST-tagged Menin truncation mutants revealed that the interaction with HA-YBX1 is mediated primarily through the central and C-terminal regions of Menin (amino acids 211–610), as fragments containing these regions, but not the N-terminal (1-210 aa) fragment, showed binding. Blots were probed with indicated antibodies (anti-HIS, anti-GST, anti-HA). (G–I) Glycolysis indicators (ATP, lactate, and glucose consumption) influenced by Menin was restored by YBX1 knocking down in both PL45 and BxPC-3 cell lines,∗*p* < 0.05, ∗∗*p* < 0.01, and ∗∗∗*p* < 0.001 when comparing Menin-OE plasmid transfected cells to control vector-transfected cells. Error bars represent mean ± SD.

Menin is widely recognized as a scaffold protein, leading us to hypothesize that other proteins might collaborate with Menin in regulating HKDC1. To identify potential interacting partners that work alongside Menin, we conducted immunoprecipitation-mass spectrometry analysis in PL45 cells ((Figure 5A). As a result, a total of 85 proteins were identified to interact with Menin (highlighted in Figure 5B). Online database-driven predictions of potential transcription factors for HKDC1, cross-referenced through a Venn diagram, identified YBX1 as a potential interacting partner of Menin and a probable transcription factor for HKDC1. (Figure 5C). Accordingly, using co-immunoprecipitation, where either YBX1 or Menin was pulled down, we verified the interaction between Menin and YBX1 in both PL45 and Bx-PC3 cells (Figure 5D). Also, YBX1 and HKDC1 were shown to bind affinity according to computational algorithm (Figure 5E). Using GST pull down, the prediction was verified (Figure 5F). Consistently, knocking down of *YBX1* (Figure S3A) in also restored the glycolysis phenotypes (ATP level, glucose consumption, and lactate production) in both PL45 and Bx-PC3 cells (Figures 5G–5I). These results indicate that Menin binds to YBX1 in PDAC.

### Menin regulates YBX1 nucleus localization to initiate HKDC1 transcription

Clarity of mechanism: HKDC1 regulation by Menin-YBX1. We therefore delineated how the MEN2 modifier Menin impacts on YBX1 function by investigating the effect of Menin on YBX1ne function.

**Figure 6:**
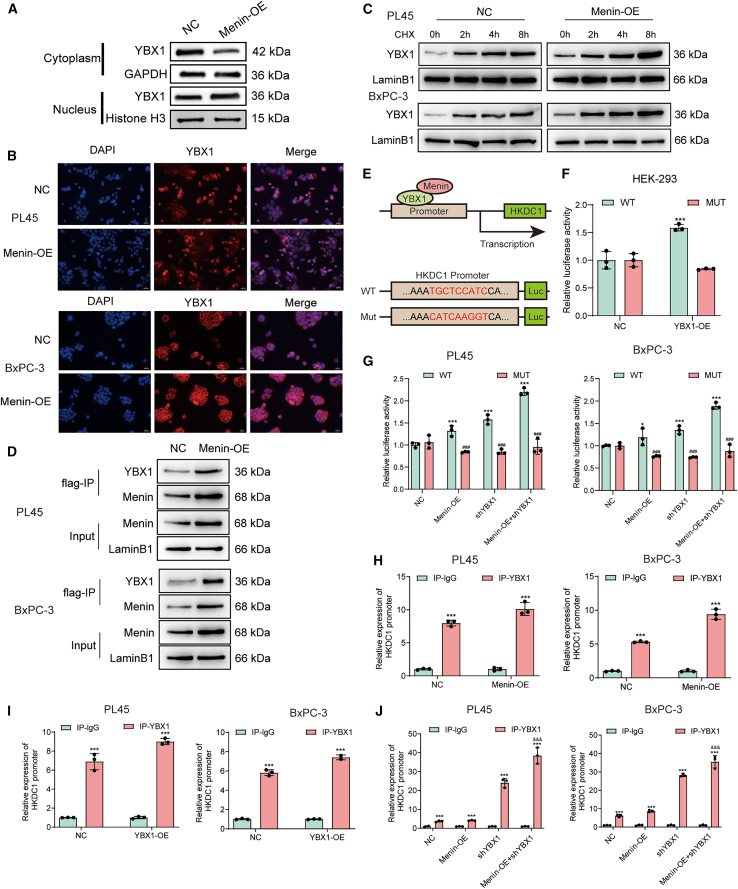
Menin induces nuclear localization of YBX1 and activates HKDC1 transcription (A) Immunoblotting of cytoplasmic and nuclear fractions shows that Menin over-expression (Menin-OE) markedly increases nuclear YBX1 in PL45 and BxPC-3 cells compared with the negative control (NC); GAPDH and Histone H3 serve as cytoplasmic and nuclear loading controls, respectively. (B) Immunofluorescence imaging confirms the enhanced nuclear accumulation of YBX1 (red) following Menin-OE, with nuclei counter-stained by DAPI (blue); scale bars, 200 μm. (C) Cycloheximide (CHX) chase assays (0–8 h) reveal that Menin-OE does not affect YBX1 protein stability in either cell line (LaminB1 loading control). (D) FLAG immunoprecipitation of nuclear extracts demonstrates a physical association between Flag-tagged Menin and endogenous YBX1 in PL45 and BxPC-3 cells, with LaminB1 as the input control. (E) Schematic of HKDC1-promoter luciferase reporters containing a wild-type (WT) or mutated (Mut) YBX1-binding motif. (F) Dual-luciferase assays in HEK-293 cells show that YBX1 activates the WT reporter but not the Mut reporter. (G) In PL45 and BxPC-3 cells, Menin-OE and YBX1 co-expression synergistically enhance WT-reporter activity, whereas mutation of the YBX1 site abolishes this effect. (H) ChIP-qPCR confirms increased recruitment of YBX1 to the HKDC1 promoter upon Menin-OE. (I) YBX1 over-expression alone serves as a positive control. (J) Combined Menin-OE and YBX1-OE further augment YBX1 binding relative to vector or IgG controls. Quantitative data are presented as mean ± SD (*n* = 3); ∗∗∗*p* < 0.001 versus the indicated control.

Analysis of western blotting showed that both PL45 and BxPC-3 cell lines (in addition), Menin overexpression substantially enhanced nuclear YBX1 accumulation and cytoplasmic depletion as detected by immunofluorescence showing that nuclear YBX1 colocalization patterns were increased in Menin-overexpressing cells, in in contrast Menin-expressing cell colonies (Figure 6A) recapitulating these results using cycloheximide chase analysis Menin overexpression did not impact on the stability of YBX1 (Figure 6C, no Menin affects stability over 8 h), indicating that YBX1 subclonal fate rather than protein turnover in part defined by Menin. An additional step using co-immunoprecipitation from nuclear extracts showed direct interaction in both cell lines of FLAG-tagged Menin and endogenous YBX1 (Figure 6D). First functionally (Figure 6E) luciferase reporter assays in HEK-293 provided the basis that YBX1 overexpression stimulated the WT, but not Mut promoter (Figure 6F) of wild-type HKDC1 (Figure 6E), validating in this case a direct relationship to the binding site of YBX1. Overexpression of Menin as well as YBX1 in PDAC cell lines improved WT HKDC1 promoter activity individually and their co-expression synergized activation; there were no significant changes in HKDC1 promoter activity with the Mut construct under any condition (Figure 6G), as previously confirmed by YBX1 knockdown (Figures S3B–S3D). ChIP-qPCR showed that YBX1 overexpression increased YBX1 peak occupancy at the HKDC1 promoter (Figure 6I) and uniquely overexpression of Menin alone (Figure 6H) while both factors were co-overexpressed further prominently increased recruitment into these regions (Figure 6J). In agreement with these results, the ChIP-qPCR experiments using anti-Menin antibody showed that Menin is enriched at the HKDC1 promoter not only itself overedited (Figure S4A). YBX1 alone overexpression also increased the endogenous recruitment of Menin to the HKDC1 promoter (Figure S4B), and again overexpression of both factors resulted in this mutual best recruitment (highest Menin occupancy) when both were co-overexpressed (Figure S4C). In summary, our findings show that Menin promotes HKDC1 transcription by facilitating YBX1 nuclear import and its recruitment to the HKDC1 promoter.

### Menin suppressed the glycolysis *in vivo*

**Figure 7:**
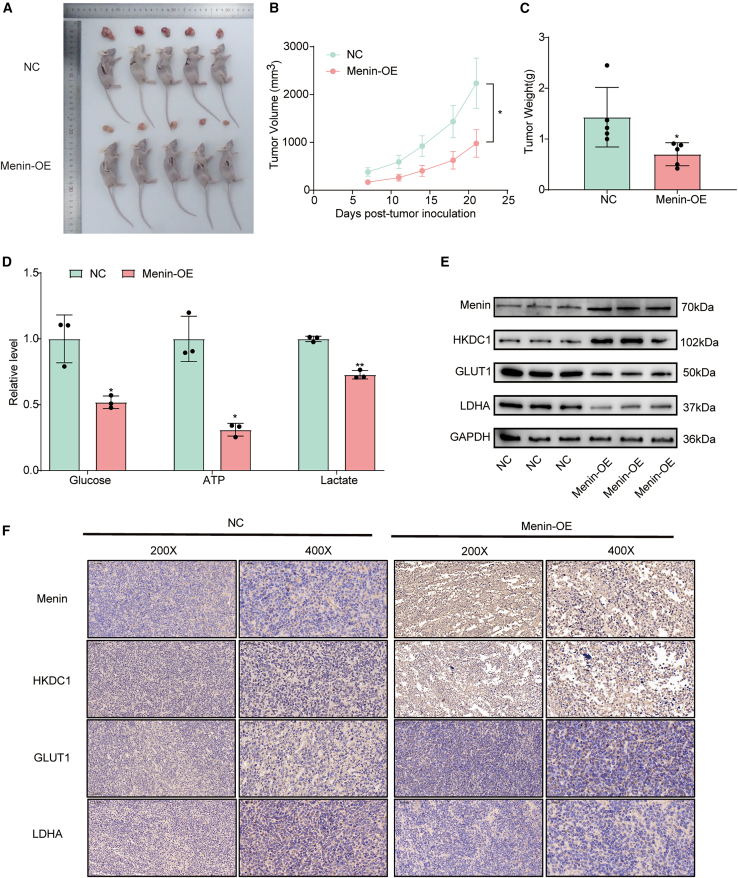
Menin over-expression suppresses tumor growth and attenuates glycolytic activity in a PL45 xenograft model (A) Representative photographs of nude mice bearing PL45 xenografts and the corresponding tumors immediately after excision (scale bars, 1 cm). (B) Tumor-volume growth curves (mean ± SD, *n* = 5) recorded every three days from implantation to day 24; ∗∗∗*p* < 0.001 versus NC at the endpoint. (C) Final tumor weights at sacrifice (mean ± SD, *n* = 5). (D) Intratumoral concentrations of glucose, ATP, and lactate measured in freshly isolated tumors; Menin-OE significantly lowers glycolytic read-outs relative to NC (mean ± SD, *n* = 5). (E) Immunoblotting of xenograft lysates shows increased Menin and decreased HKDC1, GLUT1, and LDHA in the Menin-OE group; GAPDH serves as the loading control. (F) Immunohistochemical staining of xenograft sections for Menin, HKDC1, GLUT1, and LDHA at 200 × (left; scale bars, 100 μm) and 400 × (right; scale bars, 50 μm) magnification. At least one micrograph in each magnification group carries the indicated scale bar. Data are expressed as mean ± SD.∗*p* < 0.05; ∗∗*p* < 0.01; and ∗∗∗*p* < 0.001 versus NC. All *in vivo* experiments were performed with five biological replicates per condition.

Since the impact of Menin was evaluated in pancreatic cell lines, PL45 and Bx-PC3, we next investigated the impact of Menin *in vivo*. Leveraging Bx-PC3 cells, we initiated a xenograft model of PDAC in nude mice, employing both *Menin*-overexpressing cells and those transfected with the vector alone. A notable suppression of tumor growth was observed in the *Menin*-overexpressed group just seven days post-cell injection. Compared to the control group, *Menin* overexpressing cell line volume was significantly smaller than control group (Figure 7A), and the final tumor weight at sacrifice was consistent with this (Figures 7B and 7C). To further validate the Menin-YBX1-HKDC1 regulatory axis, we performed *HKDC1* knockdown experiments in our xenograft model. Both *Menin* overexpression and *HKDC1* knockdown significantly suppressed tumor growth compared to the control group, while the combined *Menin*-OE+sh*HKDC1* group exhibited the most pronounced tumor growth inhibition (Figures S5A - S5C). In consistent with this, ATP level, glucose consumption, and lactate production profile also resembles *in vitro* pattern (Figure 7D). HKDC1 and glycolysis marker genes (GLUT1 and LDHA) were also consistent with *in vitro* results using western blot (Figure 7E) and immunohistological staining (Figure 7F). Consistently, Menin and HKDC1 expression level in 10 paired PDAC samples was quantified and the genes were significantly differentially expressed and significantly correlated (Figures S6A–S6C). In summary, Menin suppression role in PDAC is reproducible *in vivo*.

### Menin predicted drug response and immune infiltration of PDAC

**Figure 8:**
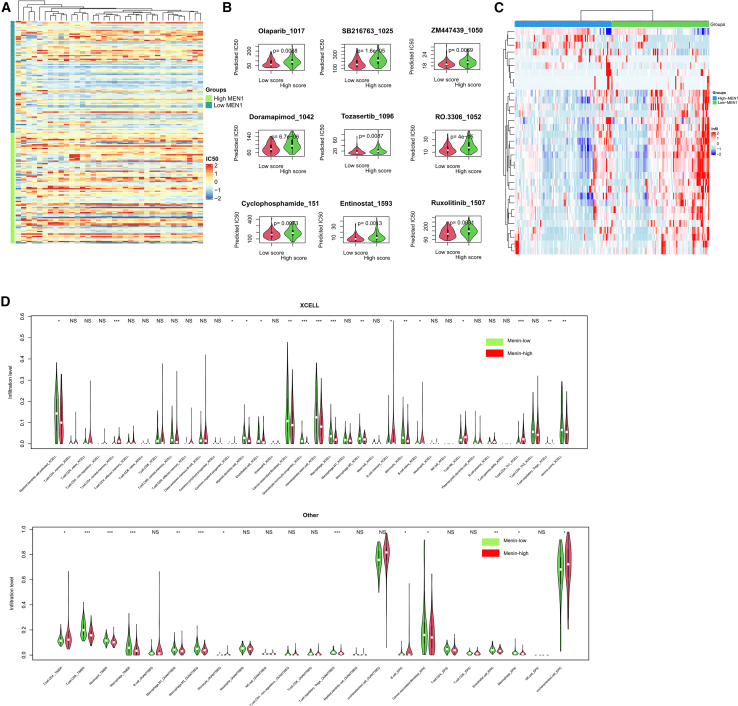
Infiltration and drug response correlated with Menin (A) Heatmap showing drugs with significantly (*p* < 0.01) differential IC50 values between Menin-low and Menin-high samples. (B) Detailed IC50 of some of these drugs. (C) Immune cells with differential infiltration abundance between Menin-low and Menin-high samples. (D) Detailed distribution of infiltration between groups.

To elucidate Menin’s potential role in PDAC therapeutic responsiveness, we integrated TCGA gene expression data with the oncopredict algorithm to generate predicted IC50 values. Correlation analysis revealed that low MEN1 expression potentially conferred enhanced sensitivity to multiple therapeutic agents (Figure 8A). Notably, several of these agents have established connections to glycolytic metabolism: Olaparib, a PARP inhibitor, has been shown to synergize with glycolysis inhibitors by exploiting metabolic vulnerabilities in cancer cells; doramapimod (p38 MAPK inhibitor) can modulate glycolytic enzyme expression and glucose metabolism through p38-mediated pathways; and ruxolitinib (JAK inhibitor) affects metabolic reprogramming by influencing STAT-mediated transcription of glycolytic genes (Figure 8B). Furthermore, immune infiltration patterns differed between Menin-low and Menin-high tumors, as shown in (Figures 8C and 8D).

These findings suggest that tumors with low Menin expression and enhanced glycolytic activity may be particularly vulnerable to therapeutic strategies that either target DNA repair mechanisms (exploiting the high replicative stress of glycolytic tumors) or directly interfere with metabolic signaling pathways. Experimental validation using PL45 and BxPC3 cells with stable Menin overexpression (MeninOE) or negative control vector (NC) confirmed these bioinformatic predictions, with MeninOE significantly decreasing sensitivity to these agents as evidenced by elevated IC50 values (Figure S7).

## Discussion

**Figure 9:**
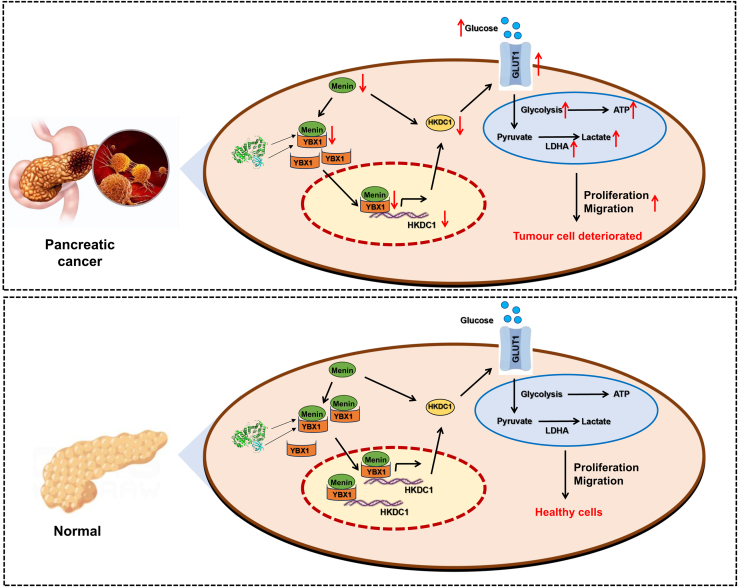
Mechanism of Menin regulating glycolysis via YBX/HKDC1 axes

In our investigation, we observed that heightened *Menin* expression significantly suppressed glycolysis in PDAC cells. This suppression was evidenced by decreased ATP and lactic acid outputs, as well as diminished expression of *GLUT1* and *LDHA*. Delving into the underlying mechanism, we found that *Menin* collaborated with *YBX1* to directly amplify the transcription of *HKDC1*, as shown in Figure 9. The binding was verified with multiple approaches. Notably, the glycolytic suppression resulting from Menin overexpression was partially mitigated when *HKDC1* was silenced. All those findings underscore the potential of *Menin* as a promising therapeutic target for PDAC.

An intriguing observation from our study is the apparent paradox wherein *Menin* overexpression simultaneously upregulates *HKDC1* while suppressing overall glycolytic activity. This counterintuitive finding suggests that *HKDC1*’s role in the *Menin*-regulated metabolic network extends beyond simple enzymatic enhancement of glucose phosphorylation. Several mechanisms may account for this phenomenon. First, while *HKDC1* upregulation may enhance the capacity for initial glucose phosphorylation, downregulation of the upstream glucose transporter *GLUT1* reduces glucose influx, limiting substrate availability for *HKDC1*. Concurrently, the more pronounced downregulation of the downstream glycolytic enzyme *LDHA* likely creates a metabolic bottleneck further along the pathway, collectively overriding *HKDC1*’s potential glycolysis-promoting effects. Second, the glucose-6-phosphate generated by enhanced *HKDC1* activity may be preferentially channeled toward alternative metabolic pathways such as the pentose phosphate pathway or glycogen synthesis, rather than proceeding through glycolysis. This metabolic flux redistribution could explain how increased *HKDC1* expression paradoxically contributes to overall glycolytic suppression within the Menin-regulated metabolic framework. The partial restoration of glycolysis upon *HKDC1* knockdown supports this interpretation, indicating that *HKDC1* functions as one component within a complex regulatory network rather than as an independent glycolytic driver.

The observation that *HKDC1* knockdown only partially restored glycolytic activity in Menin-overexpressing cells strongly suggests the existence of HKDC1-independent mechanisms through which Menin suppresses glucose metabolism. Indeed, our results demonstrate that *Menin* overexpression directly downregulates key glycolytic proteins GLUT1 and LDHA, effects that persist even when HKDC1 levels are manipulated. This indicates that Menin exerts broader metabolic control through multiple regulatory pathways beyond the YBX1-HKDC1 axis. Potential HKDC1-independent mechanisms may include Menin’s established role as an epigenetic regulator, where it could directly modulate the chromatin accessibility of glycolytic gene promoters through histone modifications. Additionally, Menin may influence upstream metabolic signaling cascades such as the PI3K/Akt pathway or AMPK signaling, both of which are master regulators of cellular glucose metabolism. Furthermore, Menin could regulate the expression or activity of other transcription factors that control glycolytic gene expression independently of the YBX1-HKDC1 pathway. These parallel regulatory mechanisms would explain why HKDC1 modulation provides only partial rescue of the Menin-induced glycolytic phenotype, highlighting the multifaceted nature of Menin’s metabolic regulatory functions.

*Menin*, produced by the *MEN1* gene, holds a pivotal position in numerous cellular functions associated with the pancreas, notably in pancreatic endocrine tumorigenesis and tumor progression.^5^^,^^34^ While the suppressive impact of *Menin* on metabolism in breast cancer and colon cancer is documented,^35^^,^^36^ its specific influence on PDAC tumor metabolism has not been fully characterized. Since glycolysis increasingly identified as a benchmark for the progression of pancreatic cancer,^11^^,^^37^ our research indicates that heightened *Menin* expression significantly diminishes glycolysis in PDAC cells through both *HKDC1*-dependent and *HKDC1*-independent mechanisms, underscoring *Menin*’s potential role as a comprehensive metabolic regulator.

Our drug sensitivity analysis provides clinical validation for *Menin*’s metabolic regulatory function, demonstrating that reduced *Menin* expression corresponds with heightened sensitivity to therapeutic agents that target glycolytic metabolism and associated stress response mechanisms. This observation aligns with our experimental evidence showing *Menin*’s suppression of glycolysis through the YBX1-HKDC1 axis and additional regulatory pathways. PARP inhibitors such as Olaparib exploit synthetic lethality in metabolically active tumors by amplifying replicative stress in cells already experiencing metabolic strain from enhanced glycolysis when *Menin* levels are diminished. The p38 MAPK and JAK signaling cascades directly modulate essential glycolytic enzymes and glucose transporters, indicating that tumors harboring dysregulated Menin-mediated glycolysis may develop heightened dependence on these survival pathways for metabolic adaptation. Low *Menin* expression thus creates a metabolic vulnerability that enables combination therapeutic approaches targeting both the elevated glycolytic state and compensatory DNA repair or signaling mechanisms in PDAC patients with reduced *Menin* levels. These observations reinforce *Menin*’s central role in metabolic regulation while suggesting that *Menin* expression status represents a viable biomarker for guiding personalized therapeutic strategies in PDAC management.

Previous investigations have documented *HKDC1*’s involvement in diverse malignancies, with studies demonstrating its participation in metabolic reprogramming processes across various cancer types.^38^ However, the functional outcomes of *HKDC1* activity appear to be highly context dependent, potentially varying based on the specific regulatory networks and cofactor interactions present within different cellular environments.^29^ Our investigation shows a mechanism wherein HKDC1 functions within the Menin-YBX1 transcriptional complex to modulate glycolytic processes in PDAC. Specifically, in the context of Menin overexpression, while the Menin-YBX1 complex upregulates *HKDC1*, the observed overall glycolytic suppression demonstrates that HKDC1’s enzymatic activity is subordinated to broader metabolic regulatory networks controlled by *Menin*. This finding expands our understanding of HKDC1’s regulatory repertoire and demonstrates that its metabolic impact can extend beyond simple enzymatic enhancement of glycolysis to encompass more sophisticated regulatory functions depending on its protein interaction partners and the cellular metabolic context.

*YBX1* functions as a versatile transcriptional regulator with demonstrated capacity for both gene activation and repression depending on the specific genomic context and associated cofactors.^39^^,^^40^ Our study reveals a functional interaction between YBX1 and Menin, through which a regulatory axis governs HKDC1 transcription and contributes to cellular glycolysis modulation as one component of Menin’s broader metabolic regulation. This finding illustrates how the same transcriptional regulator can participate in distinct functional outcomes when operating within different protein complex environments, highlighting the importance of cofactor interactions in determining *YBX1*’s ultimate biological impact within complex regulatory networks.

### Limitations of the study

In spite of garnering significant insights into the vital function of Menin in glycolysis in PDAC, our work has some limitations. First, our use of known 2D cancer cell lines (PL45 and Bx-PC3) and an immunocompromised nude xenograft model may not completely model the heterogeneous human tumor microenvironment, its three-dimensional structure, or the important host immune interactions. Therefore, the translational relevance of some findings, especially those concerning immune infiltration and predicted responses to therapy, will be limited. Secondly, though we clearly outlined the Menin-YBX1-HKDC1 pathway in controlling glycolysis, we need to further explore the upstream signals and post-translational modifications controlling the association between Menin and YBX1, its nuclear translocation, and the subsequent consequences. Thirdly, while we concentrated on glycolysis, the impact of Menin on other metabolistic pathways beyond glucose metabolism is broad and needs thorough exploration. Lastly, the therapeutic implications drawn from our preclinical observations, including the potentiality of Menin being an anticancer target, need strenuous translational verification in more biologically pertinent models like patient-derived organoids and thorough clinical trials in order for us to affirm its utility as an efficient prognostic or predictive biomarker in human PDAC patients.

## Resource availability

### Lead contact

Further information and requests for resources and reagents should be directed to and will be fulfilled by the lead contact, Peng Cheng (dfbbcxjh@163.com).

### Materials availability

This study did not generate new unique reagents.

### Data and code availability

• Single-cell RNA-seq data: The dataset generated during this study is available in the NCBI Gene Expression Omnibus (GEO) repository under the accession number GEO: GSE301126.
• Western blot data: All original, uncropped western blot images, along with a descriptive readme file, have been compiled into a single compressed archive (.zip) and are available as supplemental information (File S1).
• Graphical abstract created with BioRender.com (Created in BioRender. chang, w. (2025) https://BioRender.com/708xs6z).
• Additional information: Any additional information required to reanalyze the data reported in this paper is available from the lead contact upon reasonable request.

## Acknowledgments

This work was supported by the Natural Science Foundation of Shanghai (21ZR1478800) and the 10.13039/501100001809National Natural Science Foundation of China (81672352).

## Author contributions

Conceptualization, P.C., C.N., and Y.L.; methodology, P.C., C.N., J.Y., H.H., and Y.L.; investigation, C.N., J.Y., H.H., and Y.L.; formal analysis, C.N., H.M., X.S., J.Y., T.H., Y.Z., and Y.L.; visualization, C.N. and G.J.; writing—original draft, P.C., C.N., J.Y., Y.L., H.M., H.H., X.S., T.H., Y.Z., and G.J.; writing—review and editing, P.C., C.N., J.Y., Y.L., H.M., H.H., X.S., T.H., Y.Z., and G.J.; funding acquisition, P.C.; resources, P.C., C.N., and Y.L.; supervision, P.C., C.N., and Y.L.

## Declaration of interests

The authors declare no competing interests.

## STAR★Methods

### Key resources table

**Table:** 

REAGENT or RESOURCE	SOURCE	IDENTIFIER
**Antibodies**		

Anti-Menin	Proteintech	Cat#15159-1-AP; RRID:AB_2250607
Anti-GLUT1	Proteintech	Cat#21829-1-AP; RRID:AB_10837075
Anti-LDHA	Proteintech	Cat#19987-1-AP; RRID:AB_10646429
Anti-HKDC1	Proteintech	Cat#25874-1-AP; RRID:AB_2880279
Anti-YBX1	Proteintech	Cat#20339-1-AP; RRID:AB_10665424
Anti-GAPDH	Proteintech	Cat#60004-1-lg; RRID:AB_2107436
Goat Anti-Mouse IgG (HRP)	Abcam	Cat#ab205719; RRID:AB_2755049
Goat Anti-Rabbit IgG (HRP)	Abcam	Cat#ab6721; RRID:AB_955447

**Chemicals, peptides, and recombinant proteins**		

Lipofectamine 2000	Invitrogen, USA	11668019
Trizol reagent	Thermo	15596018
DMEM medium	Merck	FG0445-BC
Fetal bovine serum (FBS)	Gibco, USA	A5670701
Penicillin-Streptomycin	Invitrogen, USA	15140148
2-deoxy-d-glucose (2-DG)	Sigma–Aldrich	D8375
RIPA buffer with protease inhibitors	Thermo	89900

**Critical commercial assays**		

Universal Mycoplasma Detection Kit	ATCC	30-1012K
PowerPlex 21 HS System	Promega Corporation	DC8902
Reverse transcription kit	TianGen	GKR107-02
ATP assay kit	Beyotime	S0026
L-lactate assay kit	Beyotime	P0393S
Glucose Uptake Assay Kit	Beyotime	S0201S
ChIP kit	Merck Millipore	17-295
Pierce Direct Magnetic IP/Co-IP kit	Thermo Scientific	88828
NEBNext Ultra™ RNA Library Prep Kit	NEB	E7770S

**Deposited data**		

RNA-seq data	GEO: GSE301126	

**Experimental models: Cell lines**		

PL45	iCell Bioscience Inc.	iCell-h175
Bx-PC3	ATCC	CRL-1687
HEK-293	ATCC	CRL-1573

**Experimental models: Organisms/strains**		

BALB/c nude mice (BALB/c-nu/nu)	Shanghai SLAC Laboratory Animal Center	N/A

**Recombinant DNA**		

sh-HKDC1	GenePharma	5’-CCCTCGATGTGATGTGACATT-3’
sh-YBX1	GenePharma	5’-CAGTTCAAGGCAGTAAATAT-3’

**Software and algorithms**		

GraphPad Prism	GraphPad Software	N/A
SPSS	IBM	N/A
GSEA software	Broad Institute	N/A
OmicShare tool	www.omicshare.com/tools	N/A

**Other**		

Seahorse XF24 Analyzer	Seahorse Bioscience	N/A
ABI7500 PCR System	Applied Biosystems Inc.	N/A
Agilent 2100 bioanalyzer	Agilent	N/A
cBot Cluster Generation System	Illumina	N/A

### Experimental model and study participant details

#### Cell lines

The pancreatic ductal adenocarcinoma (PDAC) cell lines PL45 and Bx-PC3 were obtained from iCell Bioscience Inc. (Shanghai, China) and the American Type Culture Collection (ATCC; Manassas, VA, USA), respectively. Both were stored in liquid nitrogen at the Research Center of Changhai Hospital.Cell line authentication was performed using short tandem repeat (STR) profiling analysis. Genomic DNA was extracted from cultured cells and analyzed for 21 STR loci using the PowerPlex 21 HS System (Promega Corporation). STR profiles were compared against reference databases including ATCC, DSMZ, and JCRB to confirm cell identity. All cell lines showed >90% concordance with reference profiles, confirming their authenticity.

Mycoplasma contamination testing was conducted biannually using a PCR-based detection method with the Universal Mycoplasma Detection Kit (ATCC 30-1012K). The assay targets conserved 16S rRNA gene sequences present in all mycoplasma species. All cell lines tested negative for mycoplasma contamination throughout the study period.

Cells were maintained in DMEM medium (Merck) supplemented with 10% fetal bovine serum (Gibco, USA), 1% penicillin (100 U/mL), and 1% streptomycin (100 μg/mL) (Invitrogen, USA) at 37°C in a humidified atmosphere containing 5% CO2. Only cells within their first 20 passages were used for experiments to maintain consistent cellular characteristics.

#### Animal models

Four-week-old male BALB/c nude mice (BALB/c-nu/nu) were purchased from Shanghai SLAC Laboratory Animal Center, Chinese Academy of Sciences (Shanghai, China). Mice were housed in individually ventilated cages under specific pathogen-free (SPF) conditions with a 12-hour light/dark cycle, controlled temperature (22 ± 2°C), and humidity (50-60%). Animals had free access to autoclaved standard laboratory chow and water. All animal procedures were approved by the Institutional Animal Care and Use Committee (Approval No: CHEC2023-009) and conducted in accordance with institutional guidelines for animal care and use, following the principles of the 3Rs (Replacement, Reduction, Refinement).

Male mice were specifically chosen to eliminate potential sex-related hormonal influences on tumor growth, as previous studies have shown that estrogen can affect pancreatic cancer progression. Sex as a biological variable was not considered a confounding factor in this study, as the focus was on the molecular mechanisms of Menin regulation rather than sex-specific differences in tumor biology.

For xenograft experiments, mice were randomly assigned to experimental groups (n=5 per group) and monitored daily for signs of distress. For these experiments, PL45 cell groups (1×10ˆ7 cells in 200 μl PBS) were injected subcutaneously into the flank of sedated mice. Tumor measurements were performed every 4 days using digital calipers, with their volume determined by the equation: volume (mmˆ3) = 0.5×length×widthˆ2. Mice were humanely euthanized when tumors reached predetermined ethical endpoints or at the conclusion of the 28-day experimental period via CO2 asphyxiation followed by cervical dislocation to ensure death. The excised tumors were then weighed and further utilized for Western blot detection.

#### Human tissue samples

The 10 pairs of pancreatic ductal adenocarcinoma (PDAC) tissues and matched adjacent non-tumorous tissues used for the correlation analysis in Figure S6 were selected from a previously established patient cohort.^4^ This cohort, collected at Changhai Hospital (affiliated with the Second Military Medical University of Shanghai, China) between July 2010 and June 2012, has been described previously.^4^

The collection and use of these samples were approved by the Ethics Committee of the Second Military Medical University of Shanghai, China. Written informed consent was obtained from all patients prior to sample collection. All procedures involving human samples were conducted in accordance with the principles of the Declaration of Helsinki.

### Method details

#### Cell transfection and immunofluorescence

The overexpression plasmid for Menin/YBX1 and its control counterpart, an empty pcDNA3.1 plasmid, were sourced from GenePharma (Shanghai, China). Short hairpin RNAs (shRNAs) targeting HKDC1 and the respective negative control fragments were procured from Genechem. For transient transfection procedures, PDAC cells were seeded at a concentration of 5 × 10ˆ5 cells/well in 6-well plates and allowed to adhere for 24 hours. Subsequently, cells were transfected using Lipofectamine 2000 reagent (Invitrogen, USA) following the manufacturer's guidelines, employing the relevant plasmids. Post-transfection, the efficiency of either knockdown or overexpression was validated before proceeding to subsequent experiments.

Seed cells onto precoated slides in culture dishes, wash twice with PBS by centrifugation at 1000 rpm for 5 minutes. Chill the plate on ice, aspirate the culture medium, wash thrice with cold PBS at 4°C for 5 minutes each, aspirate residual PBS, air-dry, and fix cells in fixative for 30 minutes. Permeabilize cells before antibody incubation to allow antibody access to antigens. Block cells with blocking solution for 30 minutes. Incubate with the primary antibody at room temperature for 1 hour or overnight at 4°C. Wash thrice with BSA, 5 minutes each. Incubate with the secondary antibody required for immunofluorescence in the dark at room temperature for 1 hour. Wash thrice with BSA and once with distilled water, 5 minutes each. Stain nuclei by adding DAPI in the dark for 5 minutes. Mount using an aqueous mounting medium and photograph.

#### Quantitative reverse transcription PCR (qRT-PCR)

Total RNA was isolated from cells using the Trizol reagent (Thermo) following the recommended manual. After quantity of extracted RNA assessed spectrophotometrically, cDNA synthesis was performed using a reverse transcription kit (TianGen). QRT-PCR was carried out with the Taq Pro Universal SYBR qPCR Master Mix (Vazyme) on an ABI7500 PCR System (Applied Biosystems Inc., USA). Gene expression was quantified using the 2ˆ−ΔΔCT method, with GAPDH acting as the internal reference. The primers used for GAPDH, Menin, AKR1B10, ARSB, FBP1, HKDC1, TYMP, YBX1, GLUT1 and LDHA are provided in Table S1.

#### Western blot

Cellular proteins were extracted using RIPA buffer supplemented with protease inhibitors (Thermo). The lysates were then subjected to SDS-PAGE and electrotransferred to PVDF membranes. These membranes were subsequently blocked using 5% non-fat milk. Primary antibodies targeting Menin (1:2000, 15159-1-AP, Proteintech), GLUT1 (1:4000, 21829-1-AP, Proteintech), LDHA (1:5000, 19987-1-AP, Proteintech), HKDC1 (1:1000, 25874-1-AP, Proteintech), YBX1 (1:1000, 20339-1-AP, Proteintech) and GAPDH (1:30000, 60004-1-lg, Proteintech) were applied and the membranes were incubated overnight at 4°C. Following a trio of washes in 1% TBST buffer, the membranes were treated with the secondary antibody, Goat Anti-Mouse IgG (HRP) (1:3000, ab205719, Abcam) or Goat Anti-Rabbit IgG (HRP) (1:3000, ab6721, Abcam), for 2 hours at ambient temperature. Protein bands were visualized using an enhanced chemiluminescence kit (Thermo).

#### Measurement of ATP and lactate

ATP concentrations were assessed utilizing an ATP assay kit (Cat No. S0026) from Beyotime. In brief, following transfection with shRNA, the lysates from PL45 and Bx-PC3 cells were centrifuged at 12,000 g for a duration of 10 minutes. A 50 μl portion of the resultant supernatant was then combined with 100 μl of the ATP detection working solution. Subsequently, luminescent readings were taken using the Centro LB960 microplate luminescence detector from Berthold, Germany. ATP levels were deduced based on the established standard curve.

Lactate estimation for conditioned media of transfected cells compared to control cells was performed using L-lactate assay kit (Beyotime, Shanghai) as per manufacturer’s guidelines.

#### GLUCOSE uptake

A glucose uptake assay was carried out utilizing a Glucose Uptake Assay Kit from Beyotime (S0201S) as per the manufacturer's guidelines. In a nutshell, cells (1x10ˆ5) were plated in 24-well plates and incubated at 37°C in a 5% CO2 atmosphere for 24 hours. To enhance glucose uptake, cells were subsequently deprived of serum and incubated overnight. The following day, cells underwent three washes with 1xPBS, then subjected to glucose starvation by treatment with Krebs-Ringer bicarbonate 4-(2-hydroxyethyl)-1-piperazineethanesulfonic acid HEPES (KRBH) buffer supplemented with 2% BSA for 40 minutes. Subsequently, 1 mM 2-deoxy-D-glucose (2-DG) was introduced to the cells for an additional 20-minute incubation. Post-incubation, cells were harvested, and the uptake of 2-DG was assessed.

#### ECAR and OCR

The Extracellular Acidification Rate (ECAR) in mpH/min and the Oxygen Consumption Rate (OCR) in pmol/min were ascertained utilizing a Seahorse XF24 Analyzer (Seahorse Bioscience, North Billerica, MA) in adherence to the provided guidelines from the manufacturer. Transfected cells, after achieving a 70-80% confluence in complete RPMI 1640 medium, were detached using trypsin and seeded at a density of 2.0 × 10ˆ4 cells in each well (with a volume of 100 μL) on an XF24 cell culture microplate. This seeding occurred 24 hours before the scheduled assay. The growth medium was then substituted with XF assay medium, followed by a 1-hour incubation at 37°C in a CO2-free environment before commencing the assay. Subsequently, the microplates were positioned in the XF24 analyzer. The ECAR measurements were taken before and post the addition of 2-deoxy-d-glucose (2-DG). These readings were consistently captured at predetermined intervals. Both the medium and 2-DG were sourced from Sigma–Aldrich (St. Louis, MO), while the additional reagents were procured from Seahorse Bioscience.

#### RNA-seq and analysis

Menin-modulated PL45 cells were harvested and RNA was extracted using the TRIzol reagent (Invitrogen), adhering to the manufacturer's guidelines. Following RNA integrity assessment via the Agilent 2100 bioanalyzer, the isolated RNA was reverse-transcribed to establish a cDNA library utilizing the NEBNext Ultra™ RNA Library Prep Kit, preparing it for subsequent sequencing endeavors. Index-coded sample clustering was achieved on a cBot Cluster Generation System with the TruSeq PE Cluster Kit v3-cBot-HS (Illumina), again according to manufacturer directives. Paired-end sequencing (150 bp) was executed by Chi-Biotech, based in Shenzhen, China. For robustness, three biological replicates were processed for each cell sample. Data-driven analyses like the Gene Set Enrichment Analysis (GSEA) and the Kyoto Encyclopedia of Genes and Genomes (KEGG) pathway exploration were carried out using the OmicShare tool.

#### Gene Set Enrichment Analysis (GSEA) of Menin

Samples from the TCGA-PAAD cohort were categorized into two groups based on Menin expression levels: the Menin-high group and the Menin-low group, using the median expression value of Menin as a threshold. Gene Set Enrichment Analysis (GSEA) was executed using the GSEA software (version 4.0.2) to contrast these two groups. The reference dataset labeled as c2.cp.kegg.v7.4.symbols.gmt was used as the baseline, setting the number of permutations at 1000. Significantly enriched pathways adhered to the following criteria: a false discovery rate (FDR) below 0.25, an absolute normalized enrichment score (NES) exceeding 1, and a nominal p-value (NOM p-value) below 0.05.

#### IP -MS

Immunoprecipitation assays were conducted utilizing the Pierce Direct Magnetic IP/Co-IP kit (Thermo Scientific, Rockford, IL, USA) as per the manufacturer's guidelines. In essence, cells were disrupted using the IP lysis buffer, followed by protein quantification. Beads, pre-bound with 5 μg of Menin or YBX1 antibody, were mixed with 500-μl lysis solutions containing protein concentrations between 1–2 mg/ml. This mixture was incubated overnight at 4°C. The subsequent eluted proteins were directed to LC–MS/MS analysis, facilitated by PTM Biolabs, China. For negative control purposes, IgG from Proteintech was employed.

#### CHIP

The Chromatin Immunoprecipitation (ChIP) assay was carried out on cells with modulated expression of Menin or YBX1, utilizing a ChIP kit from Merck Millipore (Cat No. 17-295), adhering to the manufacturer's recommended procedure. Comparable quantities of immuno-precipitate and input DNA were subjected to ChIP–PCR under the specified conditions: an initial denaturation at 95°C for 30 seconds, primer-specific annealing for 30 seconds, and extension at 72°C for 20 seconds across 30 cycles.

### Quantification and statistical analysis

All experiments were performed at least three independent times (n=3 biological replicates for *in vitro* studies, n=5 mice for *in vivo* xenograft models, and n=10 paired patient samples for clinical correlations), unless otherwise stated. Statistical significance between groups was determined using GraphPad Prism 7.0 and SPSS 22.0 software packages. For comparisons between two groups, a two-tailed Student's t-test was applied. For comparisons involving more than two groups, Analysis of Variance (ANOVA) was utilized. All quantitative data are presented as the mean ± standard deviation (SD). The significance level for all analyses was consistently set at P < 0.05. All specific statistical details, including statistical tests used, exact value of n, what n represents, definition of center, and dispersion and precision measures, are provided in the corresponding figure legends, figures, and the results section of the manuscript.

## Supplemental information

Document S1. Figures S1–S7 and Table S1

Data S1. Raw WB Figures 5, 3F, 7E, and S3

File S1. Western Blot Data
