# Supplementary material for: Menin regulates YBX1 nucleus translocation to boost the HKDC1 transcription and affects pancreatic cancer glycolysis
Source: iScience. 2025 Aug 7;28(9):113245. doi: 10.1016/j.isci.2025.113245 (PMC12396302; doi:10.1016/j.isci.2025.113245)
Supplement: Document S1. Figures S1–S7 and Table S1 [file mmc1.pdf]

iScience, Volume *28*

Supplemental information

Menin regulates YBX1 nucleus translocation to boost the HKDC1 transcription and affects pancreatic cancer glycolysis

Chenming Ni, Jiacheng Yang, Yebin Lu, Hongyun Ma, Hao Hu, Xiaohan Shi, Tianlin He, Yijie Zhang, Gang Jin, and Peng Cheng

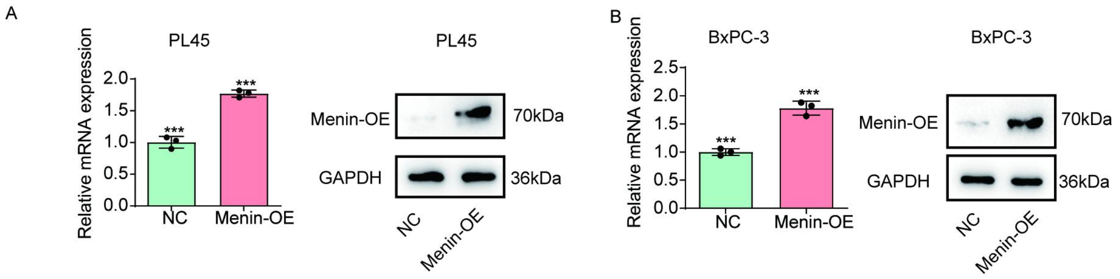

Figure S1. Menin overexpression is successfully established in pancreatic cancer cell lines, related to Figure 1

PL45 and BxPC-3 cells were transfected with Menin-overexpressing (Menin-OE) or control (NC) vectors. **A, B** qRT-PCR and Western blot validation of Menin overexpression in PL45 (A) and BxPC-3 (B) cells. GAPDH served as loading control. Data represent mean ± SD (n=3). ***P < 0.001 by two-tailed t-test.

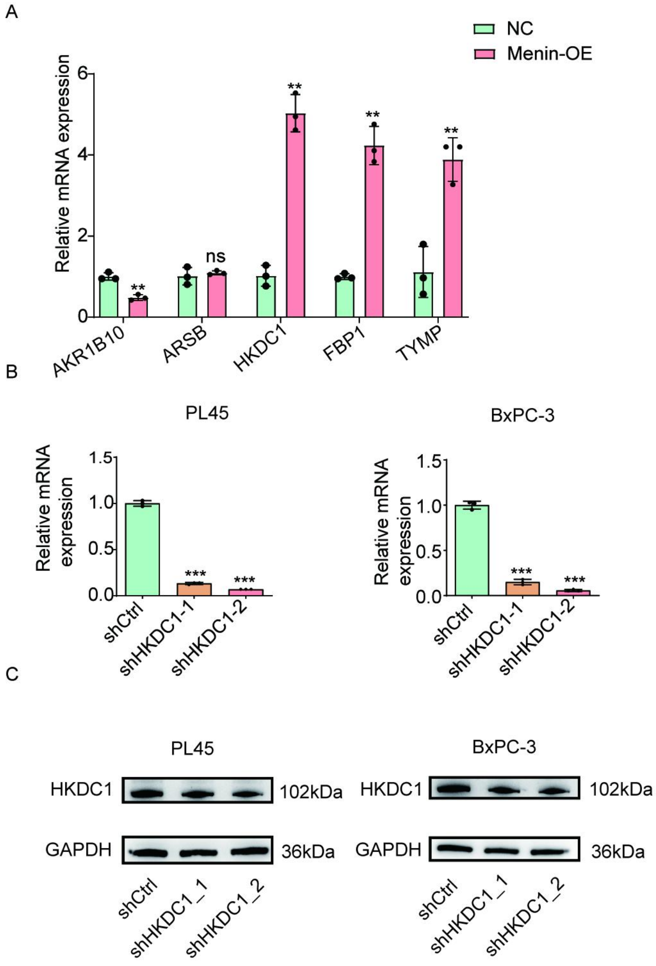

Figure S2. Menin upregulates HKDC1 expression in pancreatic cancer cells, related to Figure 3

A. qRT-PCR analysis of Menin downstream targets in cells transfected with NC or Menin-OE vectors. **B.** HKDC1 mRNA expression following transduction with control shRNA (shCtrl) or HKDC1-targeting shRNAs. **C.** Western blot analysis of HKDC1 protein levels with GAPDH as control. Data represent mean ± SD (n=3). **P < 0.01, ***P < 0.001 by two-tailed t-test

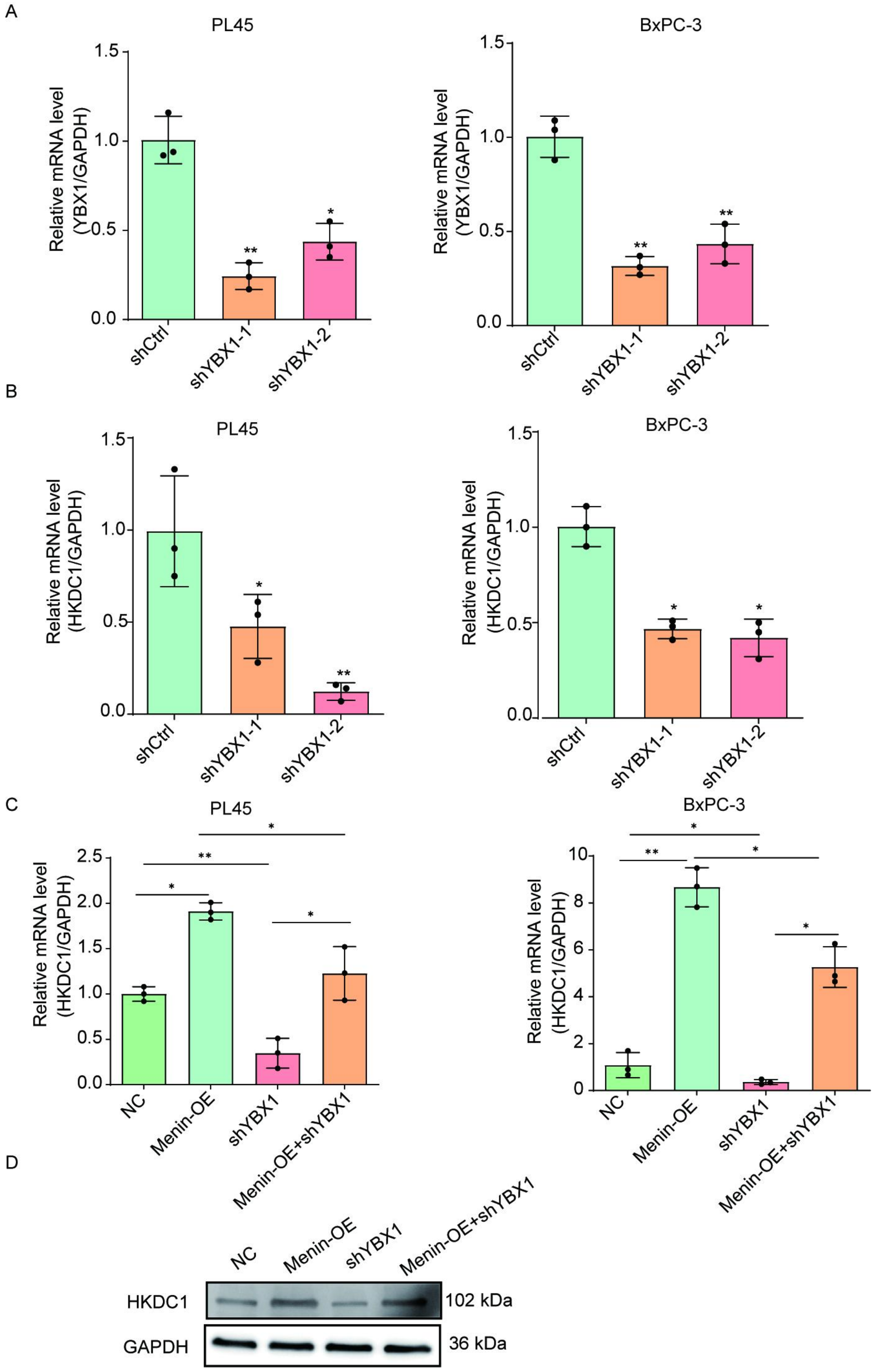

Figure S3. YBX1 mediates Menin-induced HKDC1 upregulation, related to Figure 6

A. YBX1 mRNA levels following transduction with control or YBX1-targeting shRNAs. **B.** HKDC1 mRNA expression after YBX1 knockdown. **C.** HKDC1 mRNA levels in NC, Menin-OE, YBX1 knockdown (shYBX1), and combined treatment groups. **D.** Western blot of HKDC1 protein levels with GAPDH as control. Data represent mean ± SD (n=3). $*P < 0.05$, $**P < 0.01$ by two-tailed t-test.

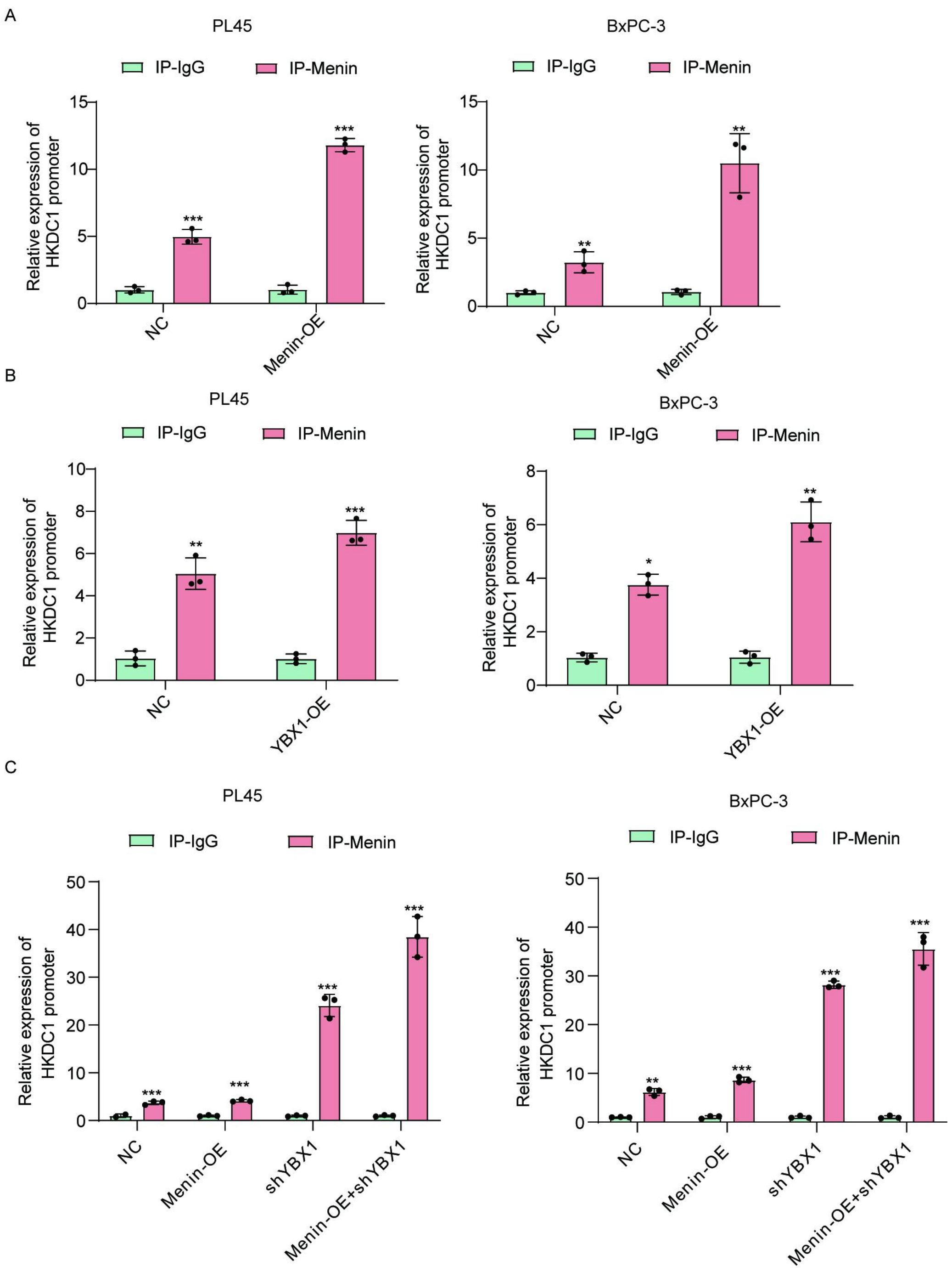

Figure S4. Menin directly binds the HKDC1 promoter through YBX1-dependent mechanism, related to Figure 6

ChIP-qPCR analysis of Menin enrichment at HKDC1 promoter using specific Menin antibody versus IgG control. **A.** Menin binding in NC and Menin-OE cells. **B.** Menin binding in NC and YBX1-overexpressing cells. **C.** Menin binding across treatment groups including combined Menin-OE and YBX1 knockdown. Data represent mean ± SD (n=3). *P < 0.05, **P < 0.01, ***P < 0.001 by two-tailed t-test.

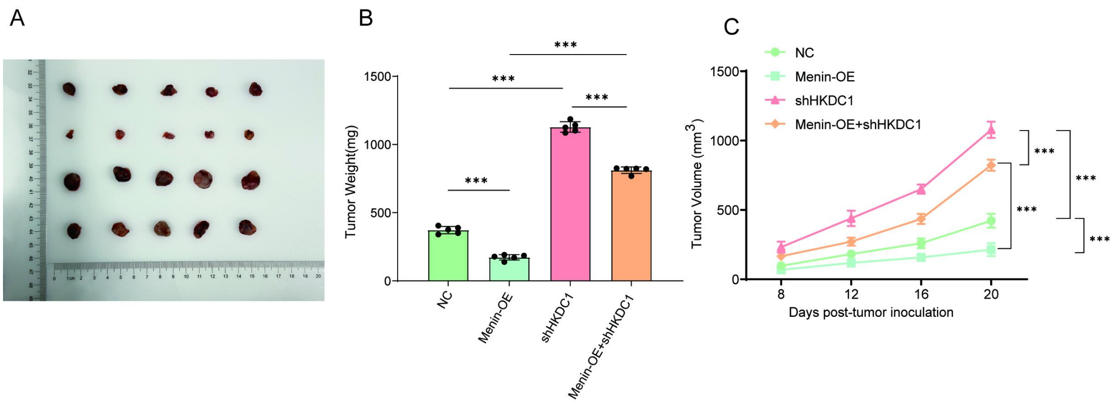

Figure S5. HKDC1 knockdown partially reverses Menin-mediated tumor suppression, related to Figure 7

Nude mice were inoculated with cells expressing NC, Menin-OE, shHKDC1, or combined constructs (n=5 per group). **A.** Representative tumor images at study endpoint. **B.** Final tumor weights. **C.** Tumor growth curves over time. Data represent mean ± SD. ***P < 0.001 by two-tailed t-test.

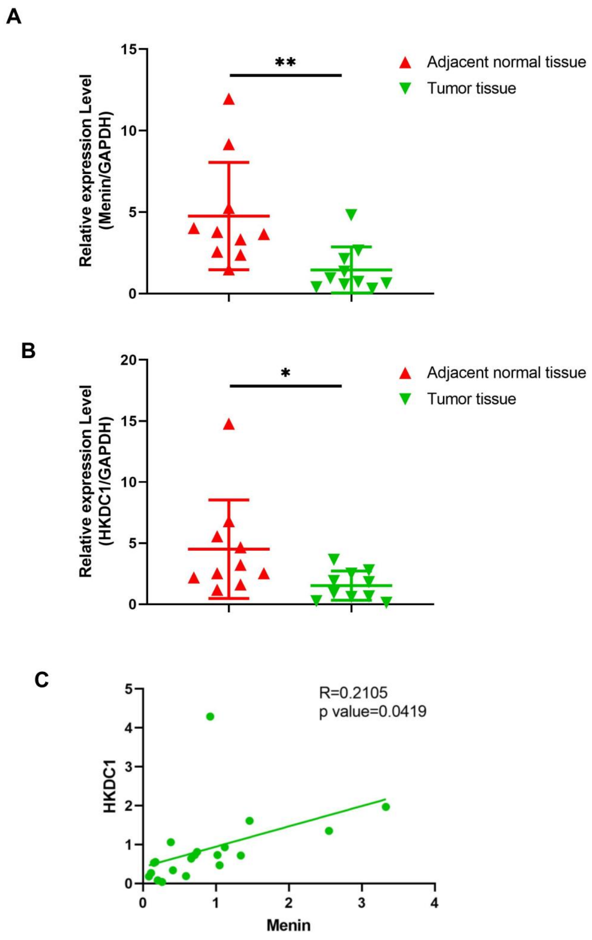

Figure S6. Menin and HKDC1 expression correlate in pancreatic cancer tissues, related to Figure 7

qRT-PCR analysis of 10 paired pancreatic tumor and normal tissues. **A, B.** Relative mRNA

expression of Menin (A) and HKDC1 (B) in tumor versus normal tissues. **C.** Correlation analysis between Menin and HKDC1 expression (n=20 samples). Pearson correlation coefficient and p-value indicated. Data show individual values with mean ± SD. *P < 0.05, **P < 0.01 by paired t-test.

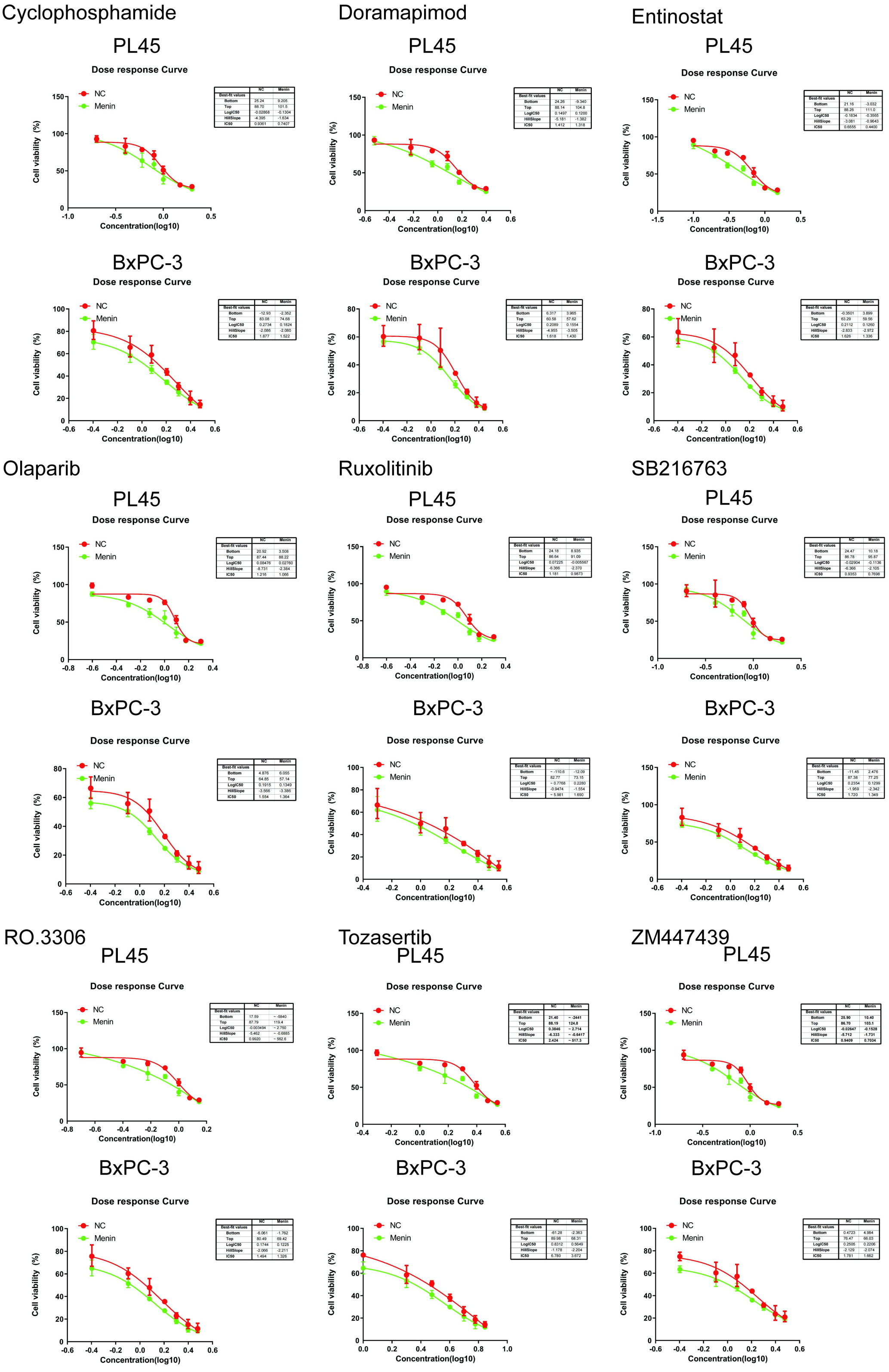

Figure S7. Menin expression modifies drug sensitivity profiles,related to Figure 8

PL45 and BxPC-3 cells expressing NC or Menin were treated with serial dilutions of nine compounds. Dose-response curves show cell viability following 72-hour treatment with indicated drugs. IC50 values provided in inset tables. Data represent mean ± SD (n=3).

Table S1: Primer list for qPCR analysis

Primer Name	Primer（5'-3')
MEN1_F:	CAGAGTGAGAAGATGAAGGG
MEN1_R:	CTTGAGGAAAGACAGAGTGT
AKR1B10 _F:	CCAGAGGAATGTGATTGTCA
AKR1B10 _R:	CCAAATGAGAGGATTGCAAC
ARSB_F:	CTGTGTTCCTCTGGATGAAA
ARSB_R:	CACTACCCAGGAGATATCCA
FBP1_F:	ACTGAGTACATCCAGAGGAA
FBP1_R:	AAATATCCCTCCGTAGACCA
HKDC1_F:	AAGCAACTGTTCGAGAAGAT
HKDC1_R:	CAGAAGATTTCTCACCACCA
TYMP_F:	ATTCAATGTCATCCAGAGCC
TYMP_R:	CACATCTCTGGCTGCATATA
LDHA_F:	GAGGTTCACAAGCAGGTGGT
LDHA_R:	GTGCACCCGCCTAAGATTCT
GLUT1_F:	AGTCCTTTGAGATGCTGATC
GLUT1_R:	GACACTTCACCCACATACAT
GAPDH_F:	GATTCCACCCATGGCAAATTC
GAPDH_R:	CTGGAAGATGGTGATGGGATT
