# Supplementary material for: Menin regulates YBX1 nucleus translocation to boost the HKDC1 transcription and affects pancreatic cancer glycolysis
Source: iScience. 2025 Aug 7;28(9):113245. doi: 10.1016/j.isci.2025.113245 (PMC12396302; doi:10.1016/j.isci.2025.113245)

Figure 3F

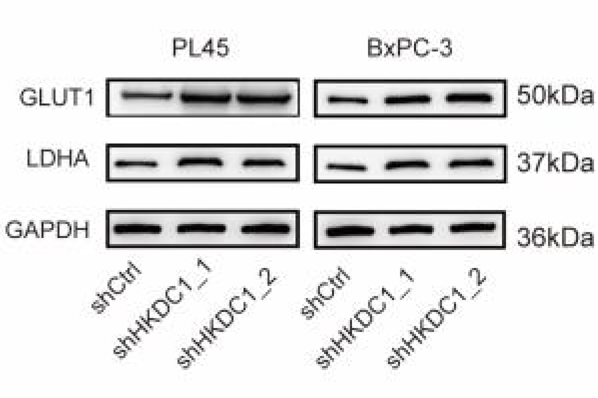

PL45

GLUT1

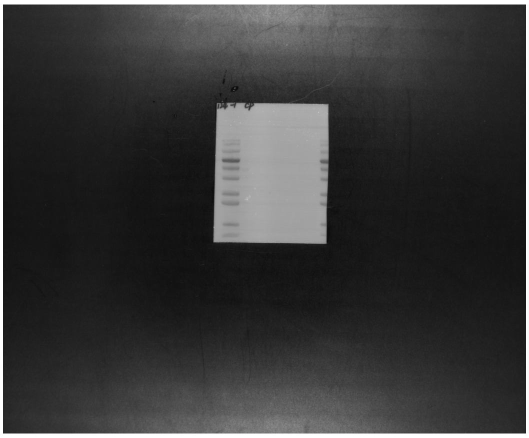
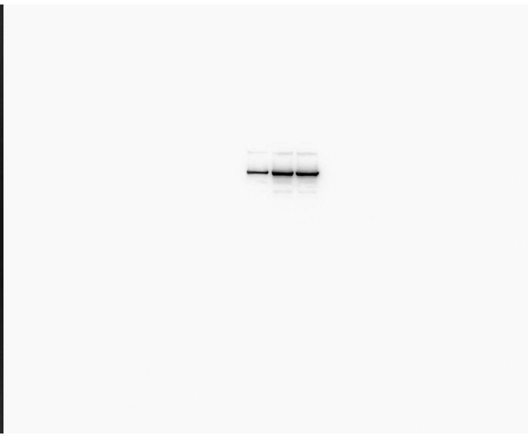

LDHA

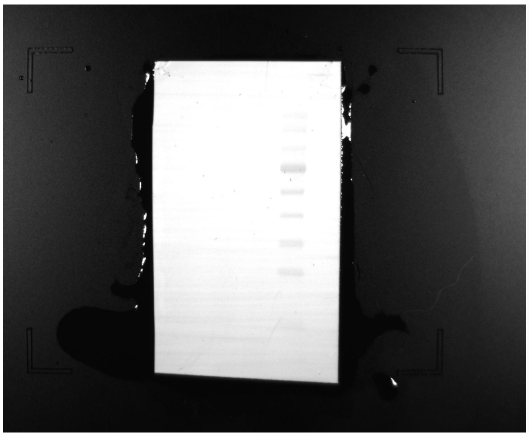
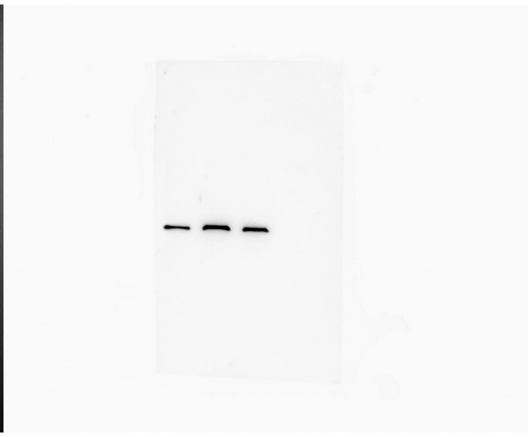

GAPDH

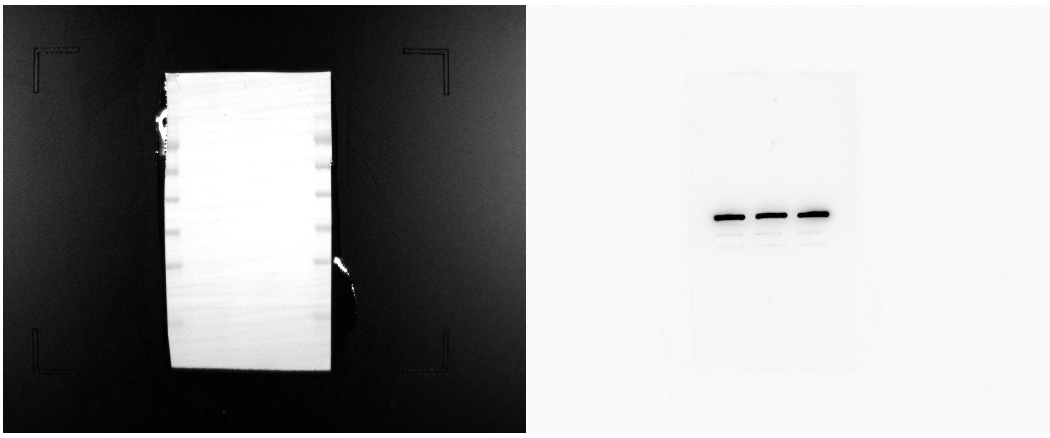

BxPC-3
GLUT1

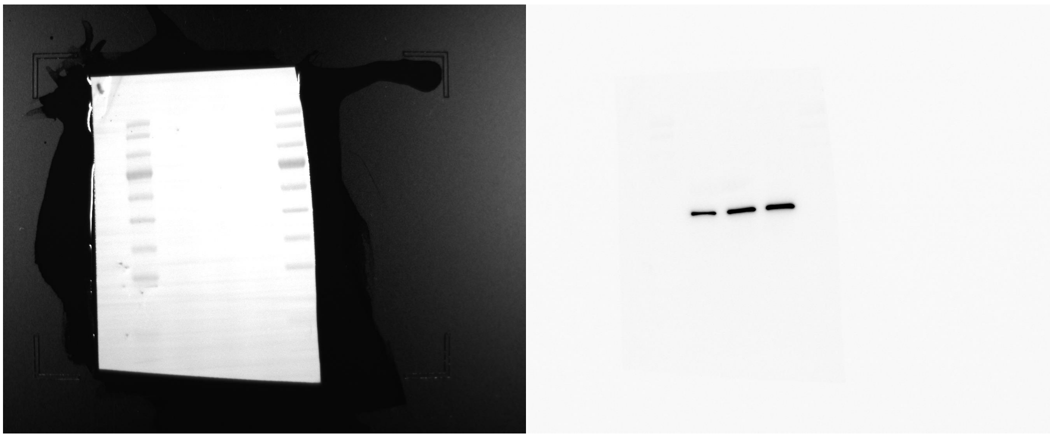

LDHA

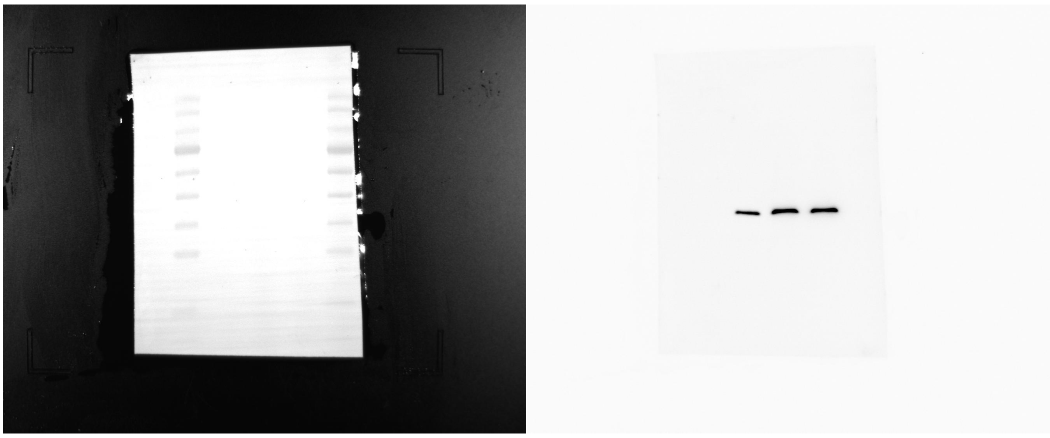

GAPDH

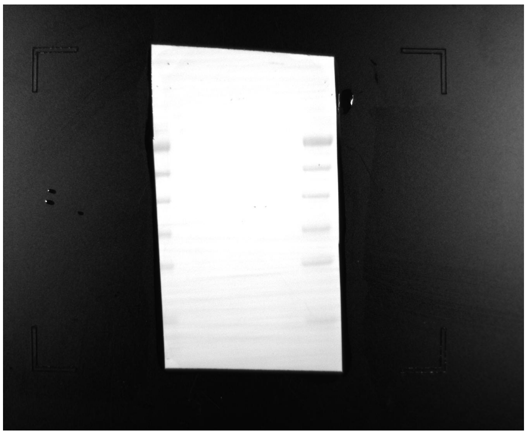

Figure 5D

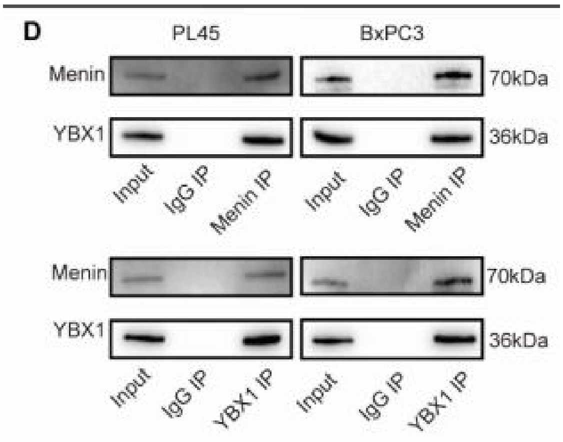

PL45

Menin IP

Menin

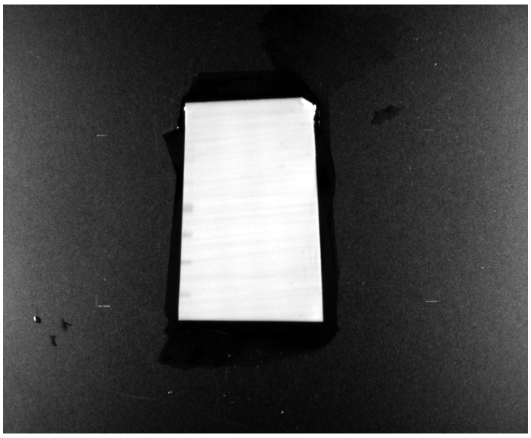

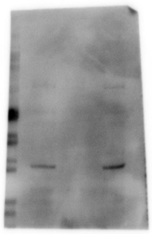

YBX1

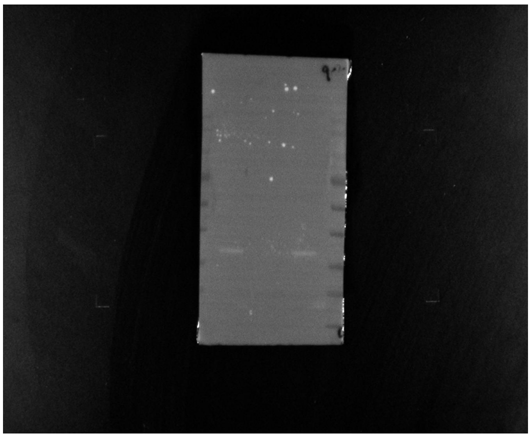

YBX1 IP

Menin

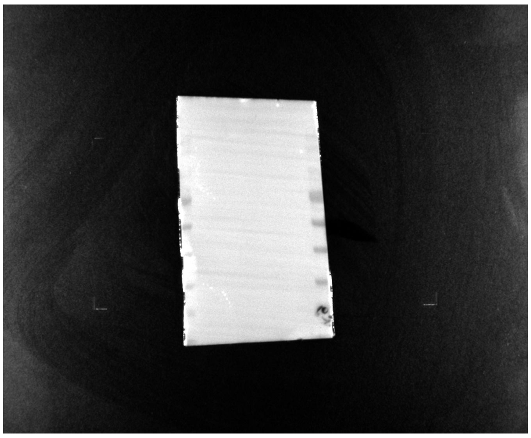

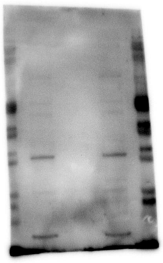

YBX1

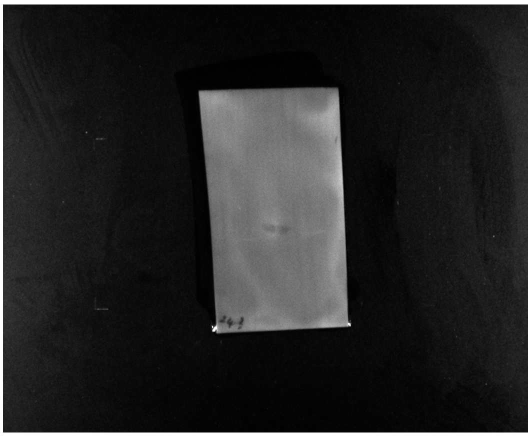

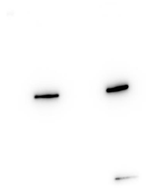

BxPC3

Menin IP

Menin

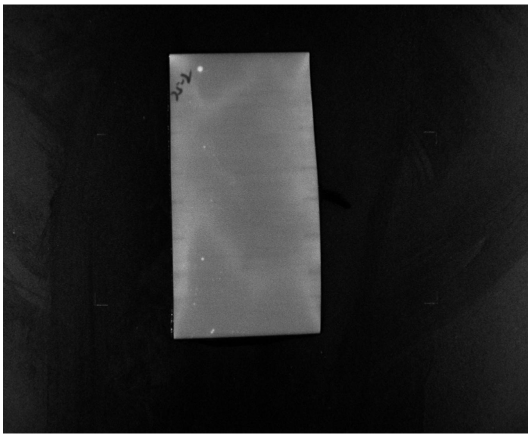

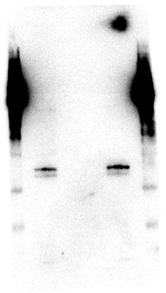

YBX1

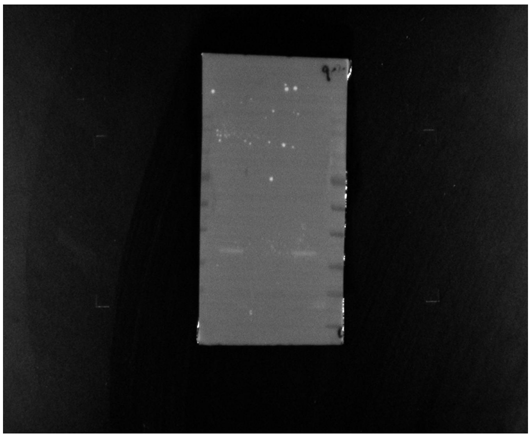

YBX1 IP

Menin

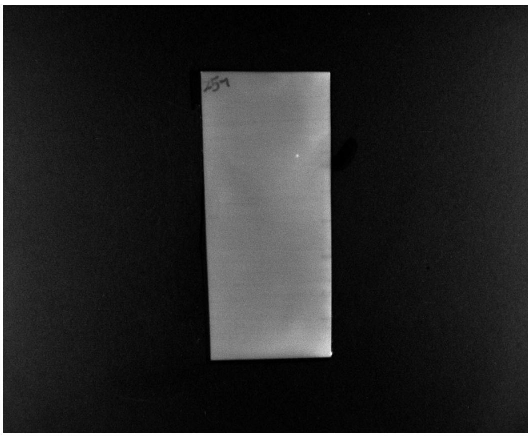

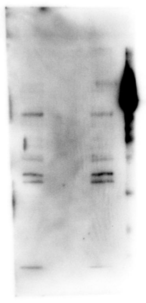

YBX1

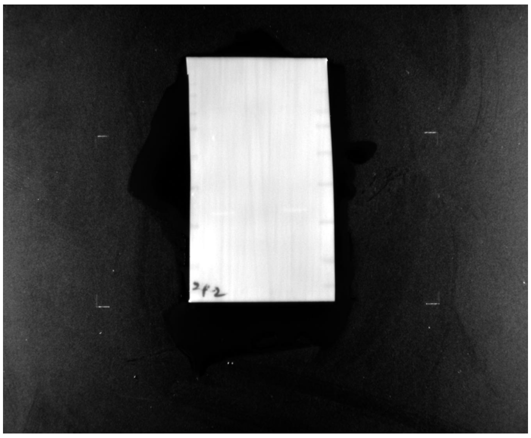

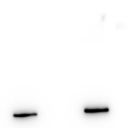

Figure 5F

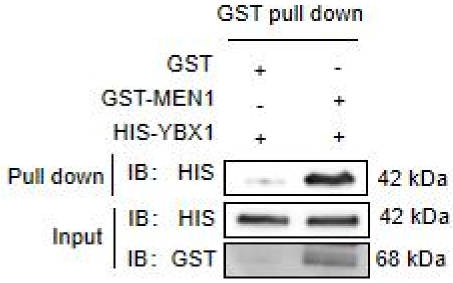

Pulldown

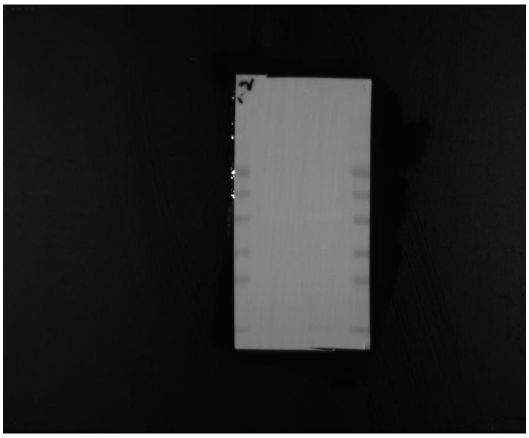

Input

IB:HIS

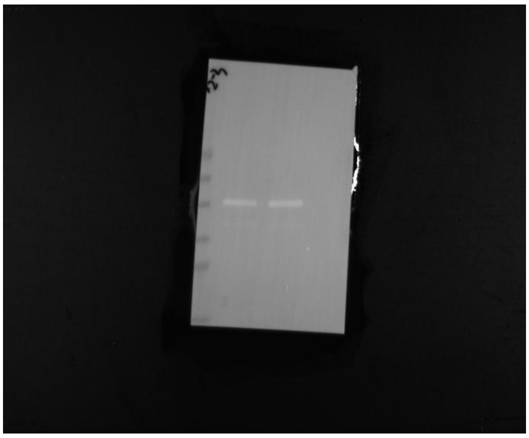

IB:GST

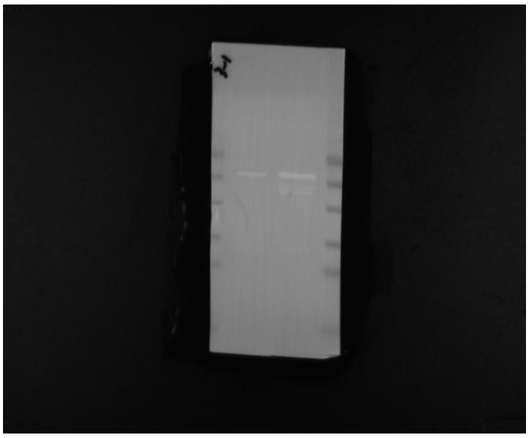

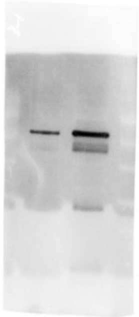

Figure 5F

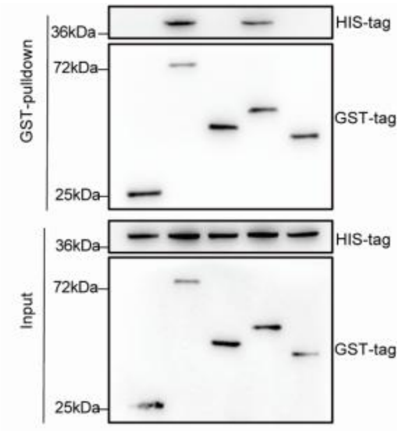

GST pull-down

GST tag

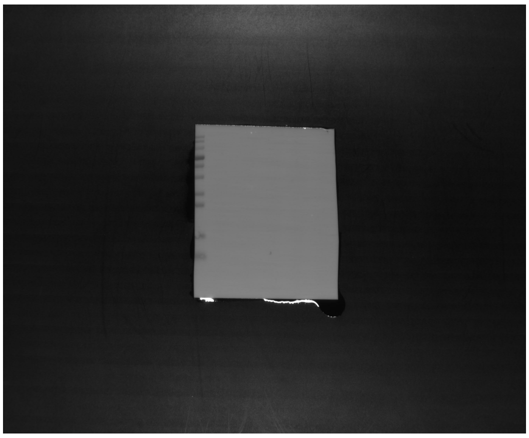
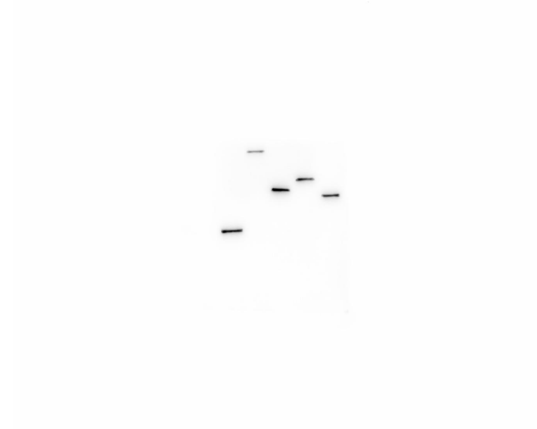

HIS-tag

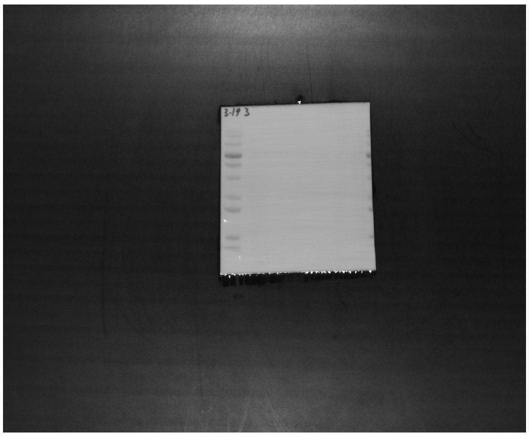
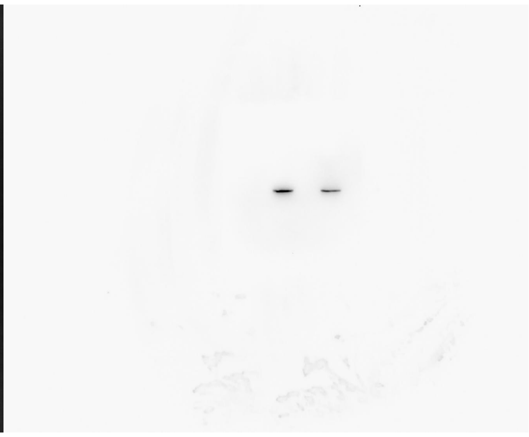

Input
GST tag

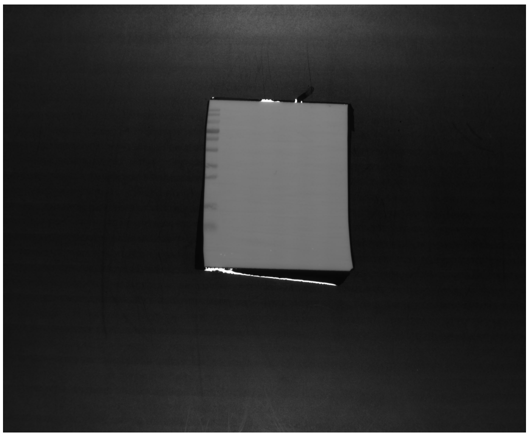
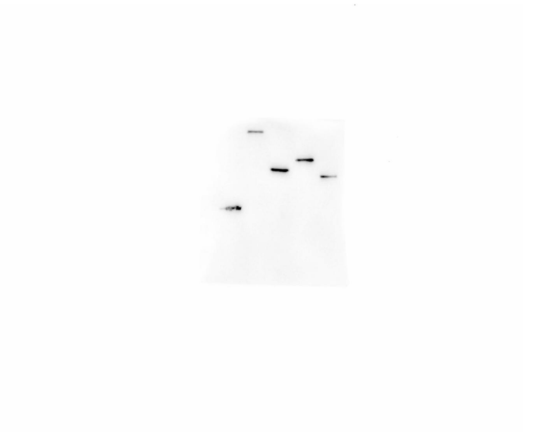

HIS-tag

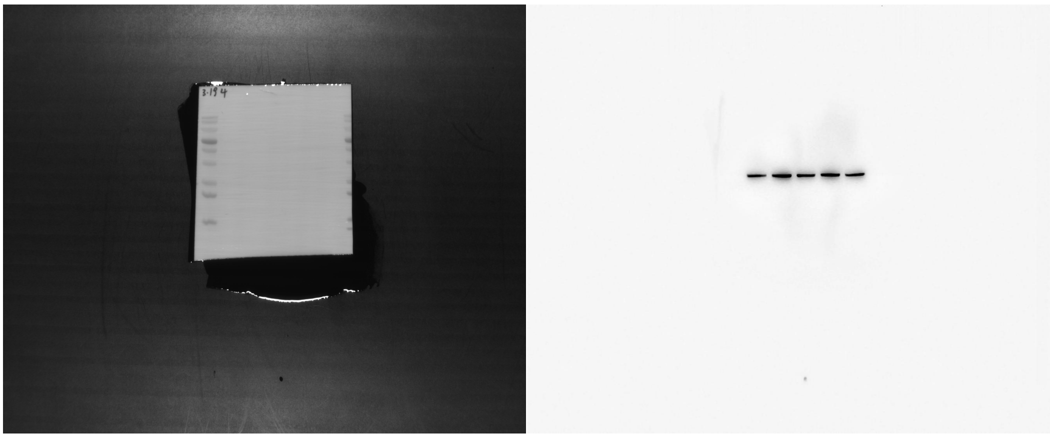

Figure 7E

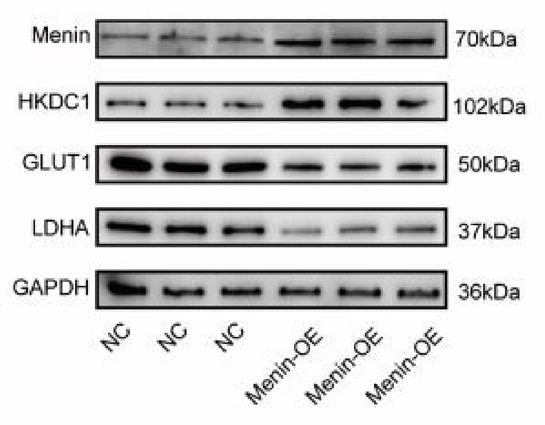

Menin

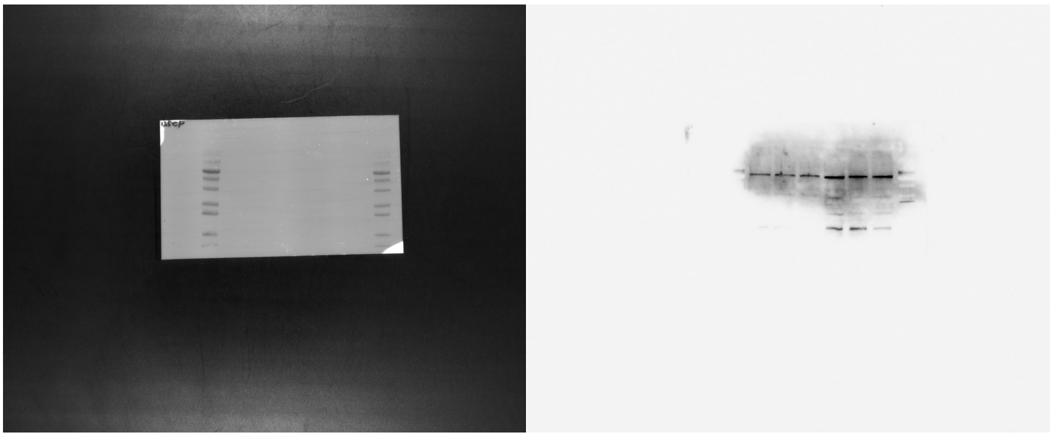

HKDC1

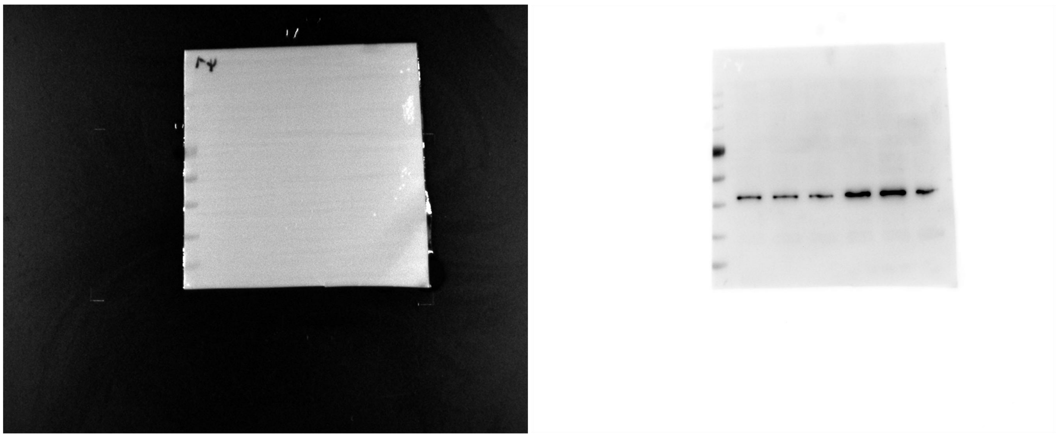

GLUT1

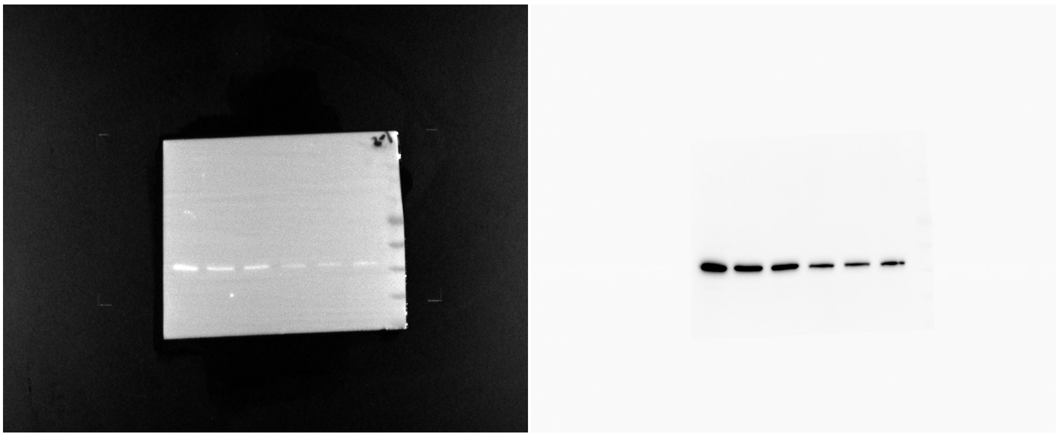

LDHA

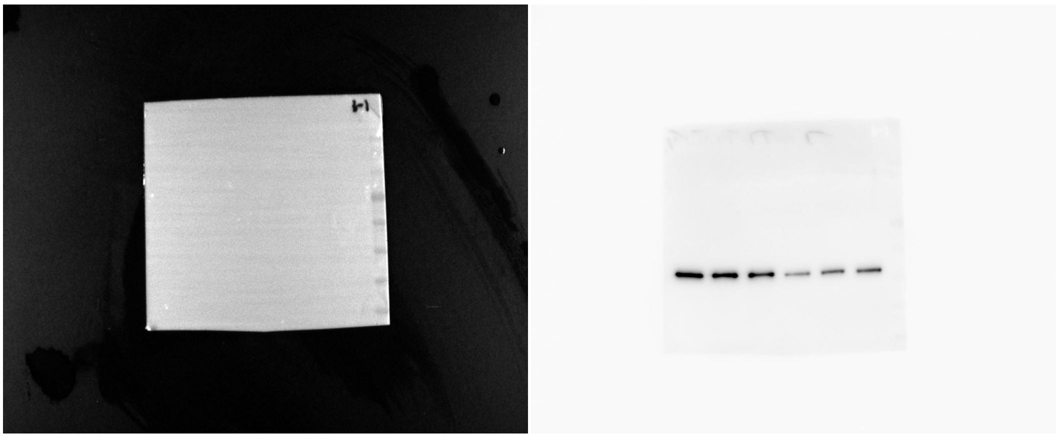

GAPDH

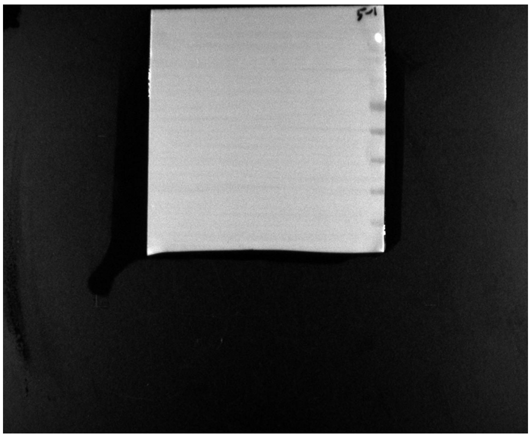

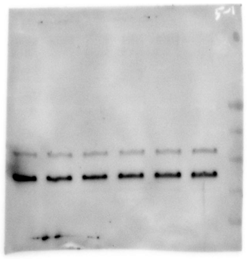

Figure S3

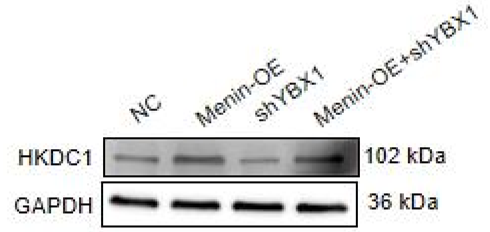

HKDC1

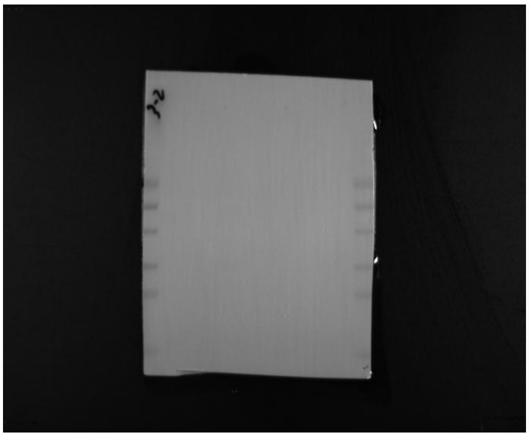

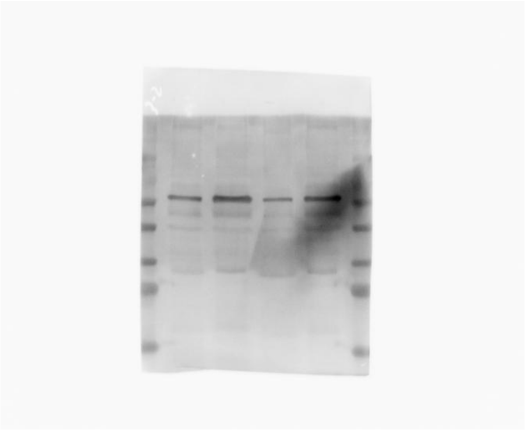

GAPDH

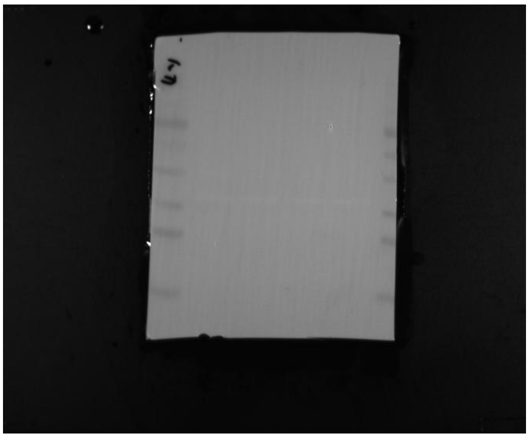

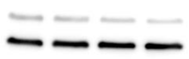

Supplement: Data S1. Raw WB Figures 5, 3F, 7E, and S3 [file mmc2.pdf]
