# Supplementary material for: Menin regulates YBX1 nucleus translocation to boost the HKDC1 transcription and affects pancreatic cancer glycolysis
Source: iScience. 2025 Aug 7;28(9):113245. doi: 10.1016/j.isci.2025.113245 (PMC12396302; doi:10.1016/j.isci.2025.113245)
Supplement: File S1. Western Blot Data [file mmc3.zip › Western Blot Data/README.docx]

**FIG 1C – Western blot lane assignment**

| **Cell line** | **Lane 1** | **Lane 2** |
| --- | --- | --- |
| **PL45** | NC – empty-vector negative control | Menin-OE – Menin over-expression |
| **BxPC-3** | NC – empty-vector negative control | Menin-OE – Menin over-expression |

Probed proteins: GLUT1 (~50 kDa), LDHA (~37 kDa), GAPDH (~36 kDa, loading control).

**FIG 3D-Western blot lane assignment**

| **Cell line** | **Lane 1** | **Lane 2** |
| --- | --- | --- |
| **PL45** | NC – empty-vector negative control | Menin-OE – Menin over-expression |
| **BxPC-3** | NC – empty-vector negative control | Menin-OE – Menin over-expression |

Probed proteins: HKDC1 (~102 kDa) and GAPDH (~36 kDa, loading control).

**FIG 3F – Western blot lane assignment**

| **Cell line** | **Lane 1** | **Lane 2** | **Lane 3** |
| --- | --- | --- | --- |
| **PL45** | shCtrl – non-targeting shRNA control | shHKDC1-1 – HKDC1 shRNA knockdown clone #1 | shHKDC1-2 – HKDC1 shRNA knockdown clone #2 |
| **BxPC-3** | shCtrl – non-targeting shRNA control | shHKDC1-1 – HKDC1 shRNA knockdown clone #1 | shHKDC1-2 – HKDC1 shRNA knockdown clone #2 |

Probed proteins: GLUT1 (~50 kDa), LDHA (~37 kDa), GAPDH (~36 kDa, loading control).

**FIG 4A-Western blot lane assignment**

| **Cell line** | **Lane 1** | **Lane 2** | **Lane 3** | **Lane 4** |
| --- | --- | --- | --- | --- |
| **PL45** | **NC** – empty-vector negative control | **Menin-OE** – Menin over-expression | **shHKDC1** – HKDC1 shRNA knock-down | **Menin-OE + shHKDC1** – combined Menin over-expression and HKDC1 knock-down |
| **BxPC-3** | **NC** – empty-vector negative control | **Menin-OE** – Menin over-expression | **shHKDC1** – HKDC1 shRNA knock-down | **Menin-OE + shHKDC1** – combined Menin over-expression and HKDC1 knock-down |

Probed proteins: GLUT1 (~50 kDa), LDHA (~37 kDa), GAPDH (~36 kDa, loading control).

**FIG 5D-Co-immunoprecipitation Western blot lane assignment**

**Probed proteins: Menin (~70 kDa) and YBX1 (~36 kDa)**

(i) Menin IP

| **Cell line** | **Lane 1** | **Lane 2** | **Lane 3** |
| --- | --- | --- | --- |
| **PL45** | Input – whole-cell lysate | IgG IP – nonspecific antibody control | Menin IP – immunoprecipitation with anti-Menin antibody |
| **BxPC-3** | Input – whole-cell lysate | IgG IP – nonspecific antibody control | Menin IP – immunoprecipitation with anti-Menin antibody |

(ii) YBX1 IP

| **Cell line** | **Lane 1** | **Lane 2** | **Lane 3** |
| --- | --- | --- | --- |
| **PL45** | Input – whole-cell lysate | IgG IP – nonspecific antibody control | YBX1 IP – immunoprecipitation with anti-YBX1 antibody |
| **BxPC-3** | Input – whole-cell lysate | IgG IP – nonspecific antibody control | YBX1 IP – immunoprecipitation with anti-YBX1 antibody |

**FIG 5F– GST pull-down lane assignment**

| **Lane** | **GST** | **GST-Menin** | **HIS-YBX1** | **Experimental condition** |
| --- | --- | --- | --- | --- |
| **1** | + | – | + | Control pull-down (GST alone incubated with HIS-YBX1) |
| **2** | – | + | + | Test pull-down (GST-Menin fusion incubated with HIS-YBX1) |

Probed bands:

**IB: HIS** – HIS-tagged YBX1 (~42 kDa) detected in the pull-down and input fractions.

**IB: GST** – GST or GST-Menin fusion (~68 kDa) verified in the input fraction.

**GST pull-down lane assignment**

| **Lane** | **GST** | **GST-Menin-FL** | **GST-Menin (1-210 aa)** | **GST-Menin (211-450 aa)** | **GST-Menin (451-610 aa)** | **HIS-YBX1** | **Experimental condition** |
| --- | --- | --- | --- | --- | --- | --- | --- |
| **1** | + | – | – | – | – | + | GST alone incubated with HIS-YBX1 (negative control) |
| **2** | – | + | – | – | – | + | Full-length GST-Menin incubated with HIS-YBX1 |
| **3** | – | – | + | – | – | + | GST-Menin N-terminal fragment (aa 1-210) + HIS-YBX1 |
| **4** | – | – | – | + | – | + | GST-Menin middle fragment (aa 211-450) + HIS-YBX1 |
| **5** | – | – | – | – | + | + | GST-Menin C-terminal fragment (aa 451-610) + HIS-YBX1 |

Blotting:

**IB: HIS** detects bound HIS-tagged YBX1 (~42 kDa) in the pull-down and input fractions.

**IB: GST** verifies the presence of GST or GST-Menin bait proteins (~68 kDa) in the input fraction.

**FIG 6A Subcellular-fraction Western blot lane assignment**

| **Fraction** | **Lane 1** | **Lane 2** |
| --- | --- | --- |
| **Cytoplasm** | **NC** – empty-vector negative control | **Menin-OE** – Menin over-expression |
| **Nucleus** | **NC** – empty-vector negative control | **Menin-OE** – Menin over-expression |

Probed proteins:

**YBX1** (~42 kDa) in both cytoplasmic and nuclear fractions

**GAPDH** (~36 kDa) as the cytoplasmic loading control

**Histone H3** (~15 kDa) as the nuclear loading control

**FIG 6C-Cycloheximide-chase Western blot lane assignment**

| **Cell line** | **Condition** | **Lane 1** | **Lane 2** | **Lane 3** | **Lane 4** |
| --- | --- | --- | --- | --- | --- |
| **PL45** | **NC** (empty-vector control) | 0 h CHX | 2 h CHX | 4 h CHX | 8 h CHX |
|  | **Menin-OE** (Menin over-expression) | 0 h CHX | 2 h CHX | 4 h CHX | 8 h CHX |
| **BxPC-3** | **NC** (empty-vector control) | 0 h CHX | 2 h CHX | 4 h CHX | 8 h CHX |
|  | **Menin-OE** (Menin over-expression) | 0 h CHX | 2 h CHX | 4 h CHX | 8 h CHX |

Experimental context: Cells were treated with cycloheximide (CHX) to block new protein synthesis, and YBX1 degradation was monitored over the indicated time points (0 h, 2 h, 4 h, 8 h).

Probed proteins:

**YBX1** (~36 kDa) – target protein

**Lamin B1** (~68 kDa) – nuclear loading control

**FIG 6D-Flag immunoprecipitation (IP) Western blot lane assignment**

| **Fraction** | **Lane 1** | **Lane 2** |
| --- | --- | --- |
| **flag-IP** | **NC** – empty-vector negative control (no Flag-Menin) | **Menin-OE** – Flag-Menin over-expression, immunoprecipitated with anti-Flag |
| **Input** | **NC** – whole-cell lysate, empty-vector control | **Menin-OE** – whole-cell lysate, Menin over-expression |

Probed proteins:

**YBX1** (~36 kDa) – co-precipitating partner / target protein

**Menin** (~68 kDa) – bait protein (detected in IP and input)

**Lamin B1** (~68 kDa) – nuclear loading control (input fraction only)

**FIG 7E-Western blot lane assignment (Menin-OE animal model, three biological replicates)**

| **Lane** | **Group** | **Biological replicate** | **Description** |
| --- | --- | --- | --- |
| **1** | NC (empty-vector control) | Mouse #1 | Control animal sample |
| **2** | NC (empty-vector control) | Mouse #2 | Control animal sample |
| **3** | NC (empty-vector control) | Mouse #3 | Control animal sample |
| **4** | Menin-OE (Menin over-expression) | Mouse #1 | Menin-OE animal sample |
| **5** | Menin-OE (Menin over-expression) | Mouse #2 | Menin-OE animal sample |
| **6** | Menin-OE (Menin over-expression) | Mouse #3 | Menin-OE animal sample |

Probed proteins: Menin (~70 kDa), HKDC1 (~102 kDa), GLUT1 (~50 kDa), LDHA (~37 kDa), GAPDH (~36 kDa, loading control).

**FIG S1A-Western blot lane assignment (PL45 cells)**

| **Cell line** | **Lane 1** | **Lane 2** |
| --- | --- | --- |
| **PL45** | **NC** – empty-vector negative control | **Menin-OE** – Menin over-expression |

Probed proteins: Menin (~70 kDa) and GAPDH (~36 kDa, loading control).

**FIG S1B-Western blot lane assignment (**BxPC-3** cells)**

| **Cell line** | **Lane 1** | **Lane 2** |
| --- | --- | --- |
| ****BxPC-3**** | **NC** – empty-vector negative control | **Menin-OE** – Menin over-expression |

Probed proteins: Menin (~70 kDa) and GAPDH (~36 kDa, loading control).

**FIG S2C-Western blot lane assignment**

| **Cell line** | **Lane 1** | **Lane 2** | **Lane 3** |
| --- | --- | --- | --- |
| **PL45** | **shCtrl** – non-targeting shRNA control | **shHKDC1-1** – HKDC1 shRNA knock-down clone #1 | **shHKDC1-2** – HKDC1 shRNA knock-down clone #2 |
| **BxPC-3** | **shCtrl** – non-targeting shRNA control | **shHKDC1-1** – HKDC1 shRNA knock-down clone #1 | **shHKDC1-2** – HKDC1 shRNA knock-down clone #2 |

Probed proteins: HKDC1 (~102 kDa) and GAPDH (~36 kDa, loading control).

**FIG S3-Western blot lane assignment**

| **Lane** | **Experimental condition** | **Description** |
| --- | --- | --- |
| **1** | **NC** | Empty-vector negative control |
| **2** | **Menin-OE** | Menin over-expression |
| **3** | **shYBX1** | YBX1 shRNA knock-down |
| **4** | **Menin-OE + shYBX1** | Combined Menin over-expression and YBX1 knock-down |

Probed proteins: HKDC1 (~102 kDa) and GAPDH (~36 kDa, loading control).
