# Supplementary figures and images for: Menin regulates YBX1 nucleus translocation to boost the HKDC1 transcription and affects pancreatic cancer glycolysis
Source: iScience. 2025 Aug 7;28(9):113245. doi: 10.1016/j.isci.2025.113245 (PMC12396302; doi:10.1016/j.isci.2025.113245)

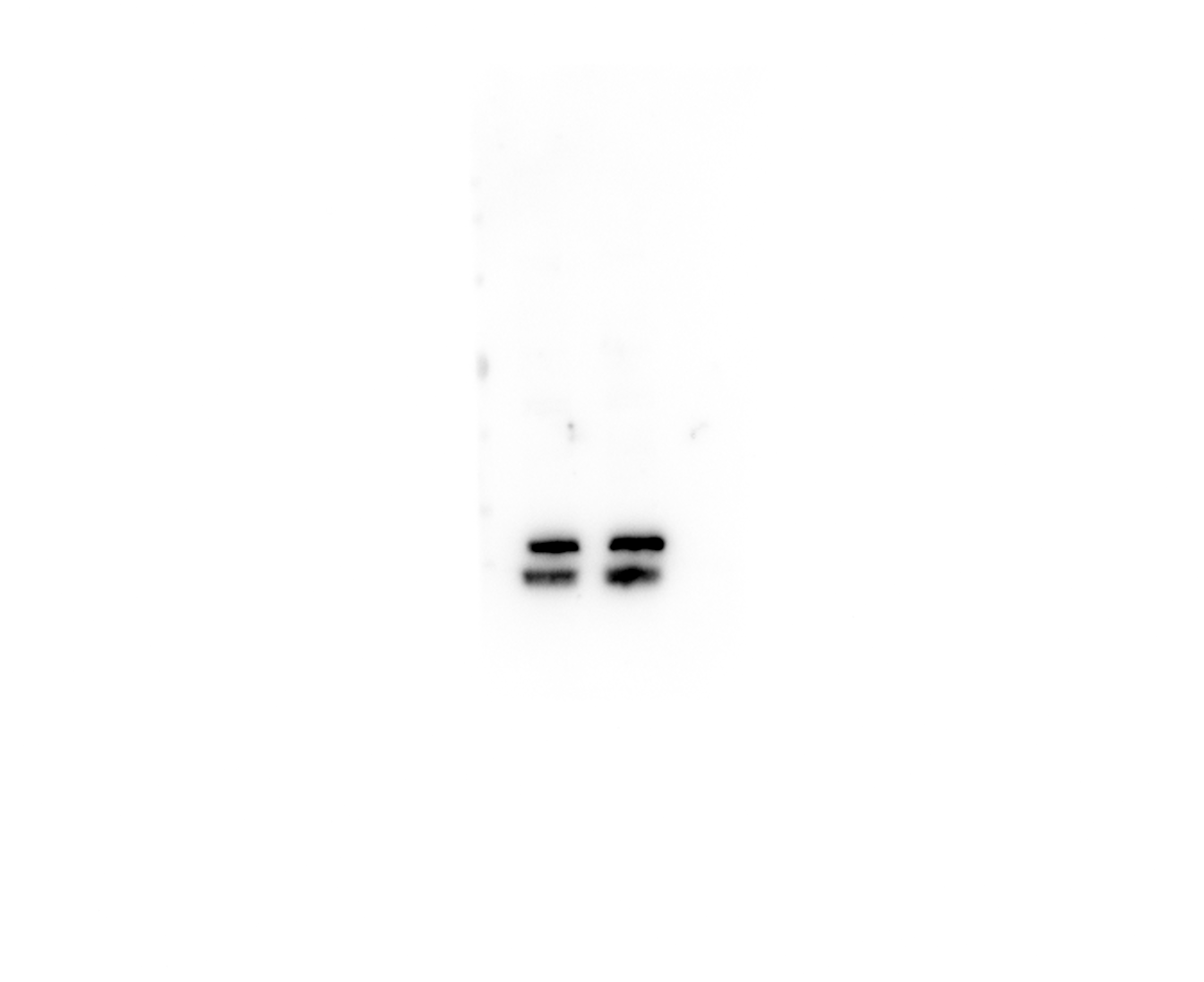

Supplement: File S1. Western Blot Data [file mmc3.zip › Western Blot Data/WB/Fig1C-Bx-PC-3-GAPDH.Tif]

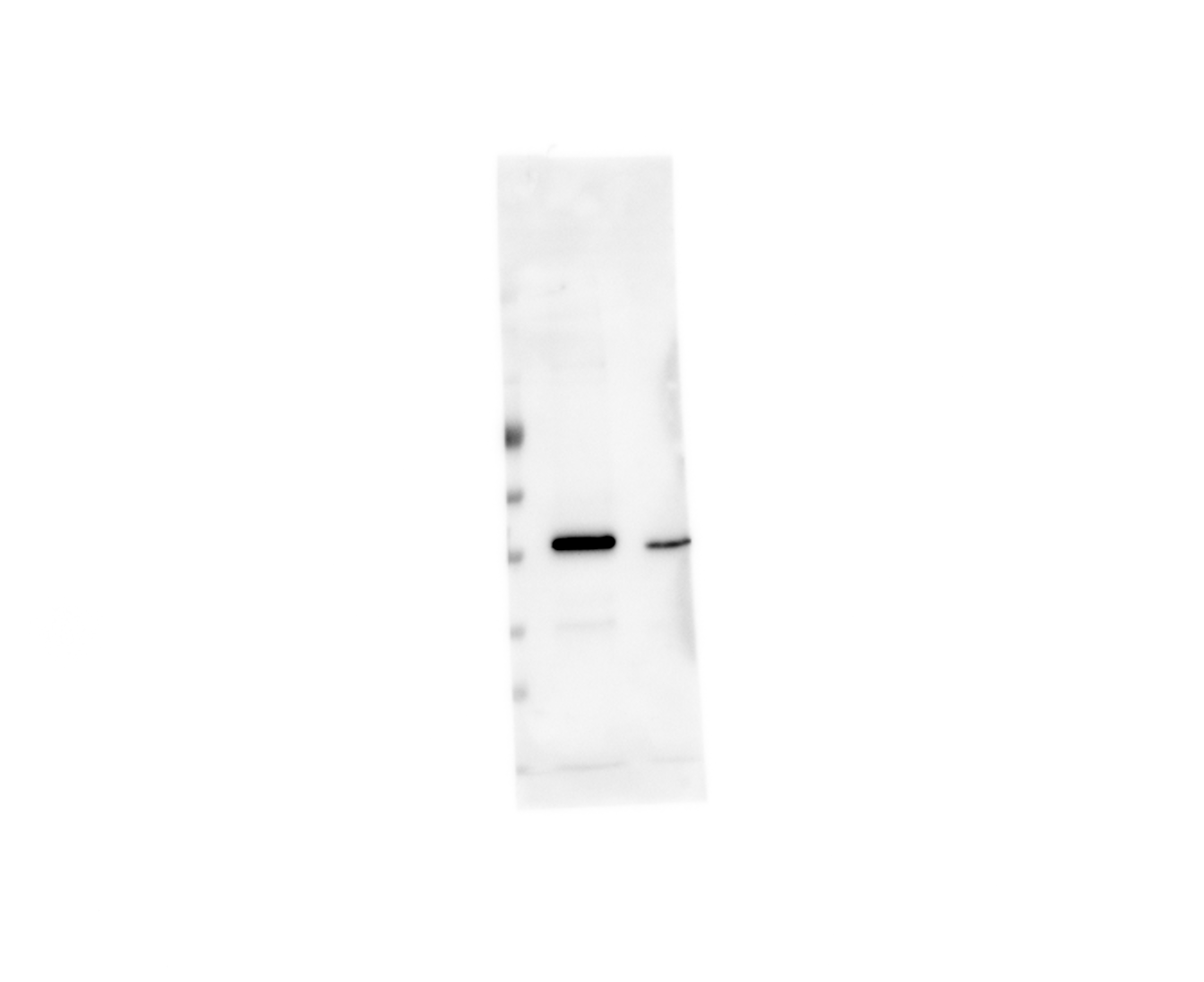

Supplement: File S1. Western Blot Data [file mmc3.zip › Western Blot Data/WB/Fig1C-Bx-PC-3-GLUT1.Tif]

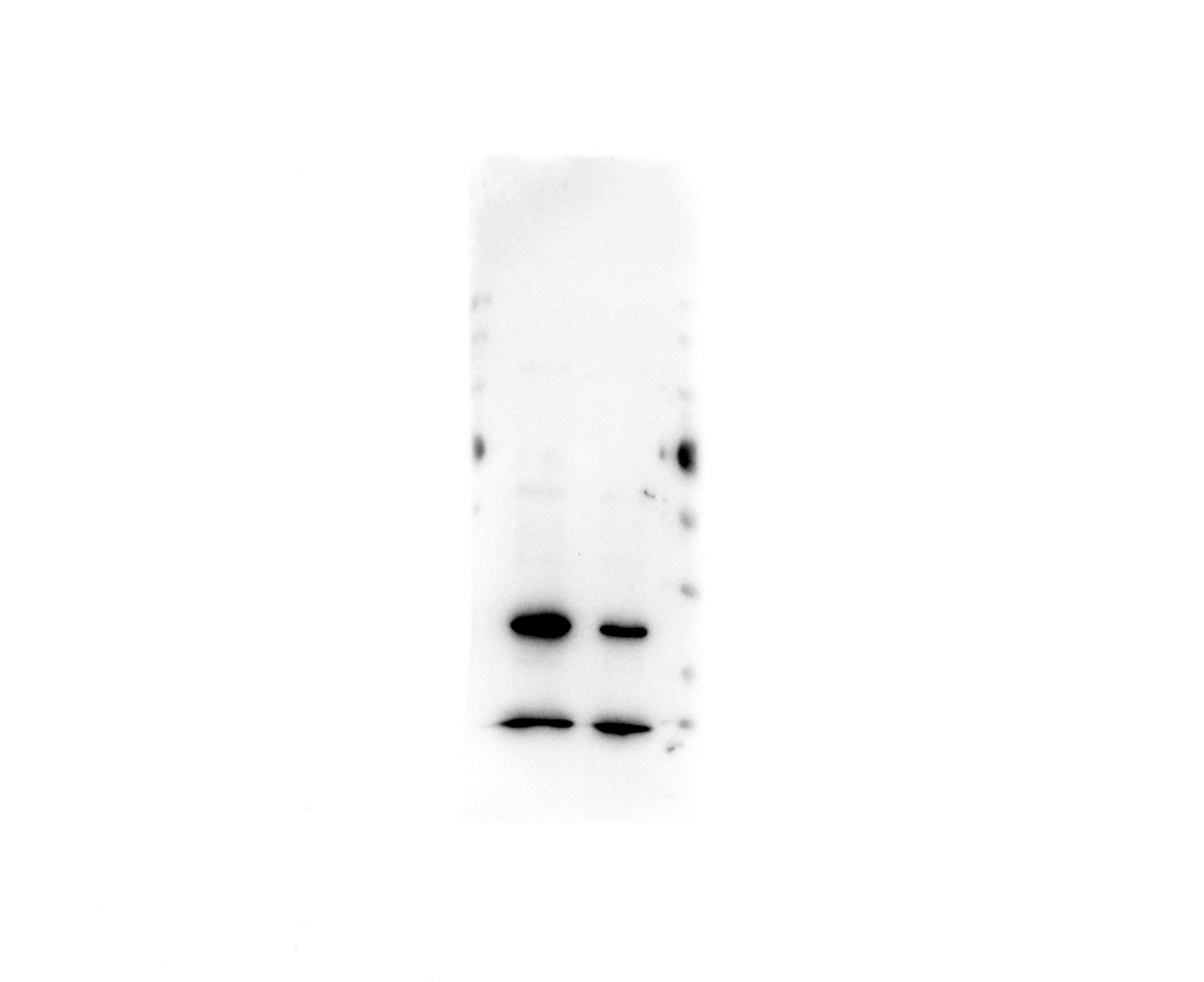

Supplement: File S1. Western Blot Data [file mmc3.zip › Western Blot Data/WB/Fig1C-Bx-PC-3-LDHA.Tif]

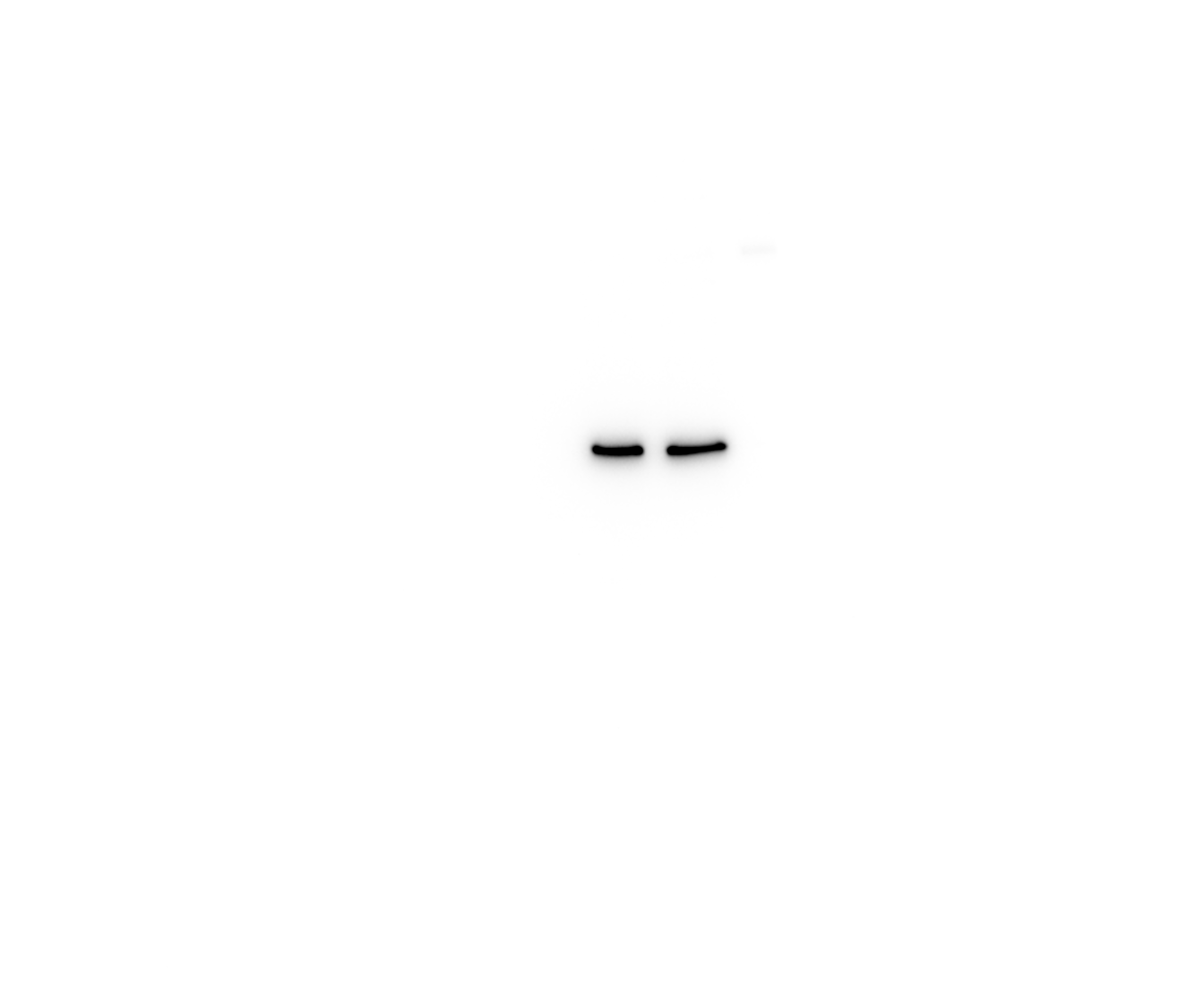

Supplement: File S1. Western Blot Data [file mmc3.zip › Western Blot Data/WB/Fig1C-PL45-GAPDH.Tif]

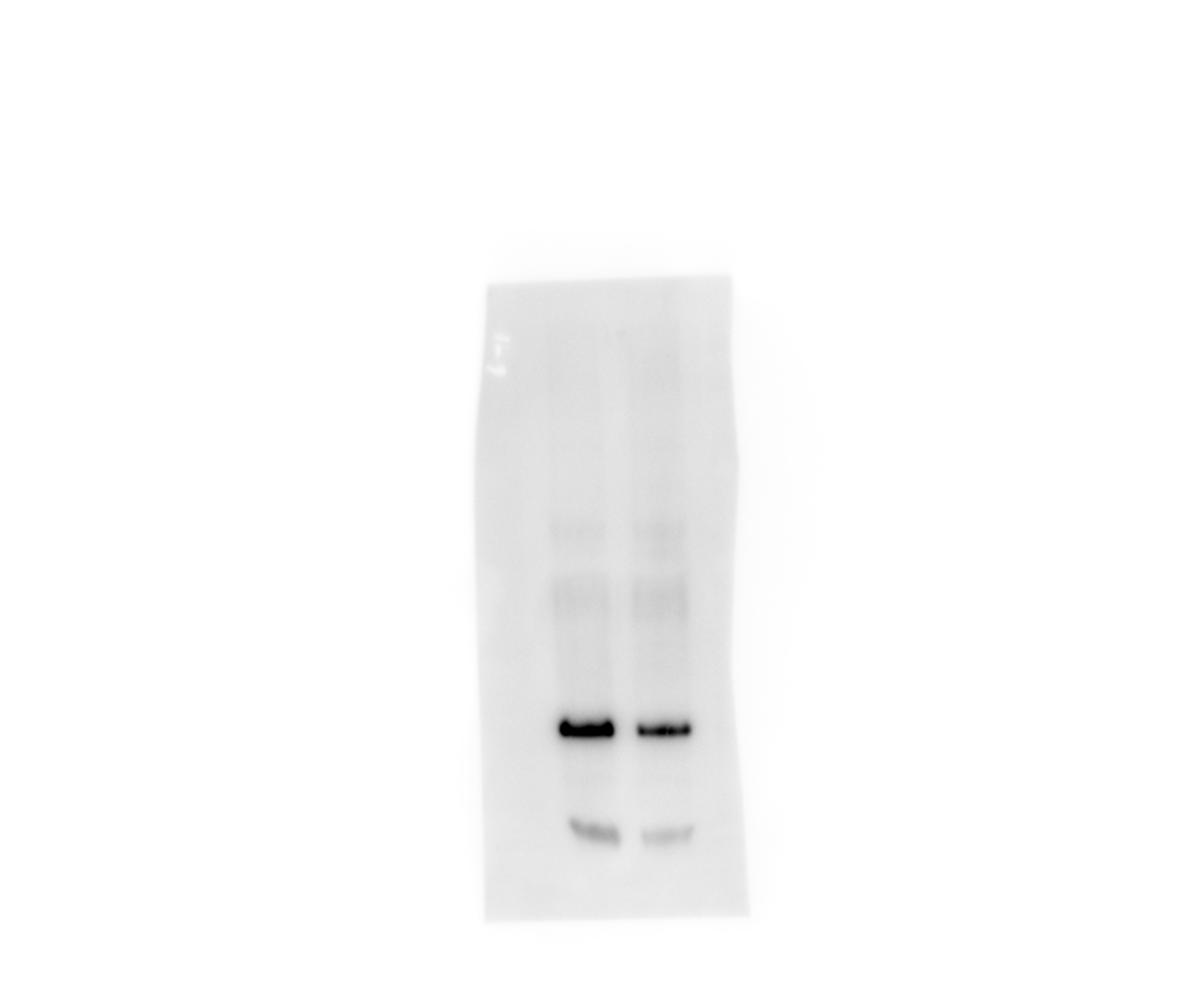

Supplement: File S1. Western Blot Data [file mmc3.zip › Western Blot Data/WB/Fig1C-PL45-GLUT1.Tif]

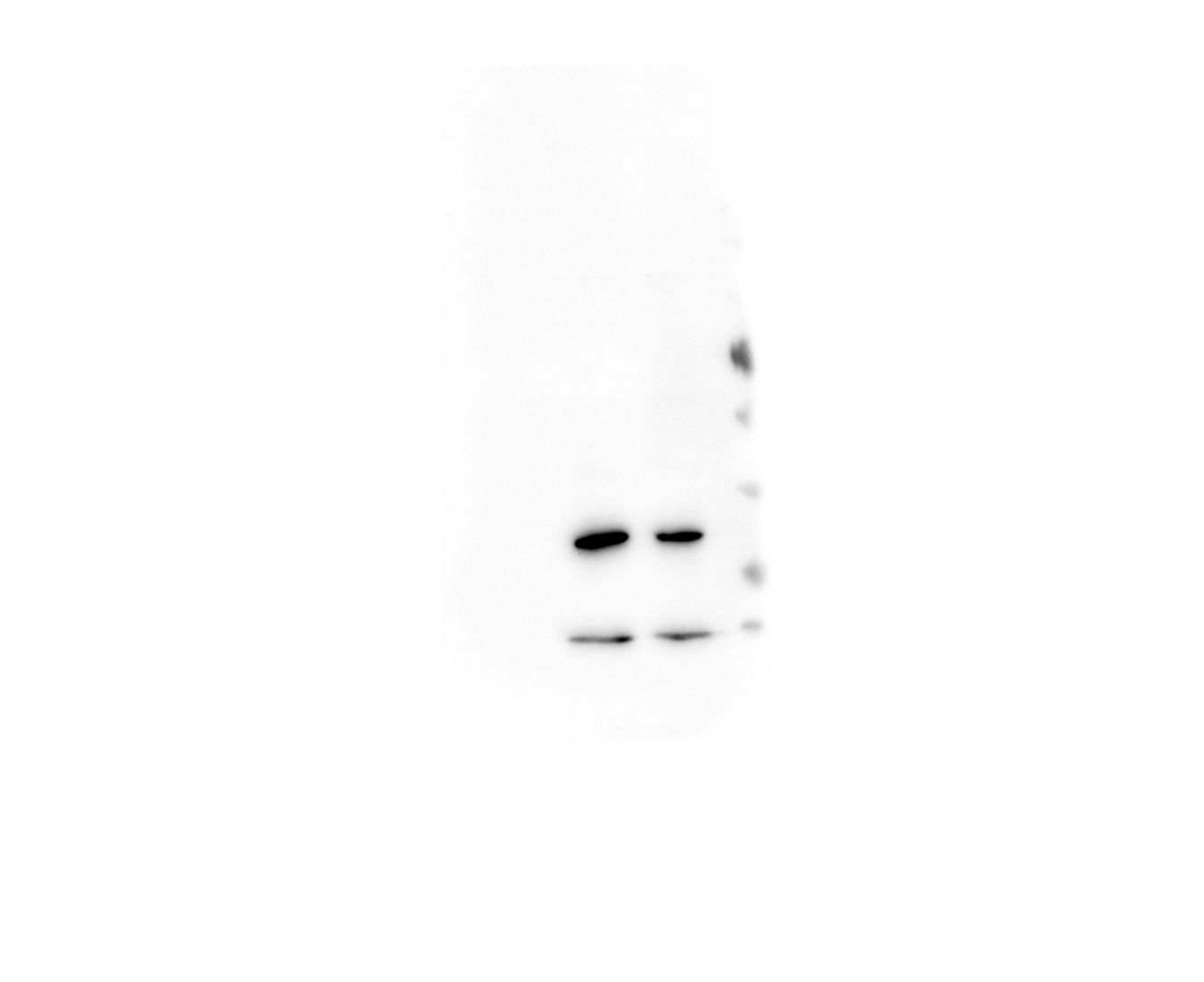

Supplement: File S1. Western Blot Data [file mmc3.zip › Western Blot Data/WB/Fig1C-PL45-LDHA.Tif]

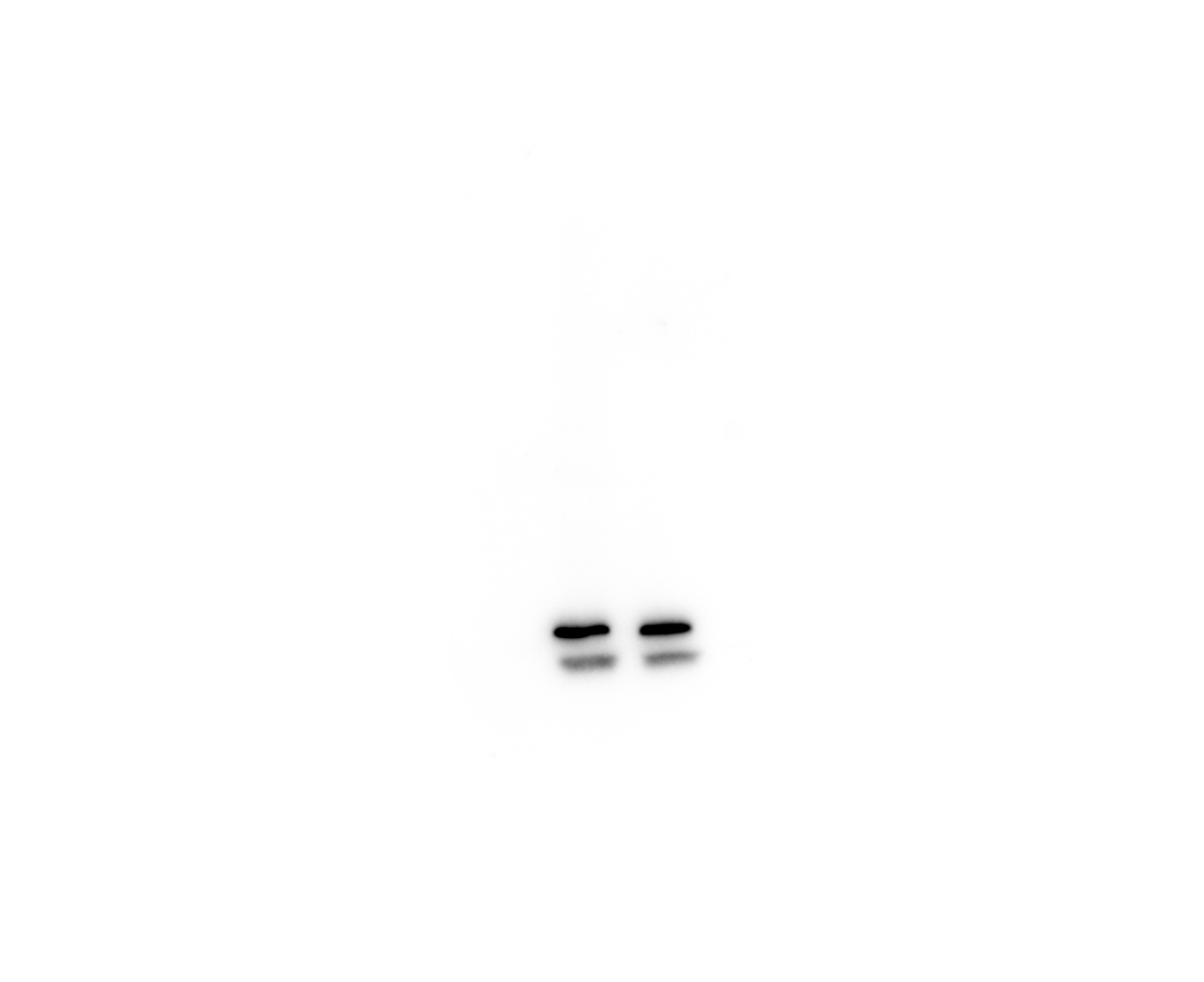

Supplement: File S1. Western Blot Data [file mmc3.zip › Western Blot Data/WB/Fig3D-Bx-PC-3-GAPDH.Tif]

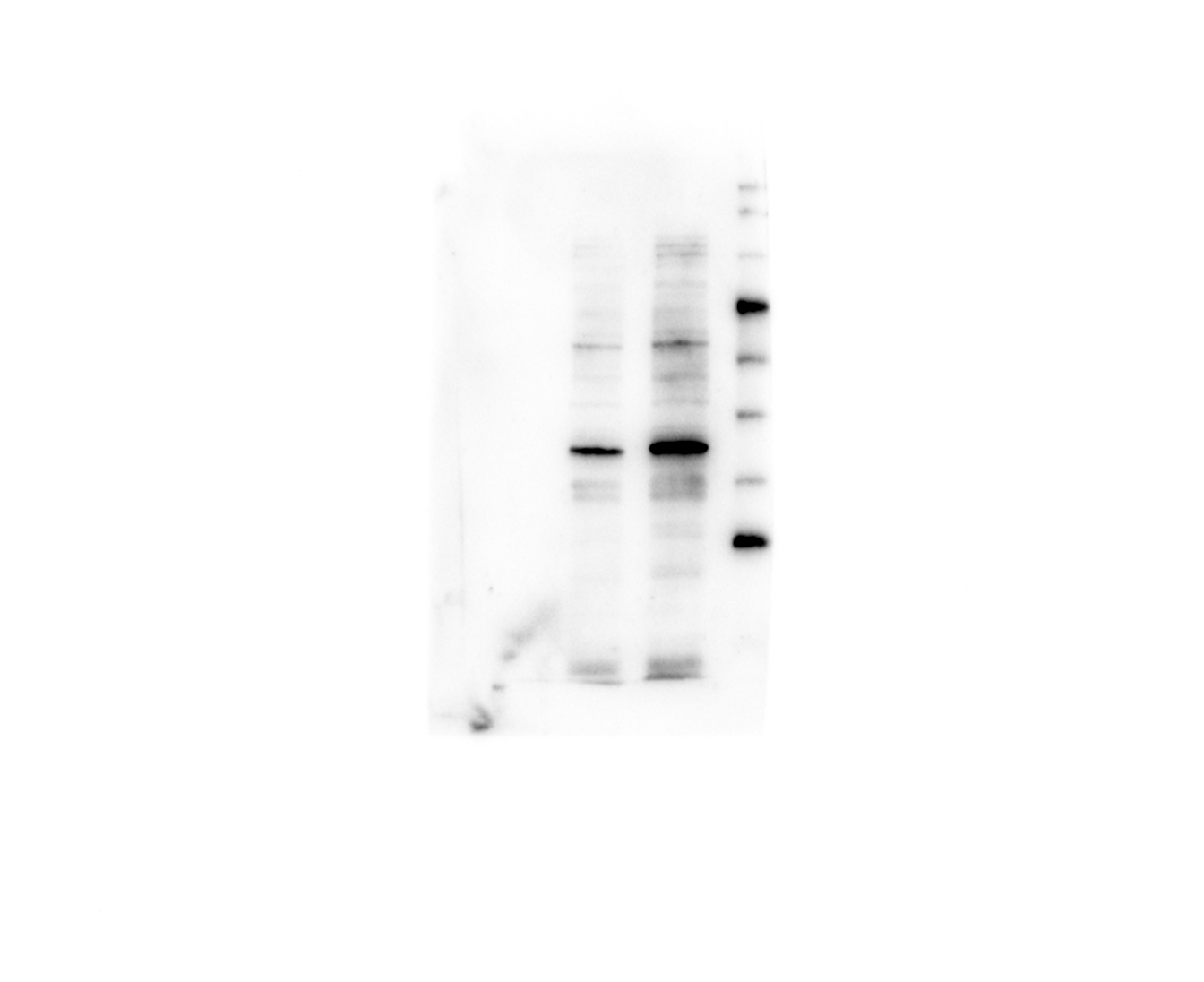

Supplement: File S1. Western Blot Data [file mmc3.zip › Western Blot Data/WB/Fig3D-Bx-PC-3-HKDC1.Tif]

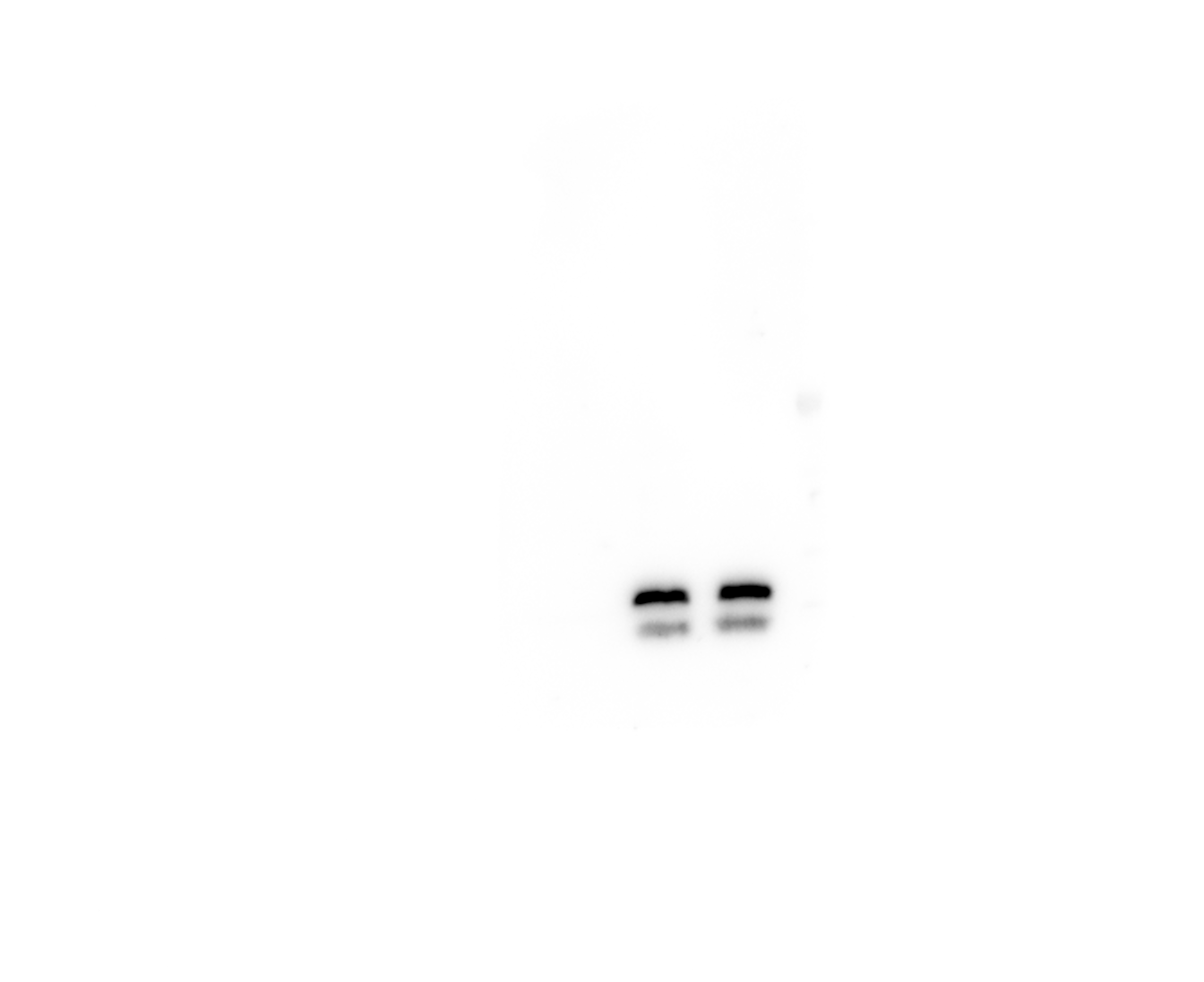

Supplement: File S1. Western Blot Data [file mmc3.zip › Western Blot Data/WB/Fig3D-PL45-GAPDH.Tif]

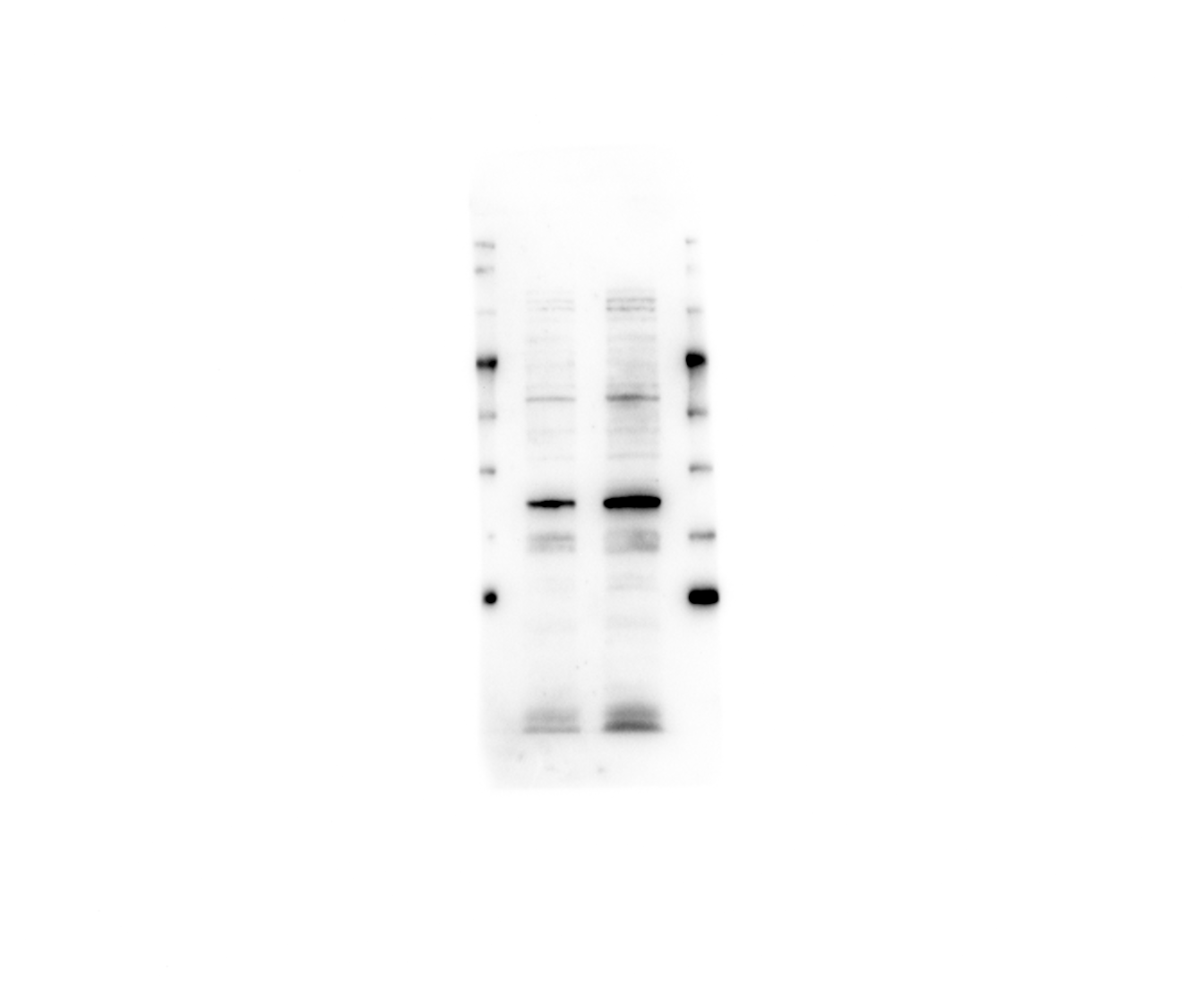

Supplement: File S1. Western Blot Data [file mmc3.zip › Western Blot Data/WB/Fig3D-PL45-HKDC1.Tif]

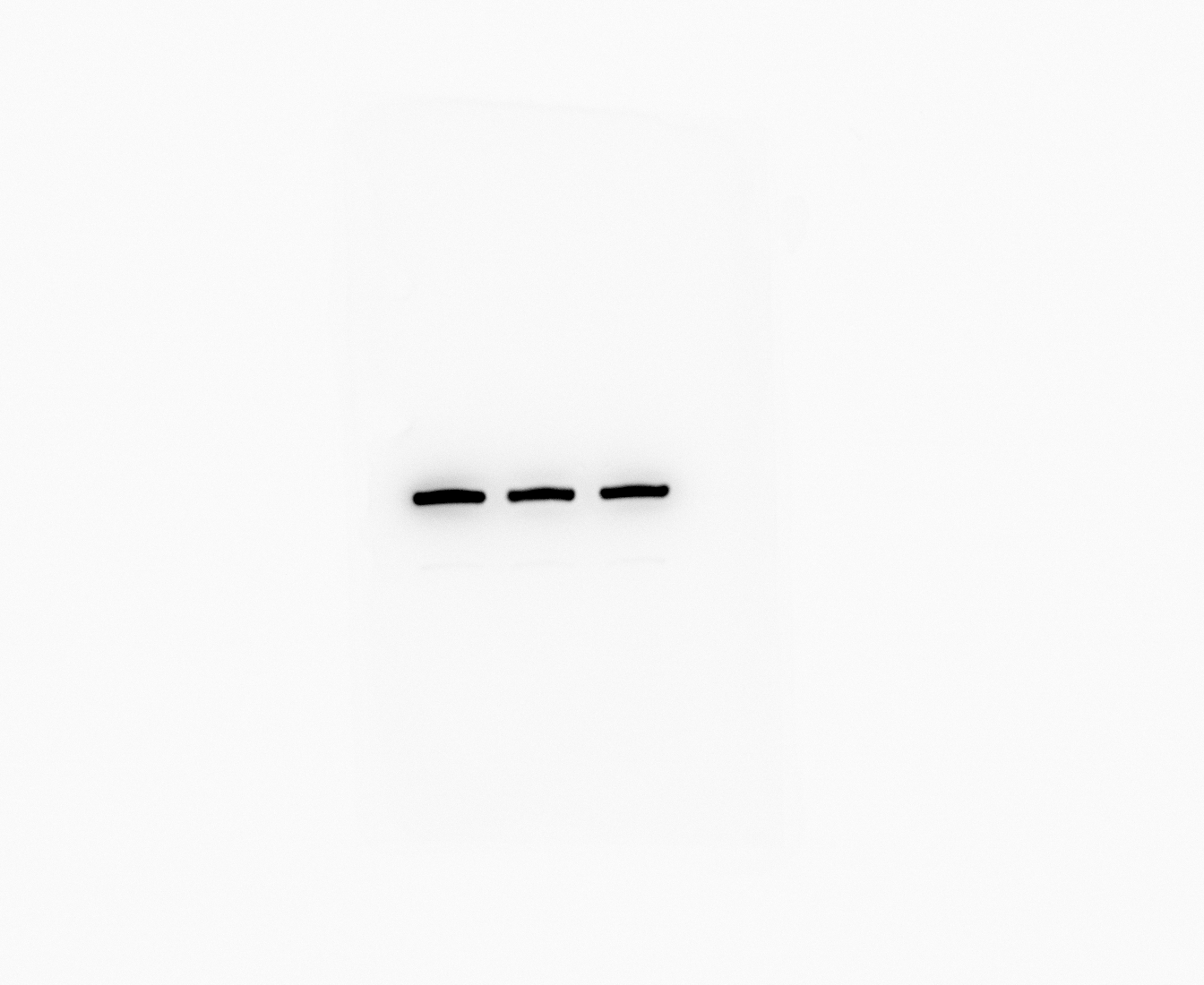

Supplement: File S1. Western Blot Data [file mmc3.zip › Western Blot Data/WB/Fig3F-Bx-PC-3-GAPDH.tif]

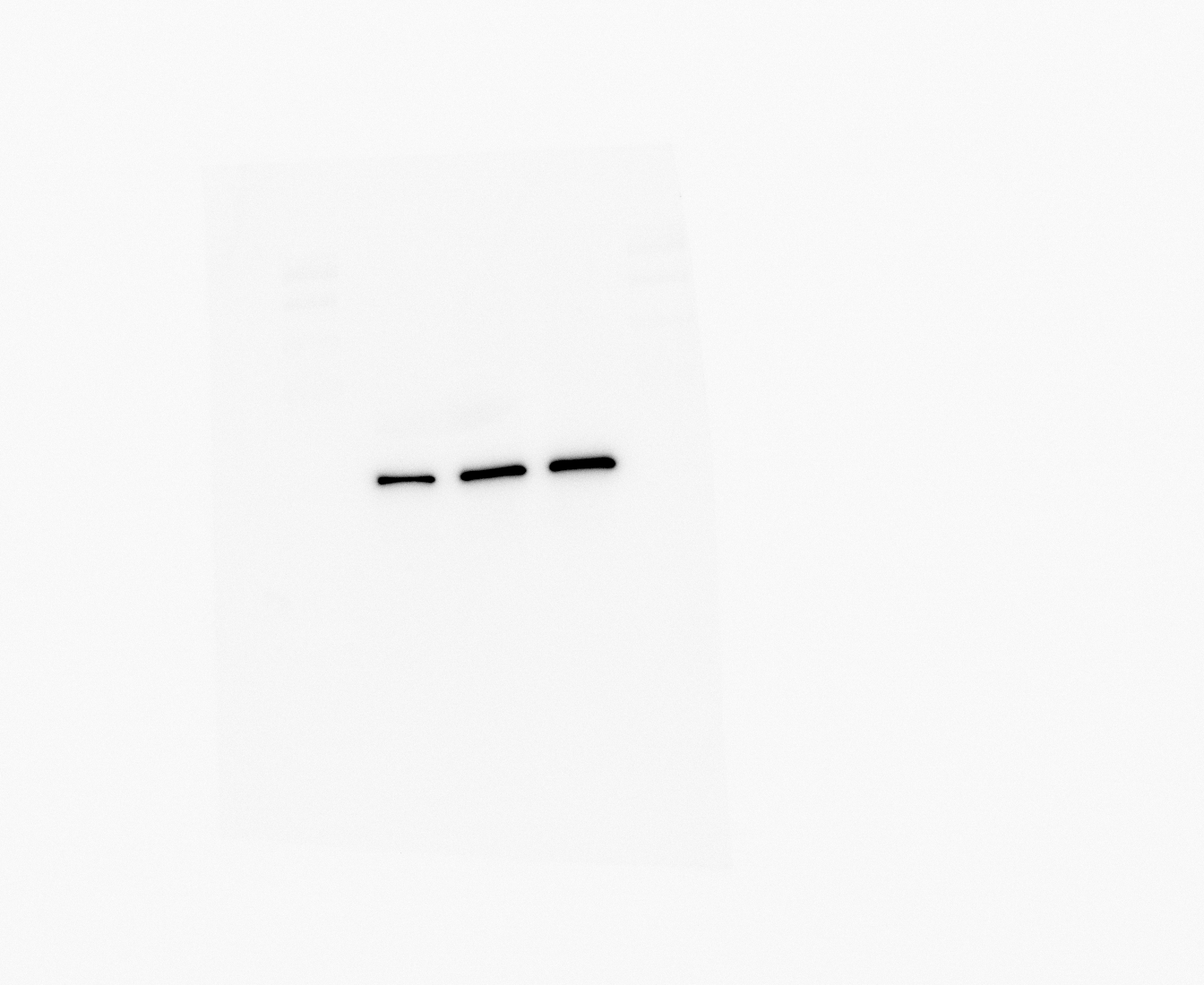

Supplement: File S1. Western Blot Data [file mmc3.zip › Western Blot Data/WB/Fig3F-Bx-PC-3-GLUT1.tif]

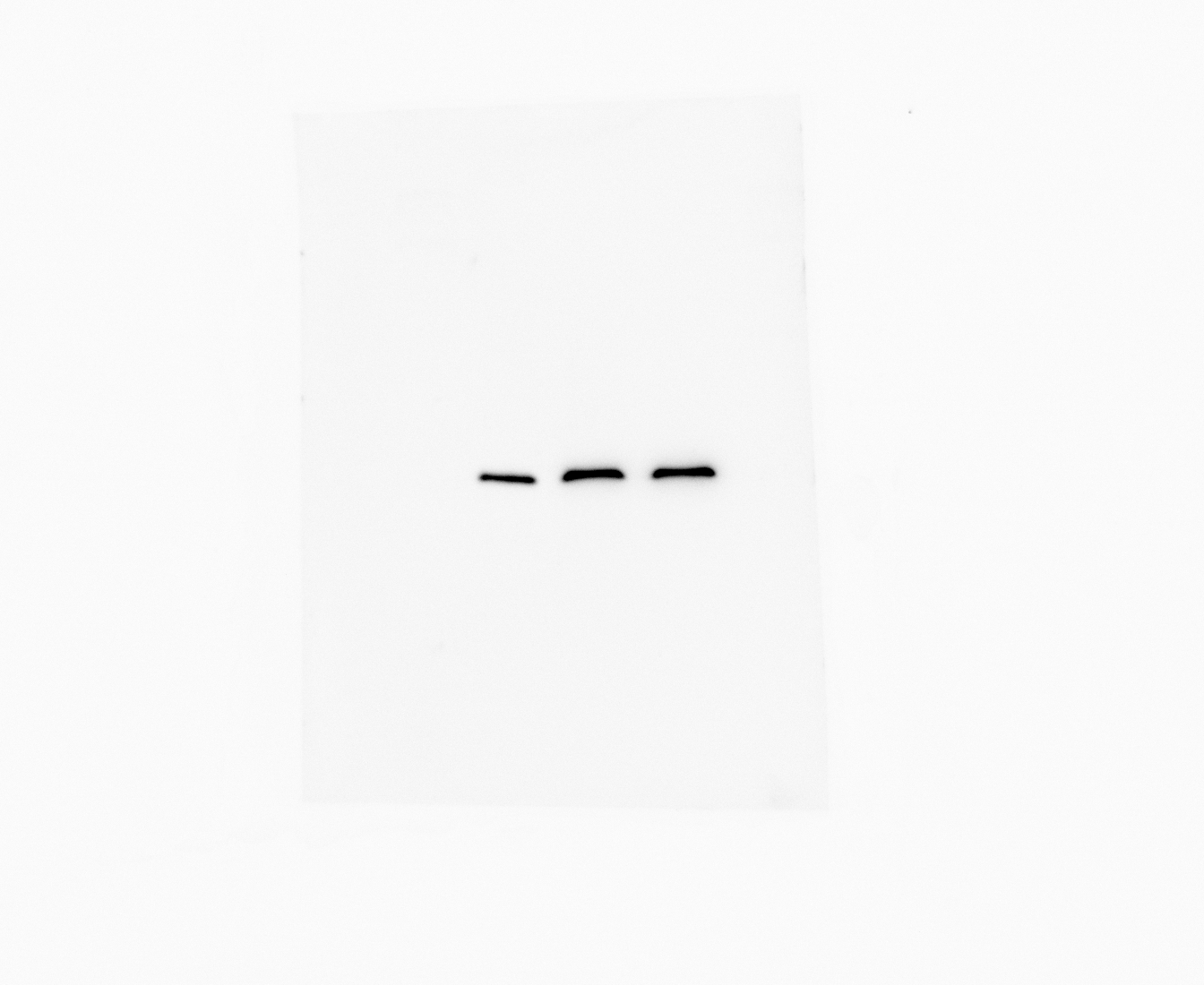

Supplement: File S1. Western Blot Data [file mmc3.zip › Western Blot Data/WB/Fig3F-Bx-PC-3-LDHA.tif]

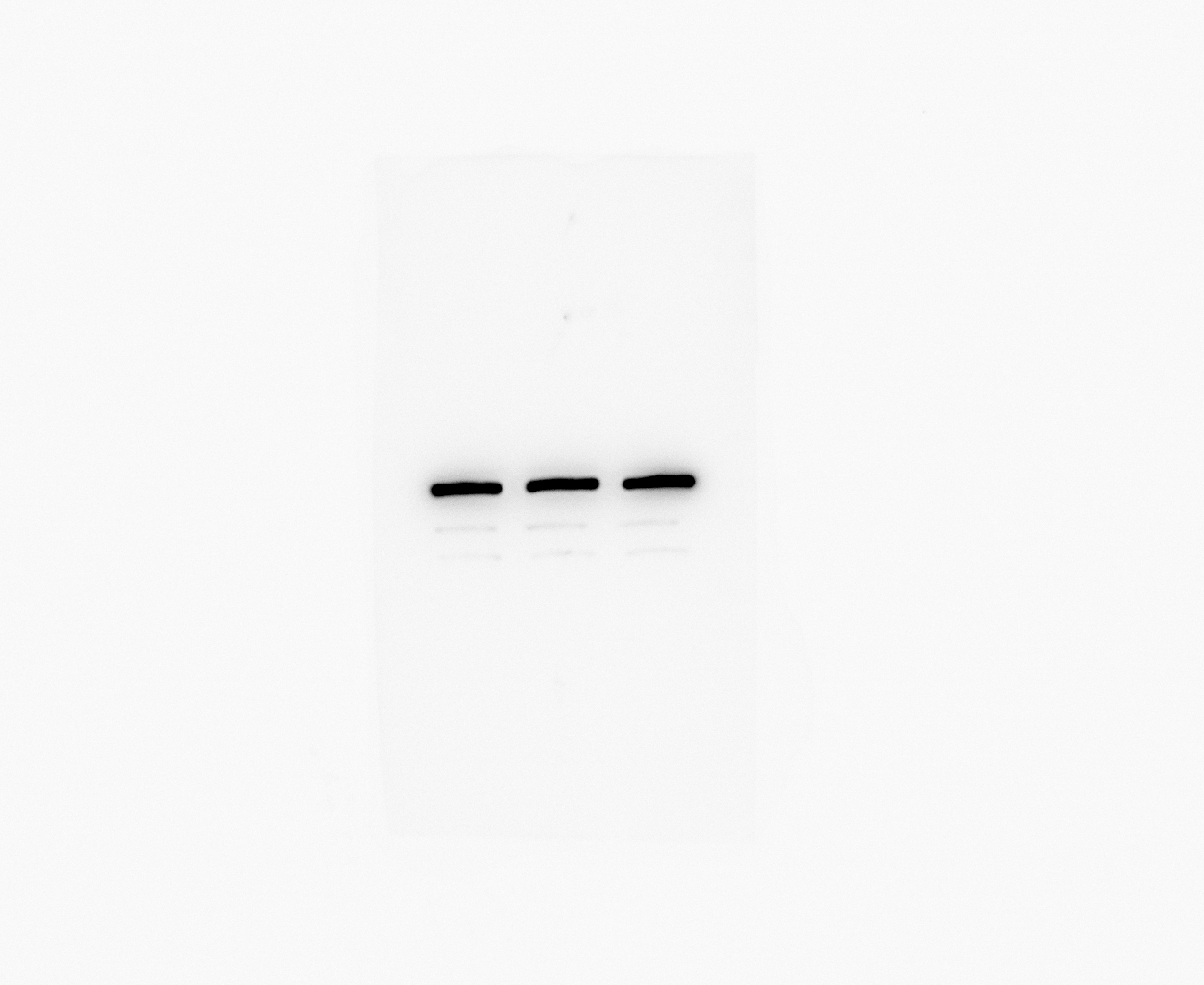

Supplement: File S1. Western Blot Data [file mmc3.zip › Western Blot Data/WB/Fig3F-PL45-GAPDH.tif]

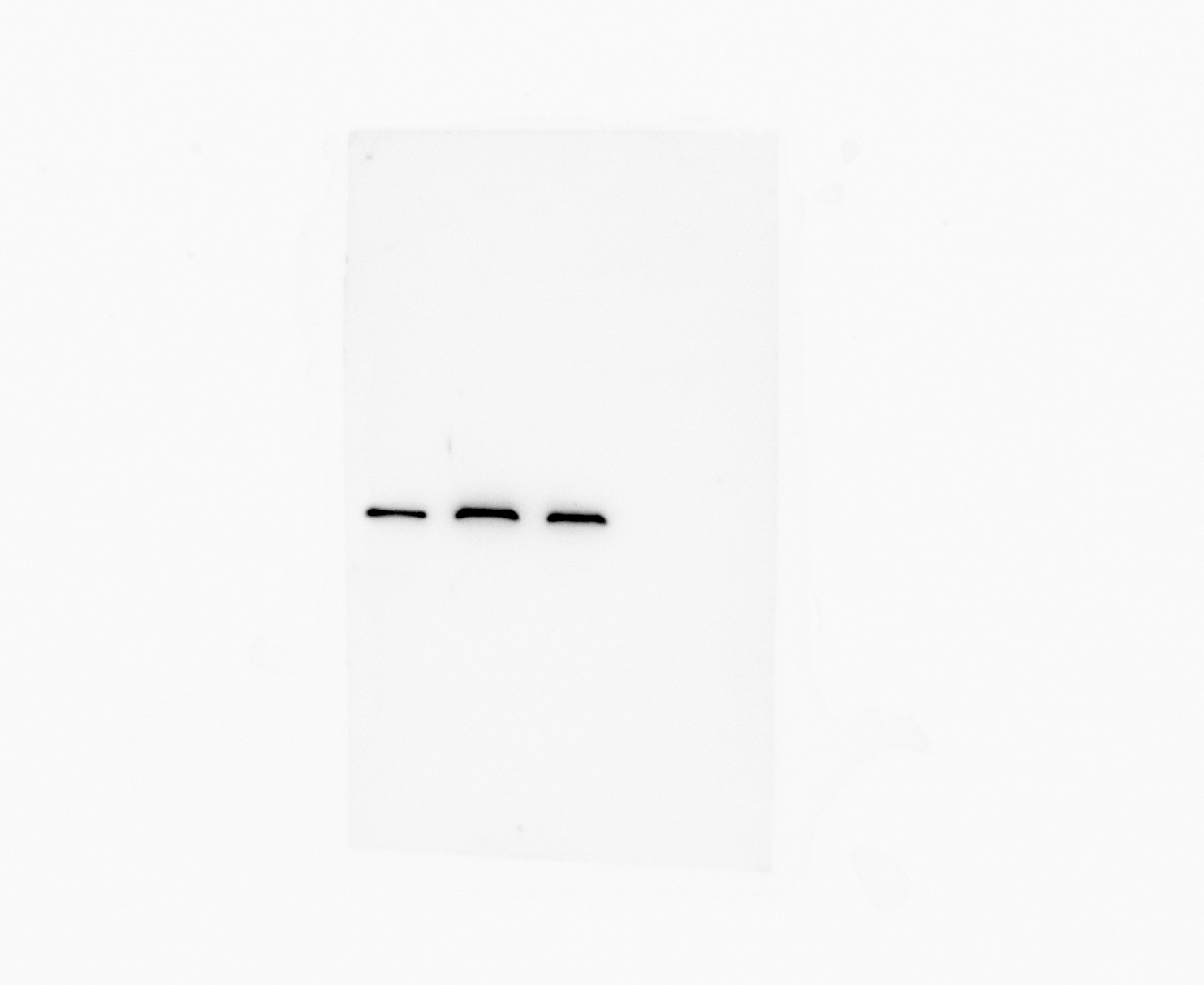

Supplement: File S1. Western Blot Data [file mmc3.zip › Western Blot Data/WB/Fig3F-PL45-LDHA.tif]

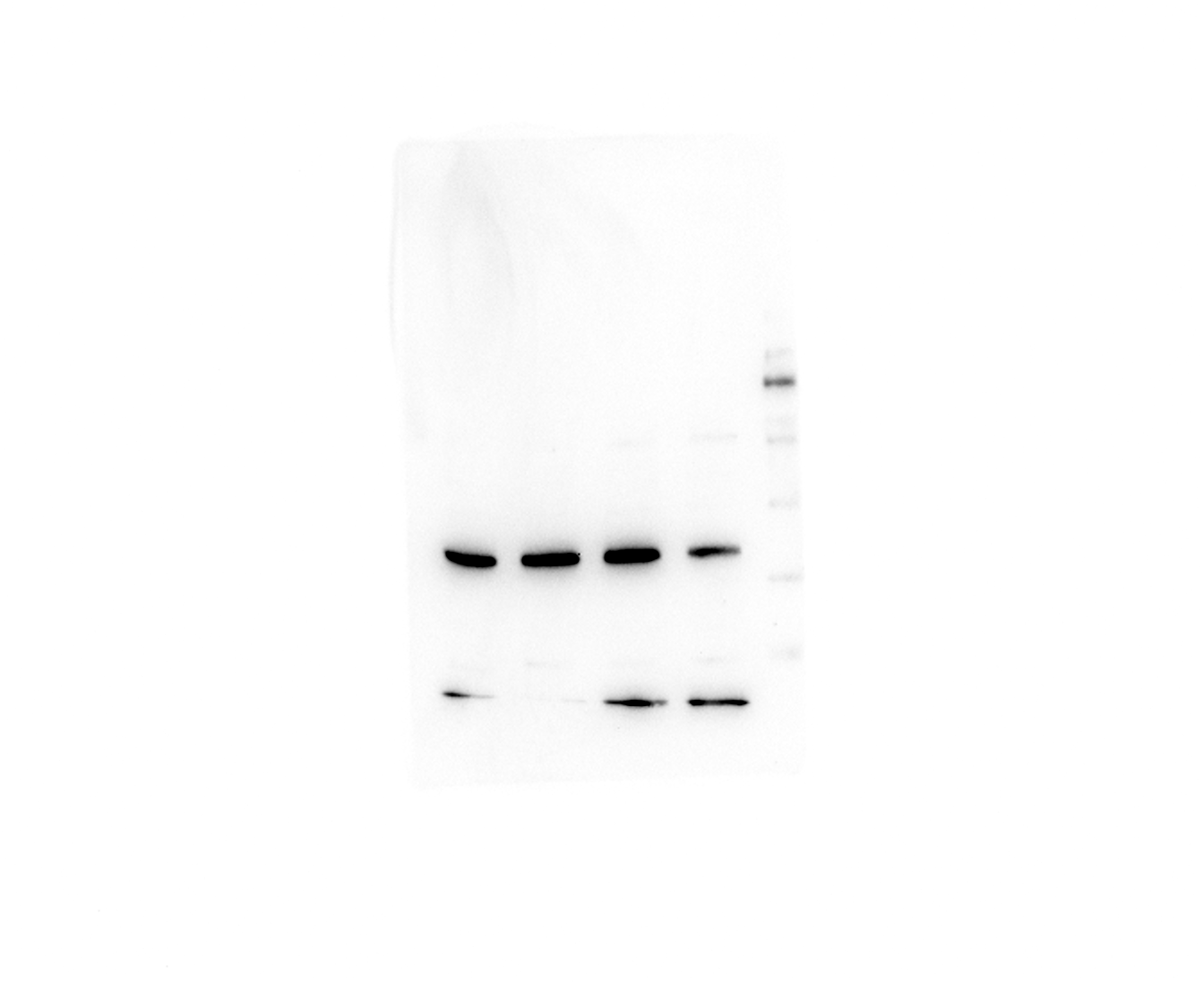

Supplement: File S1. Western Blot Data [file mmc3.zip › Western Blot Data/WB/Fig4A-Bx-PC-3-GAPDH.Tif]

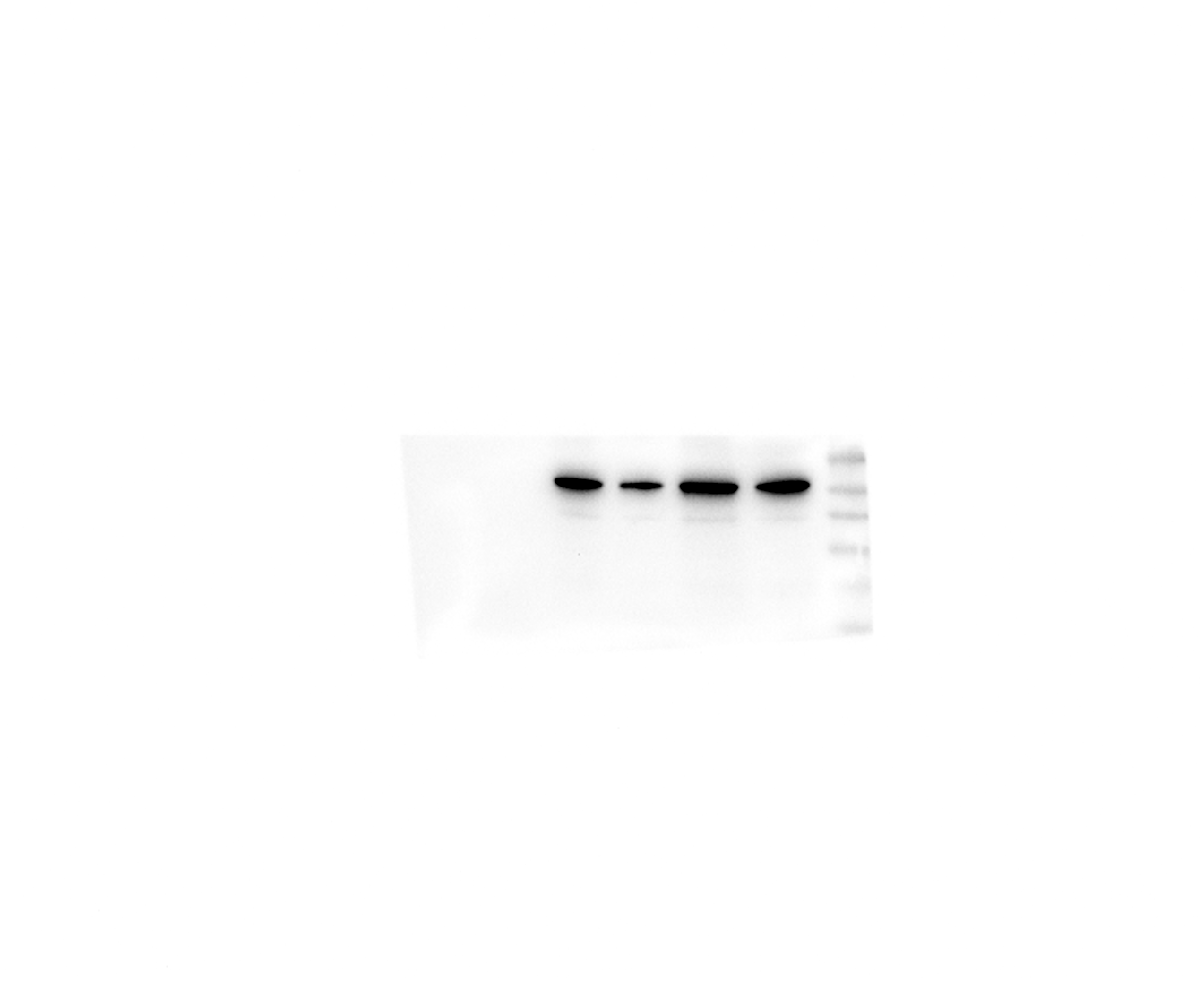

Supplement: File S1. Western Blot Data [file mmc3.zip › Western Blot Data/WB/Fig4A-Bx-PC-3-GLUT1.Tif]

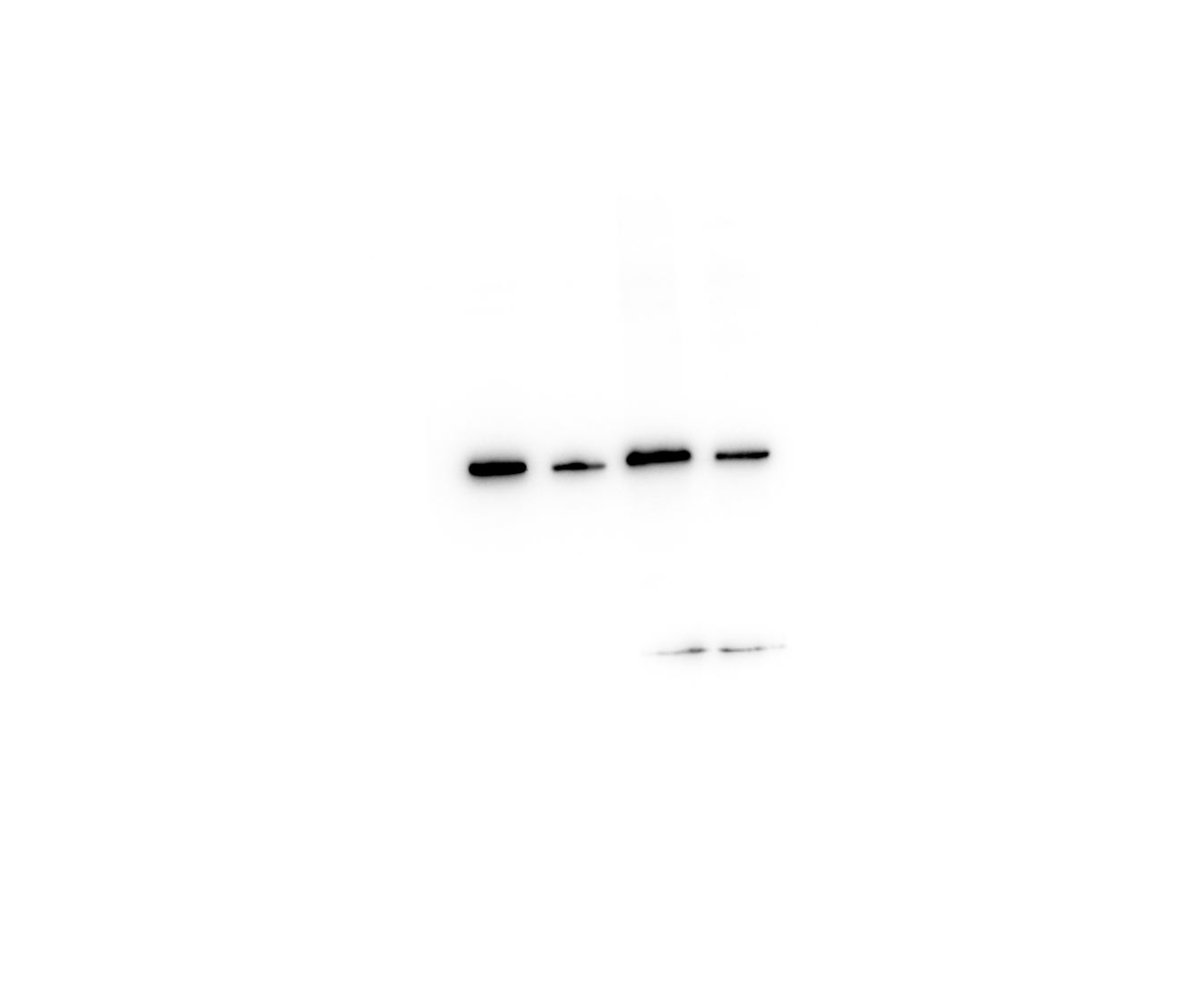

Supplement: File S1. Western Blot Data [file mmc3.zip › Western Blot Data/WB/Fig4A-Bx-PC-3-LDHA.Tif]

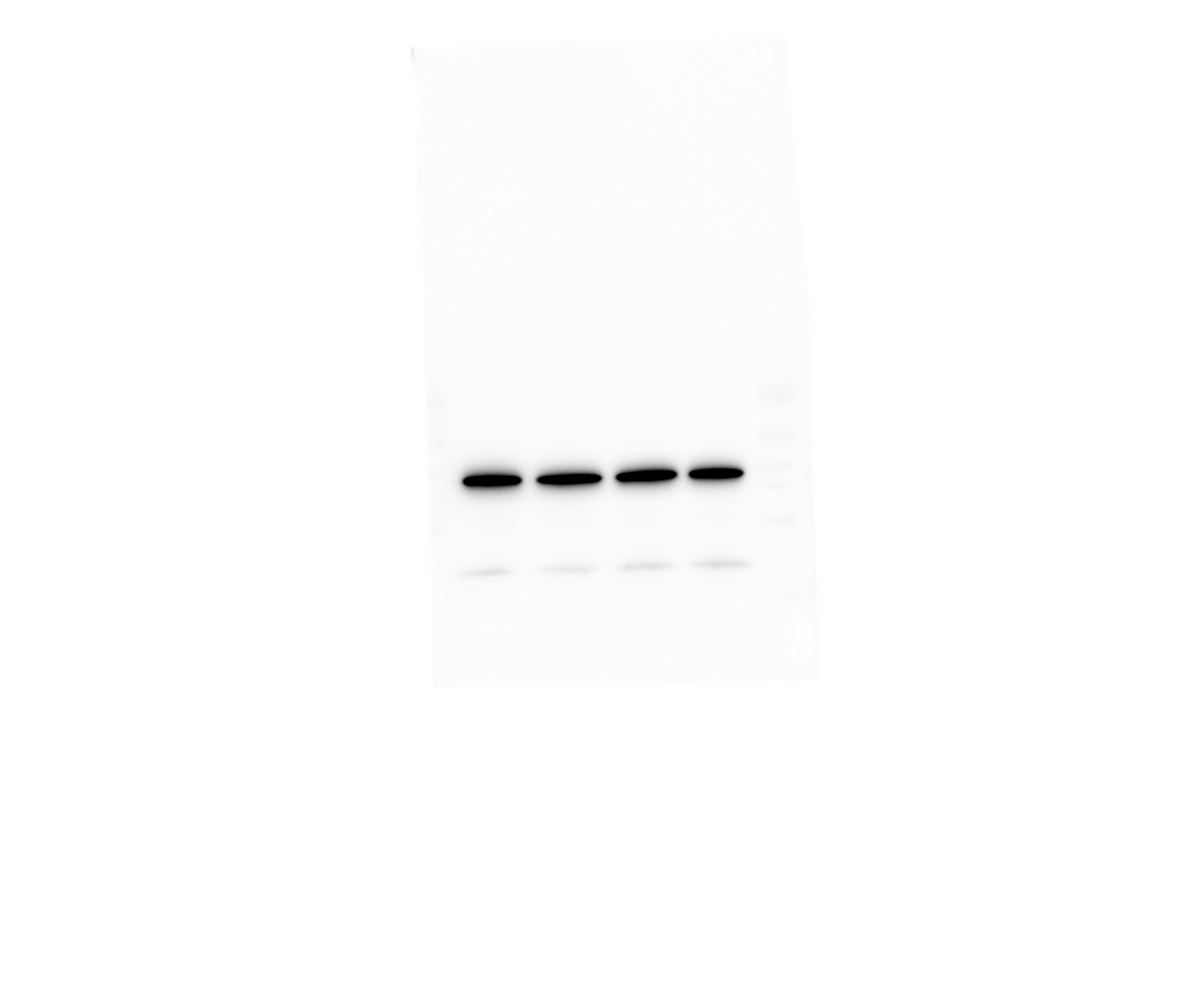

Supplement: File S1. Western Blot Data [file mmc3.zip › Western Blot Data/WB/Fig4A-PL45-GAPDH.Tif]

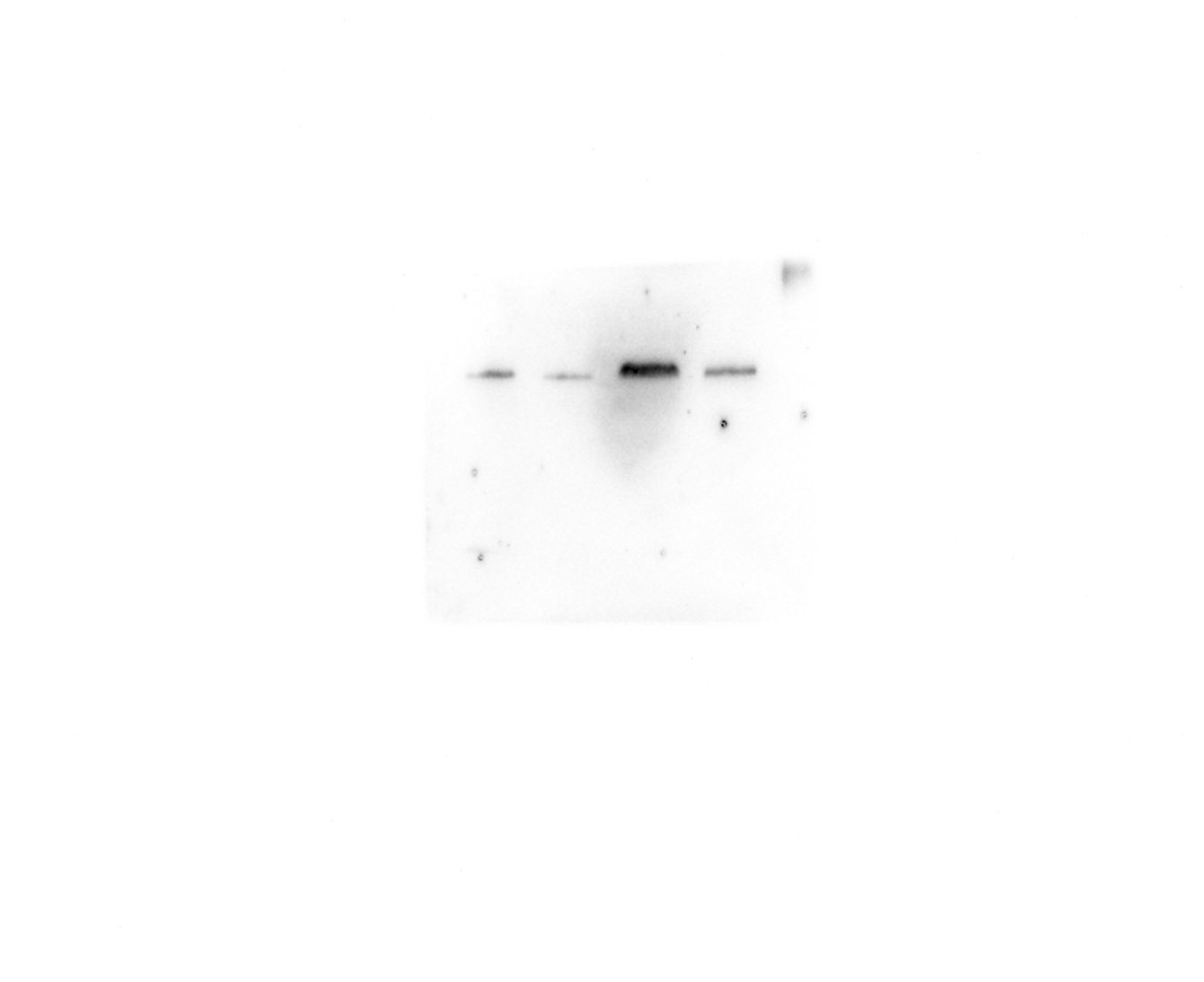

Supplement: File S1. Western Blot Data [file mmc3.zip › Western Blot Data/WB/Fig4A-PL45-GLUT1.Tif]

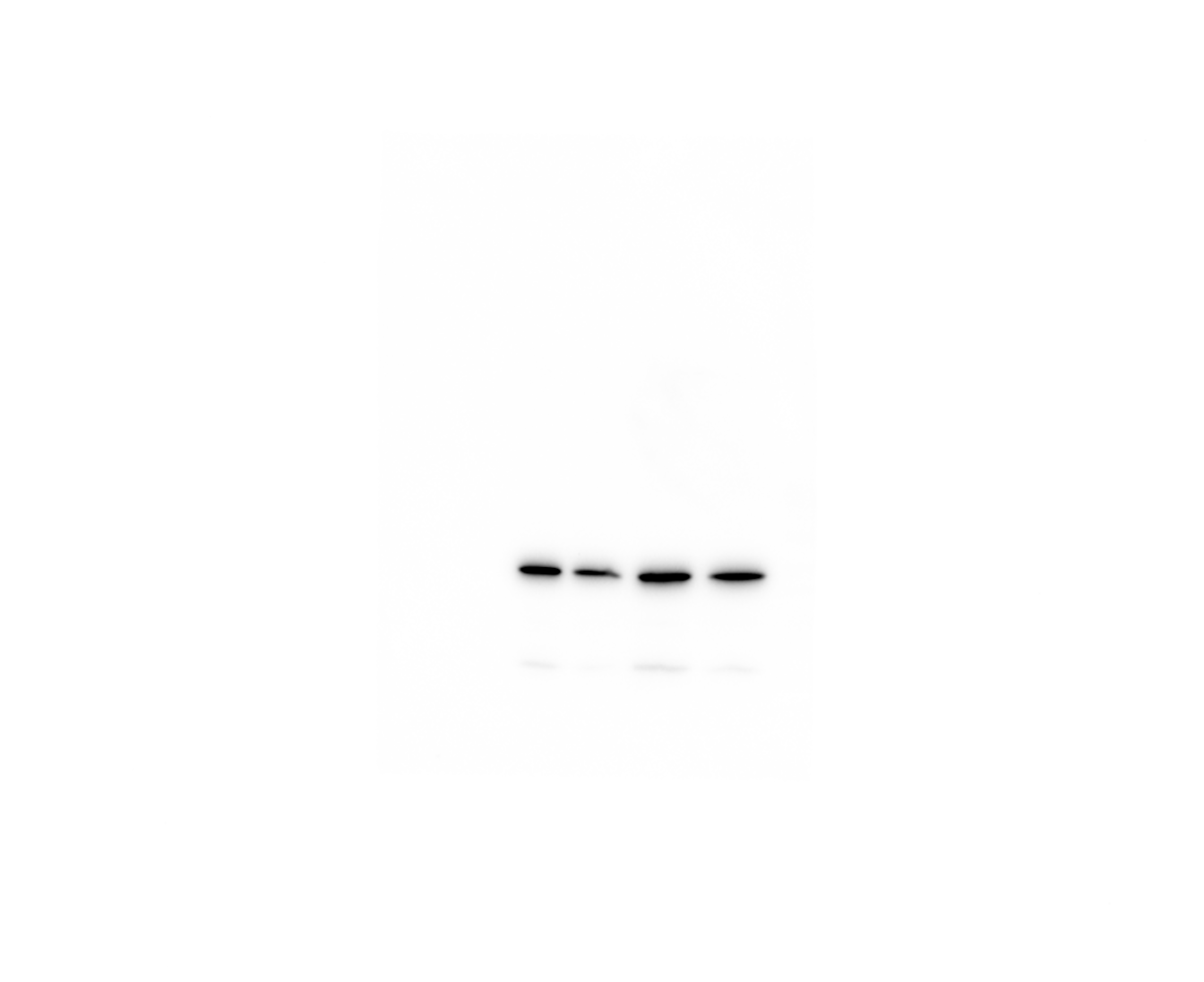

Supplement: File S1. Western Blot Data [file mmc3.zip › Western Blot Data/WB/Fig4A-PL45-LDHA.Tif]

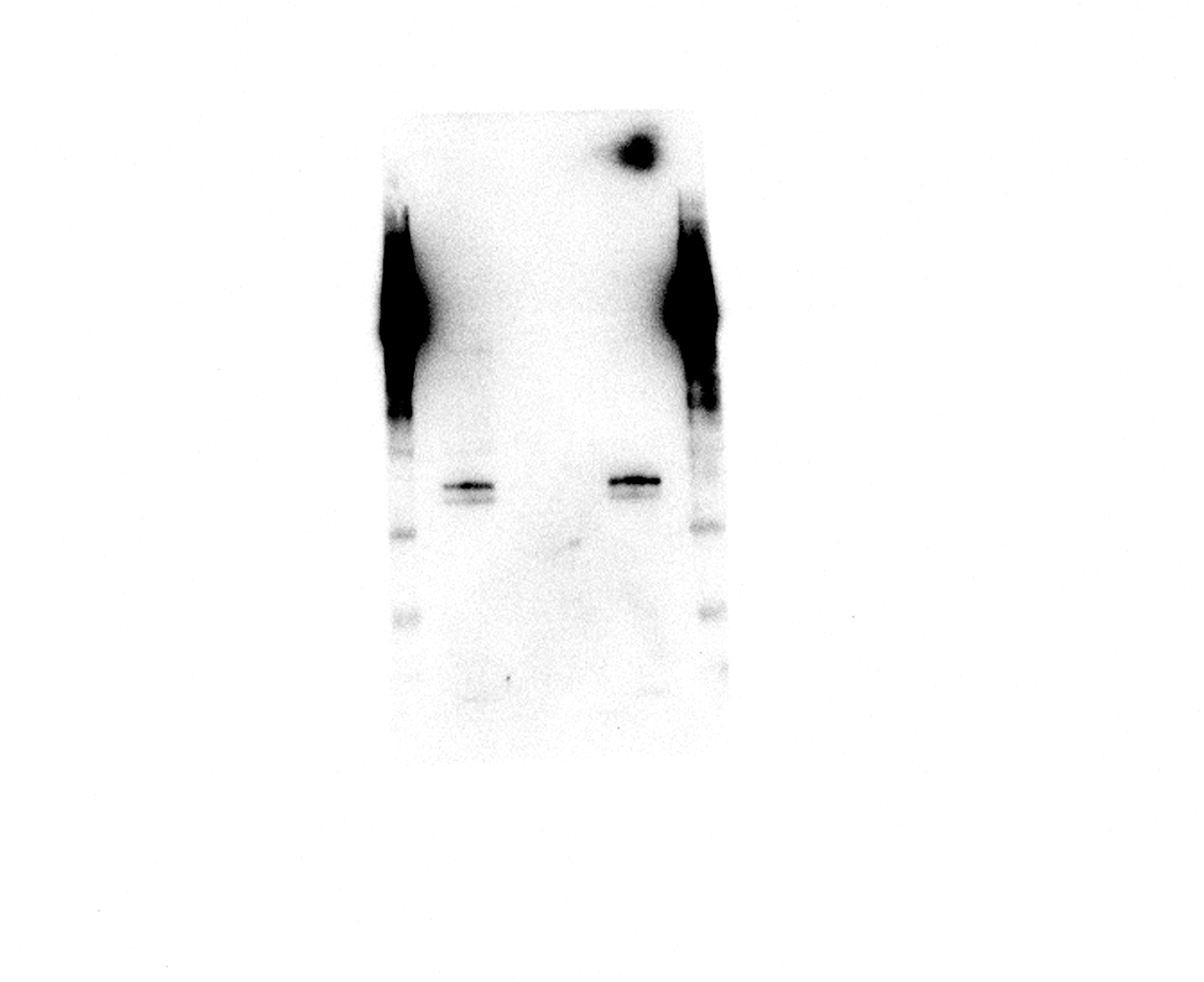

Supplement: File S1. Western Blot Data [file mmc3.zip › Western Blot Data/WB/Fig5D-Bx-PC-3--Menin-MEN1.Tif]

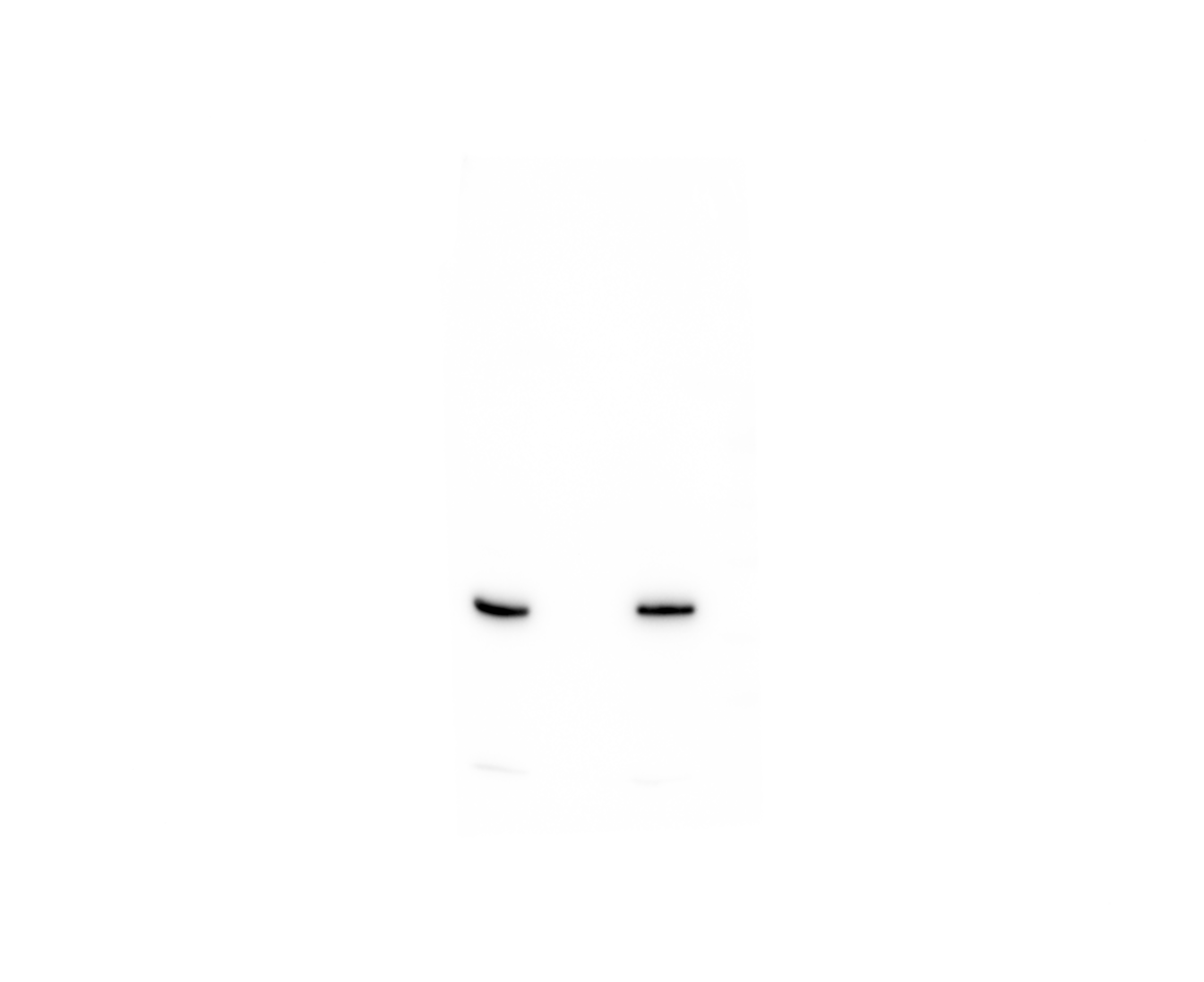

Supplement: File S1. Western Blot Data [file mmc3.zip › Western Blot Data/WB/Fig5D-Bx-PC-3--Menin-YBX1.Tif]

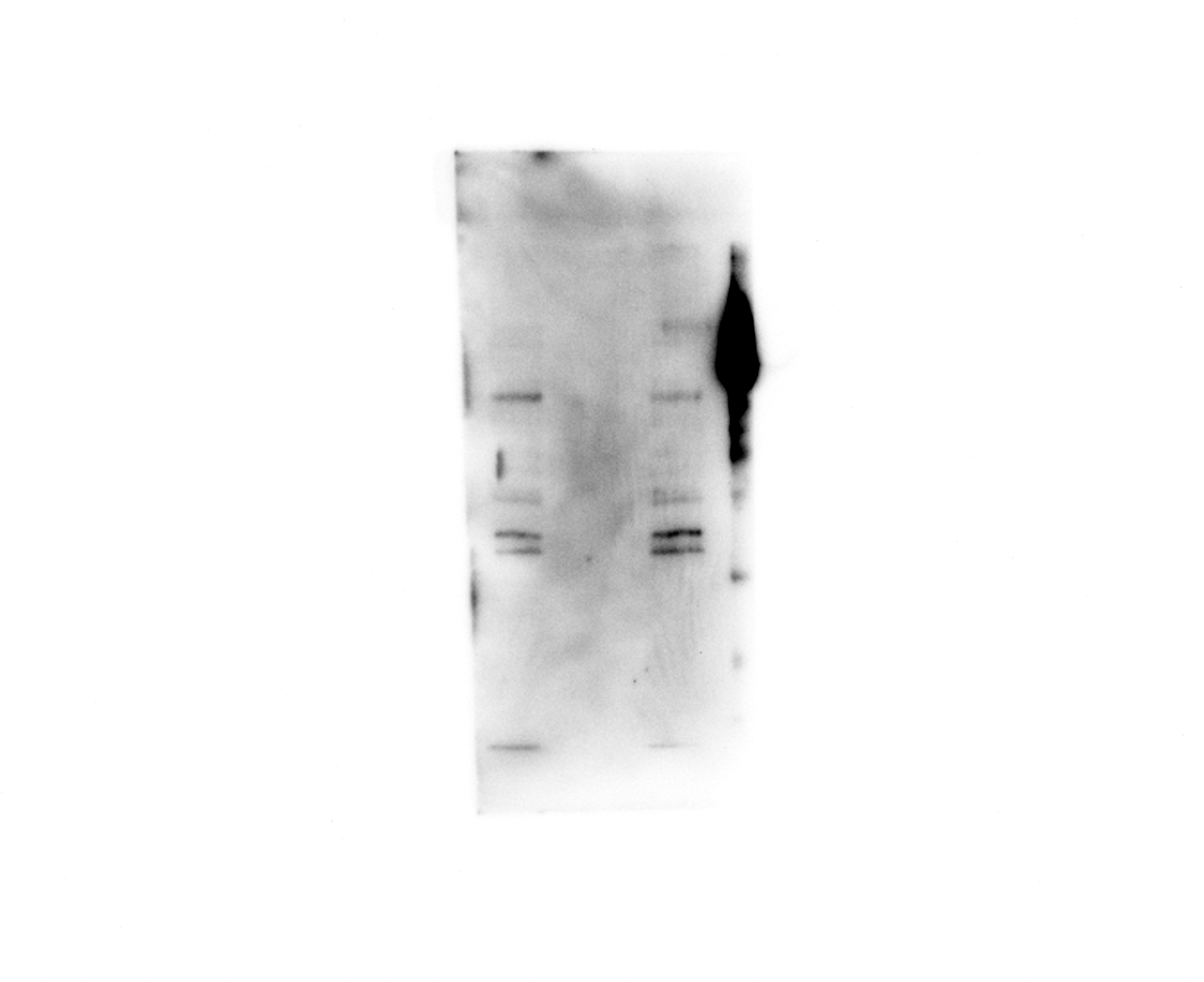

Supplement: File S1. Western Blot Data [file mmc3.zip › Western Blot Data/WB/Fig5D-Bx-PC-3-YBX1-MEN1.Tif]

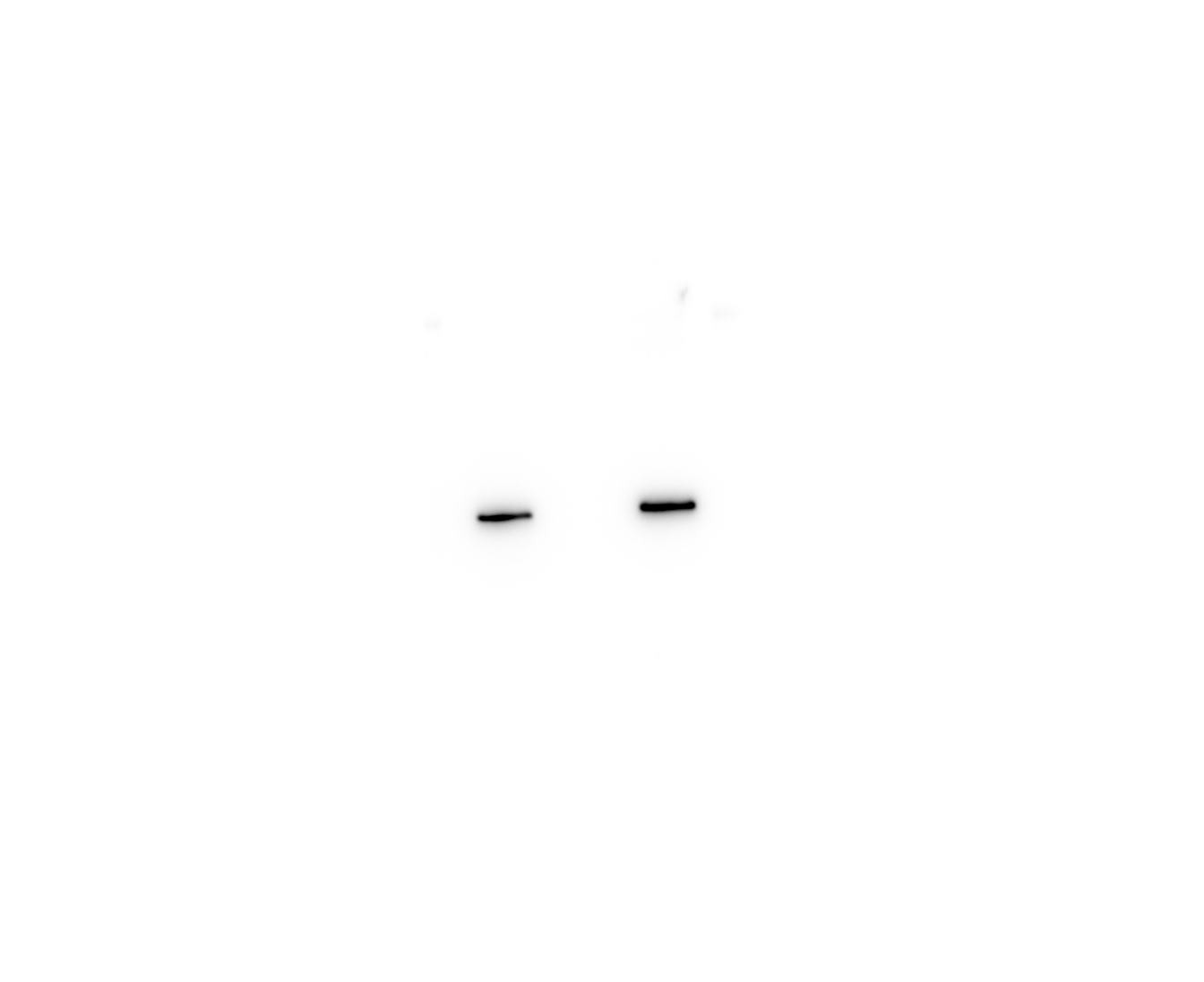

Supplement: File S1. Western Blot Data [file mmc3.zip › Western Blot Data/WB/Fig5D-Bx-PC-3-YBX1-YBX1.Tif]

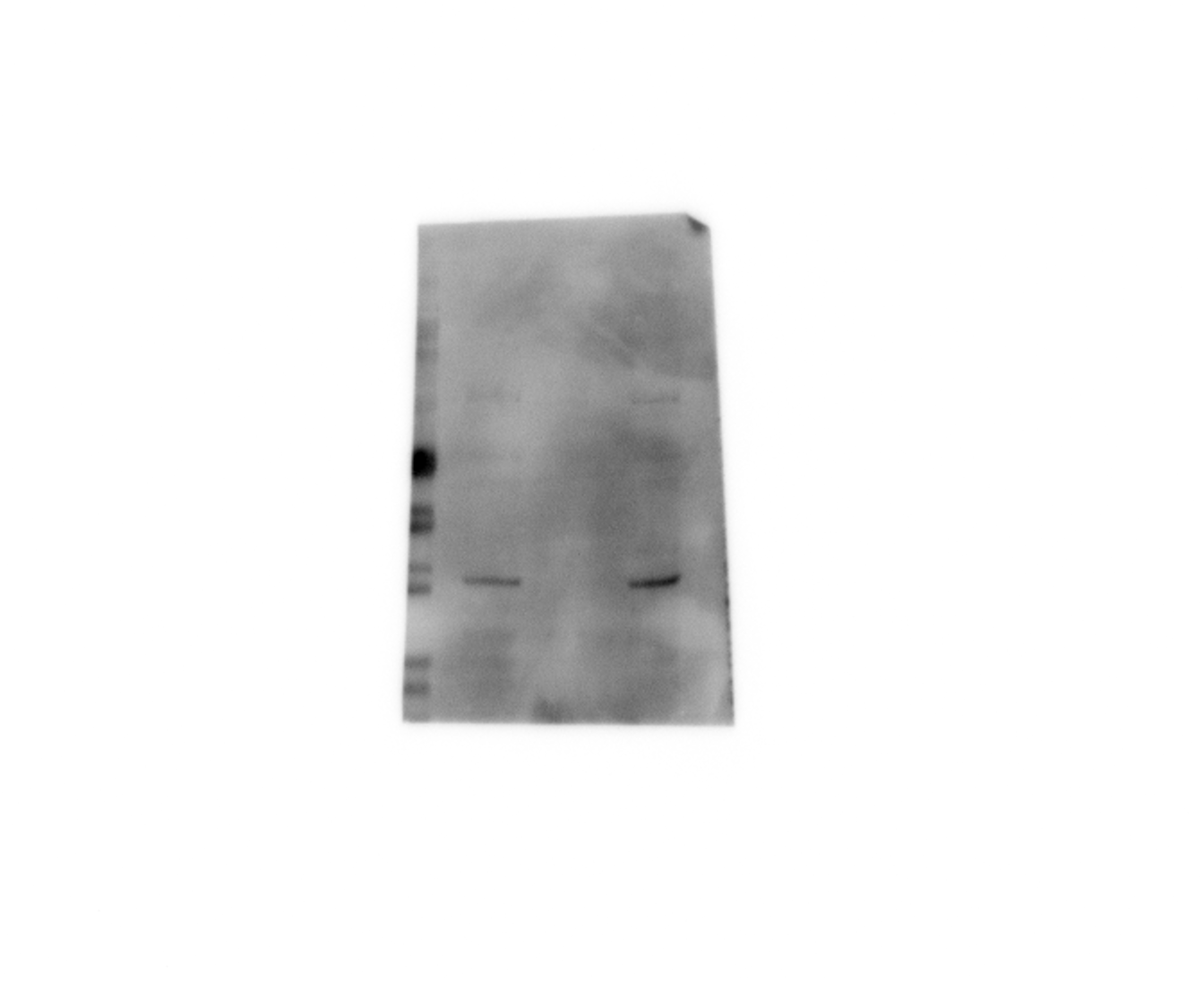

Supplement: File S1. Western Blot Data [file mmc3.zip › Western Blot Data/WB/Fig5D-PL45--Menin-MEN1.Tif]

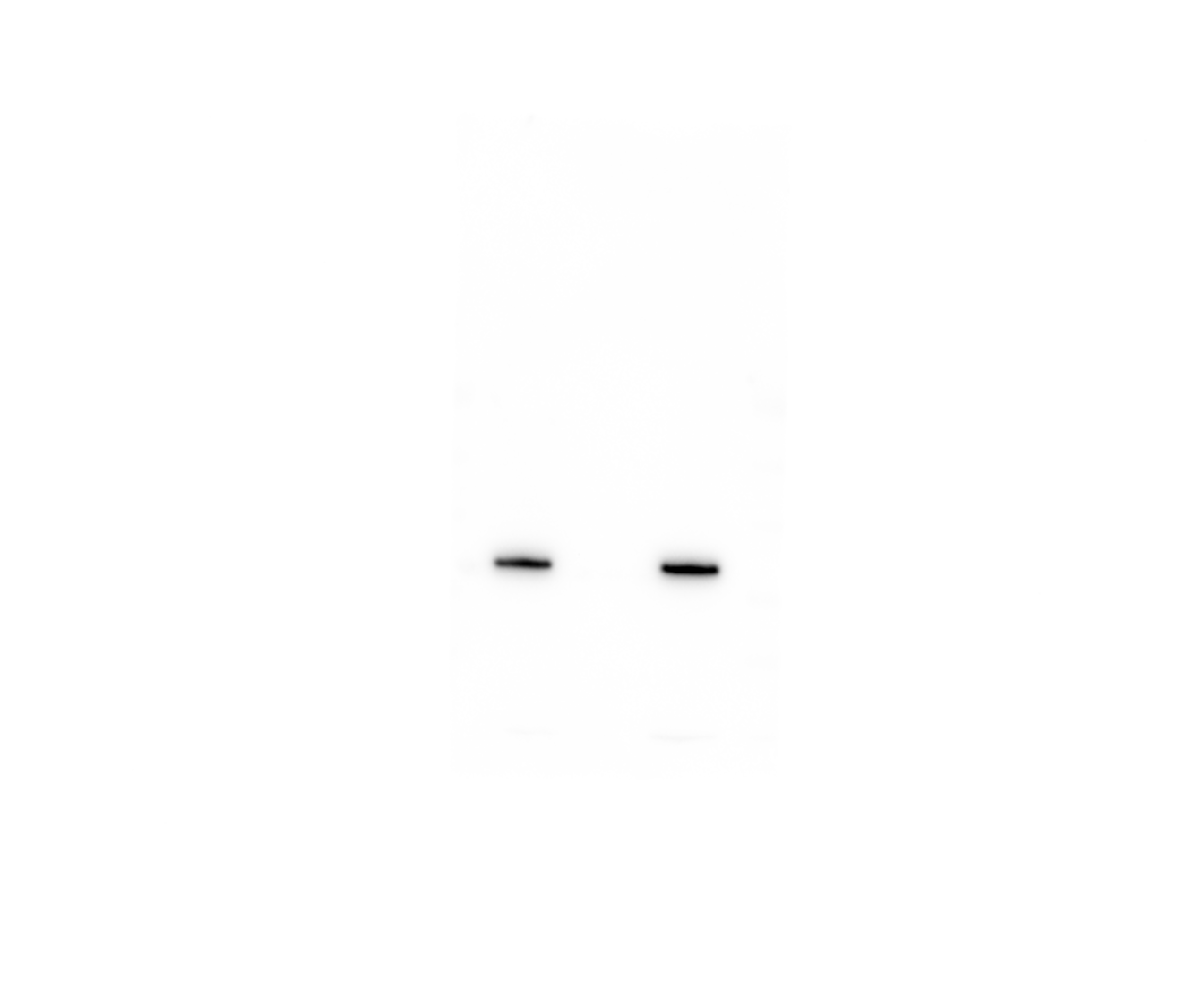

Supplement: File S1. Western Blot Data [file mmc3.zip › Western Blot Data/WB/Fig5D-PL45--Menin-YBX1.Tif]

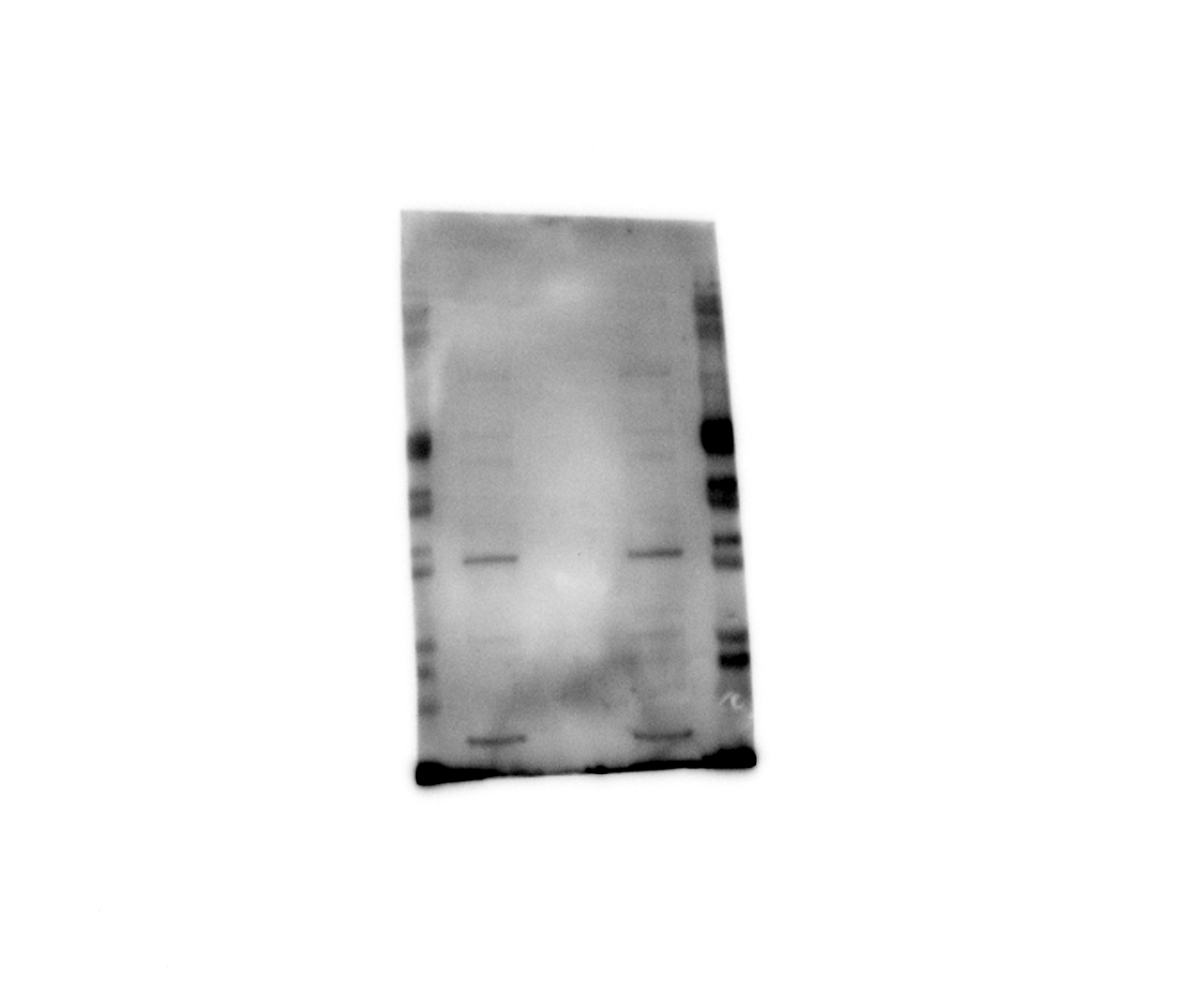

Supplement: File S1. Western Blot Data [file mmc3.zip › Western Blot Data/WB/Fig5D-PL45-YBX1-MEN1-1.Tif]

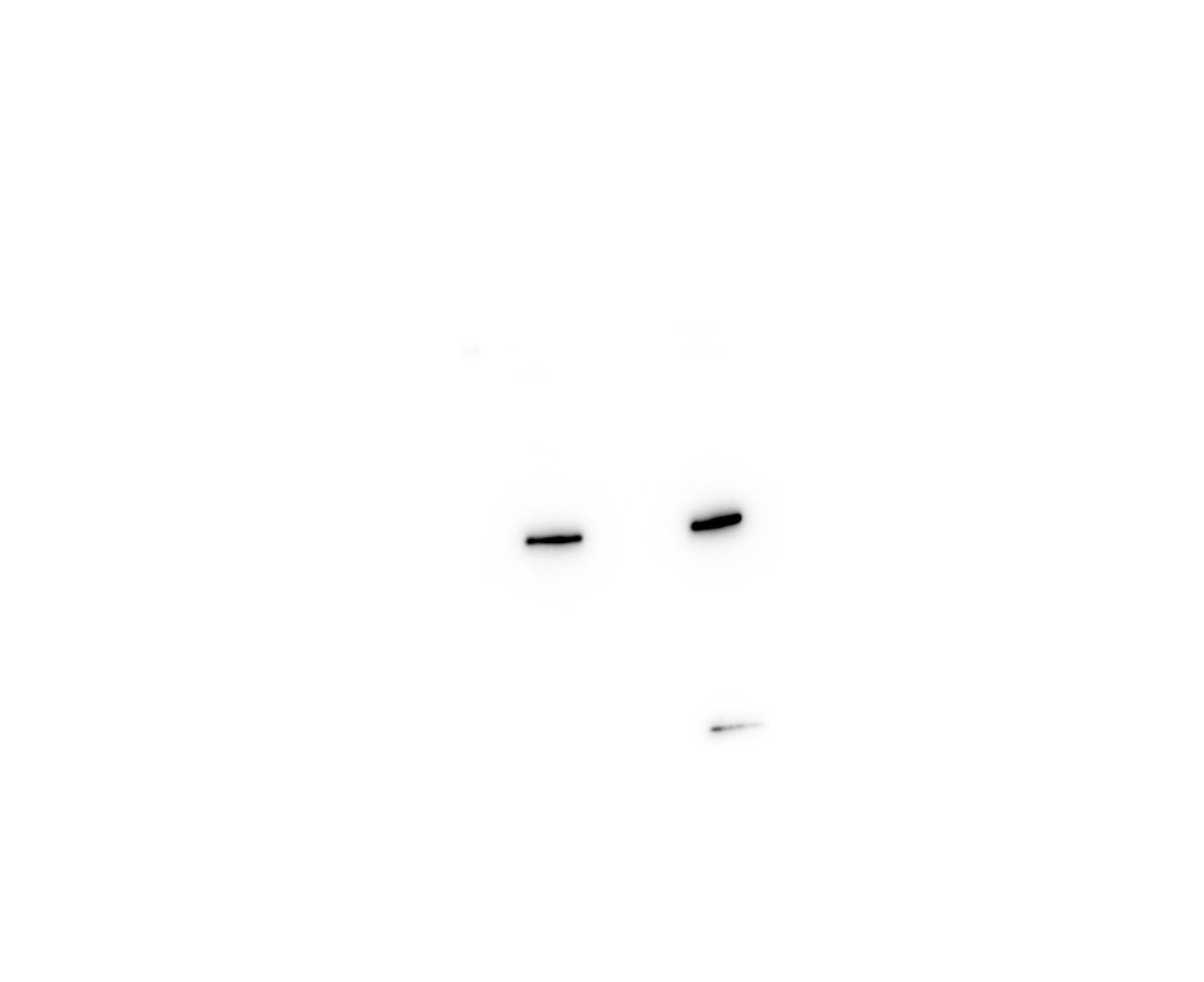

Supplement: File S1. Western Blot Data [file mmc3.zip › Western Blot Data/WB/Fig5D-PL45-YBX1-YBX1-1.Tif]

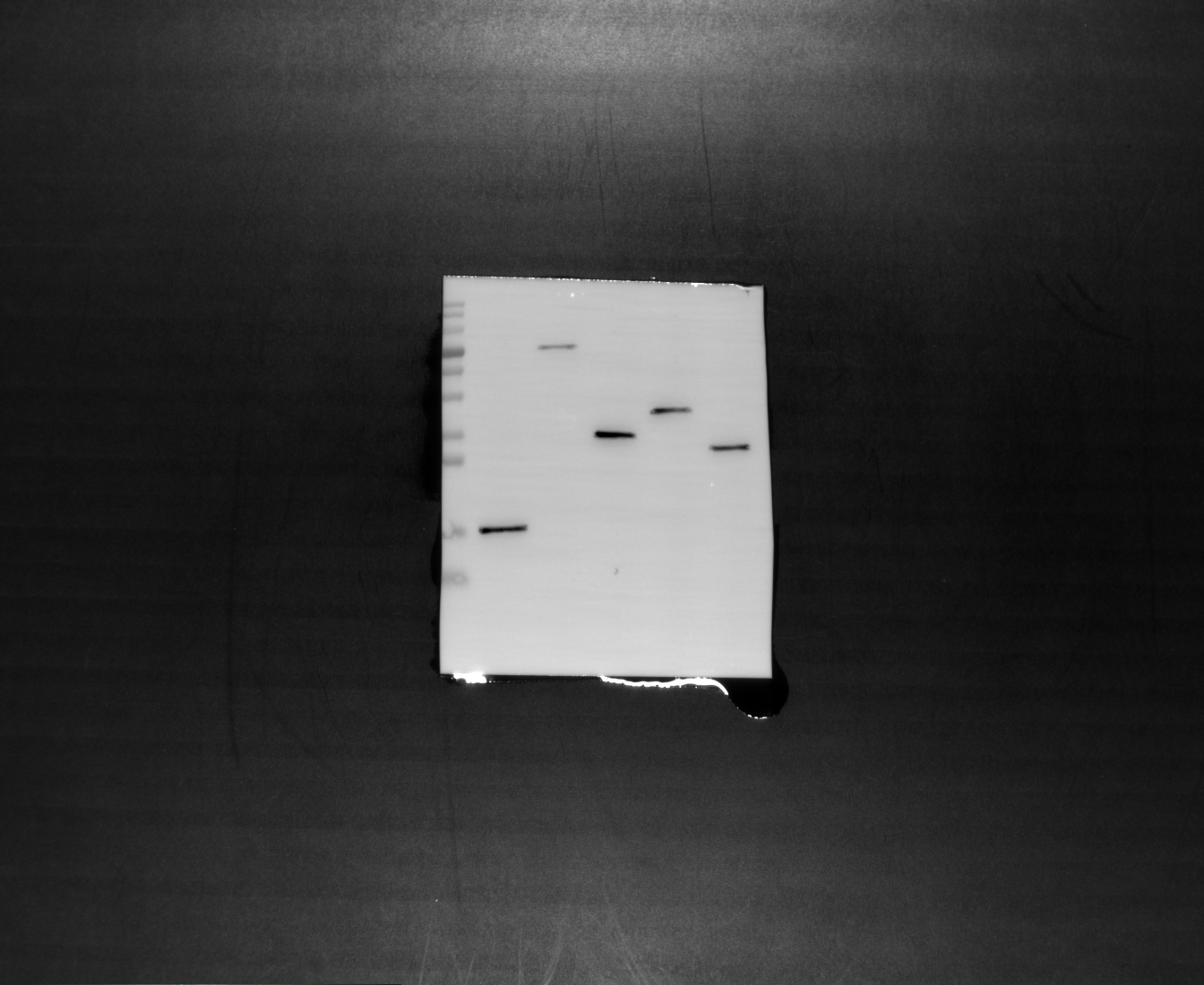

Supplement: File S1. Western Blot Data [file mmc3.zip › Western Blot Data/WB/Fig5F-GST-tag Merge.Tif]

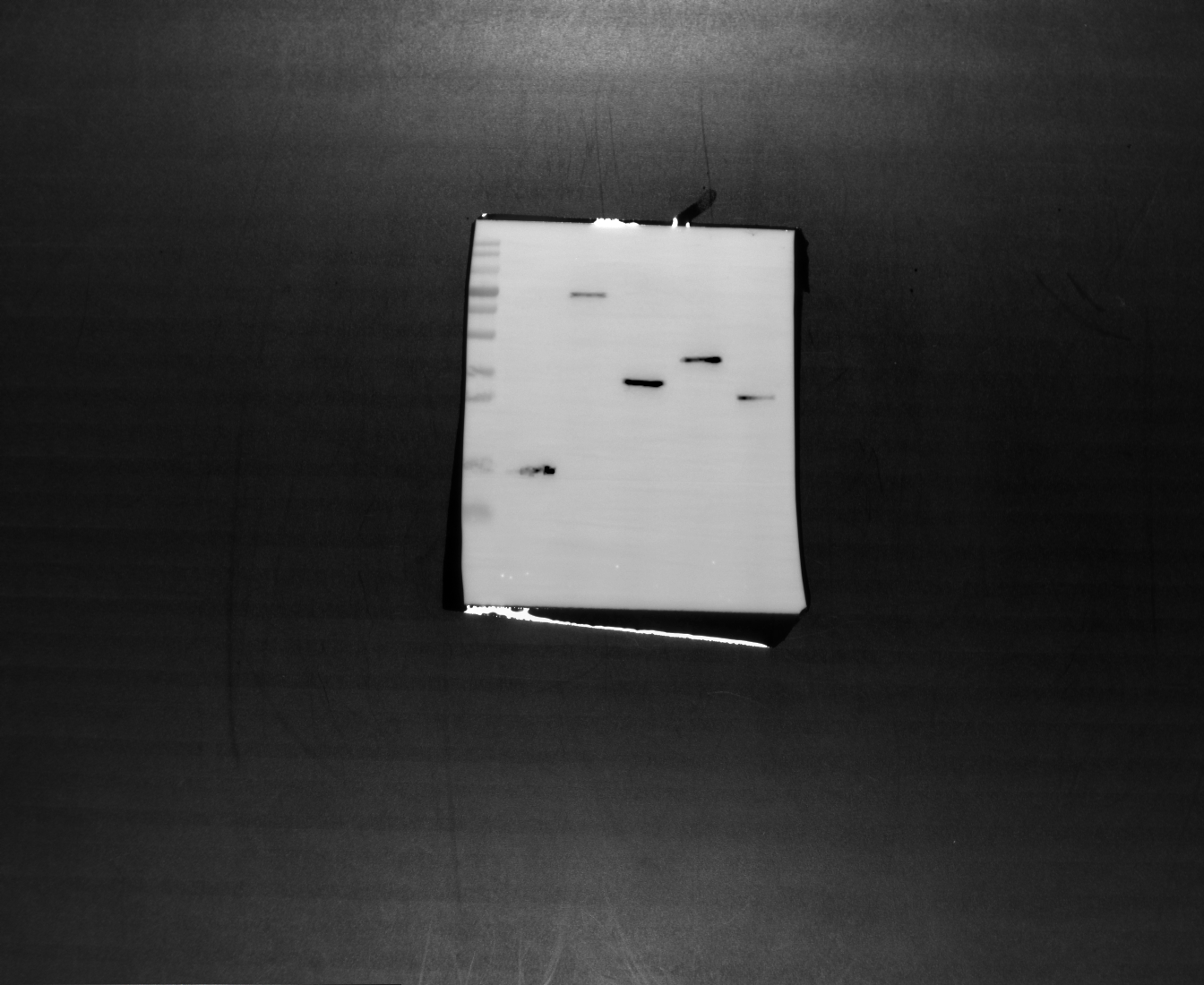

Supplement: File S1. Western Blot Data [file mmc3.zip › Western Blot Data/WB/Fig5F-GST-tag.Tif]

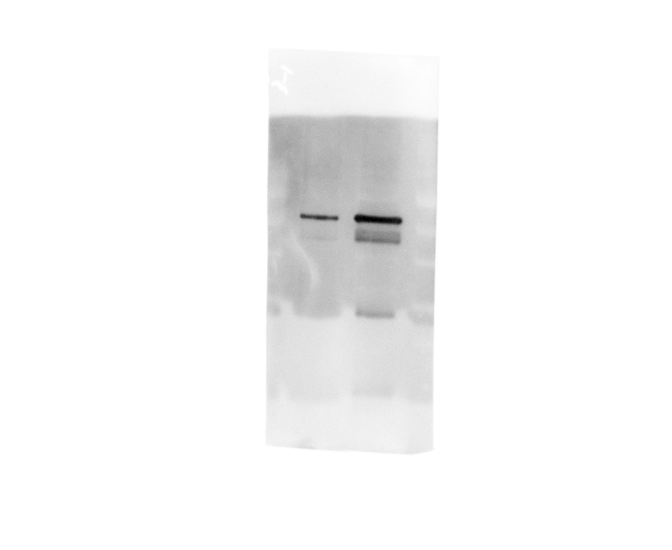

Supplement: File S1. Western Blot Data [file mmc3.zip › Western Blot Data/WB/Fig5F-GST.tif]

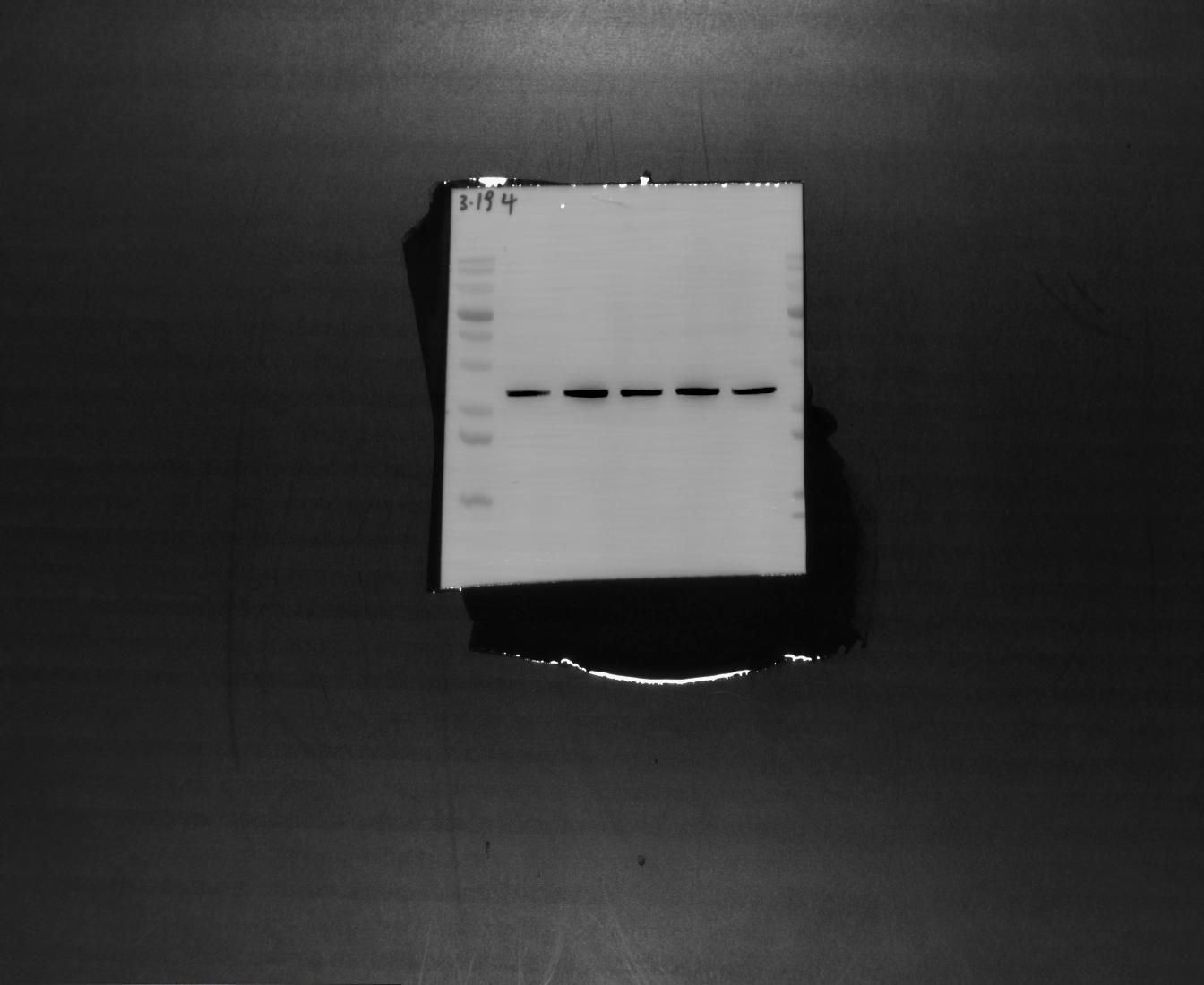

Supplement: File S1. Western Blot Data [file mmc3.zip › Western Blot Data/WB/Fig5F-HA-tag (2).Tif]

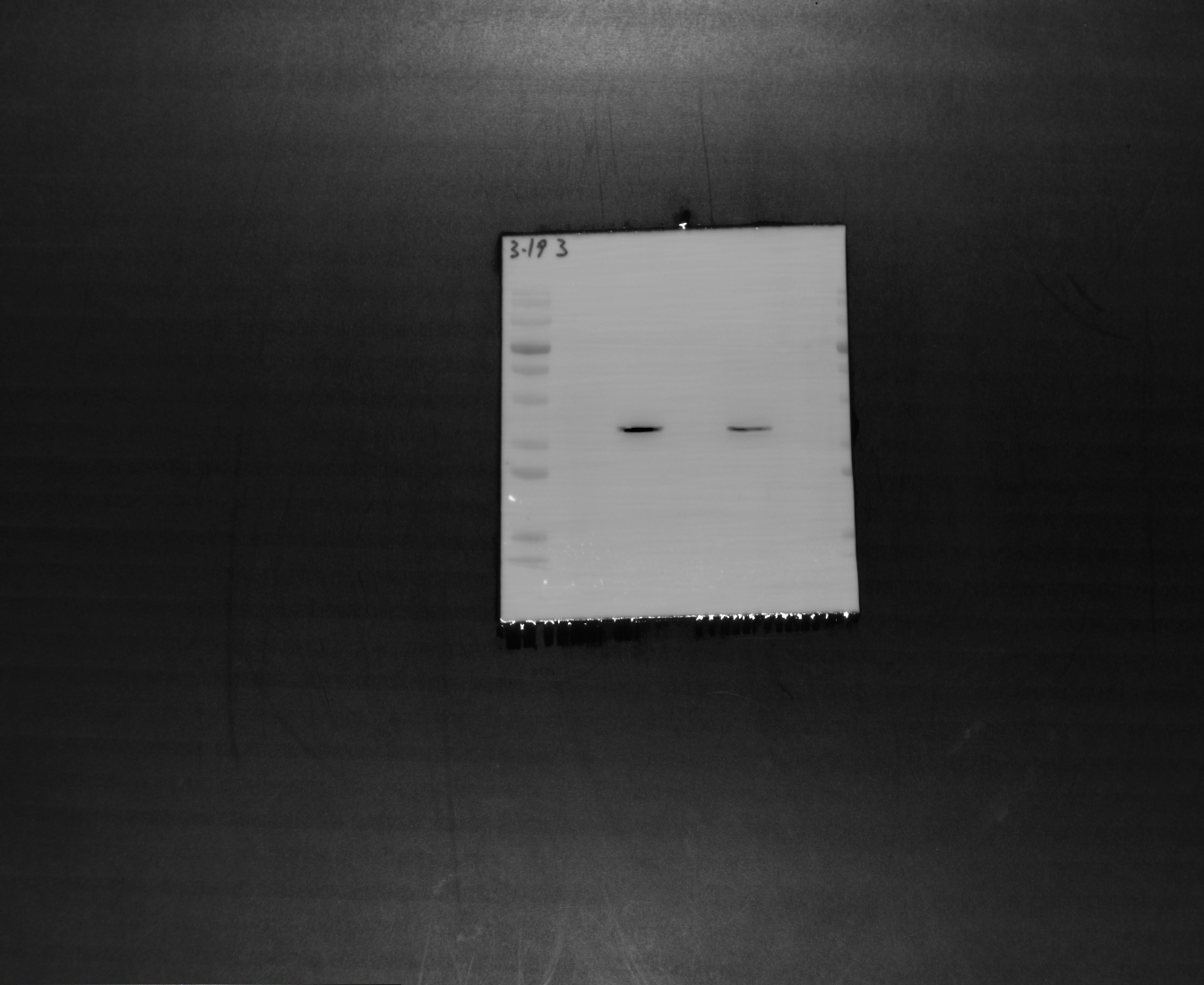

Supplement: File S1. Western Blot Data [file mmc3.zip › Western Blot Data/WB/Fig5F-HA-tag.Tif]

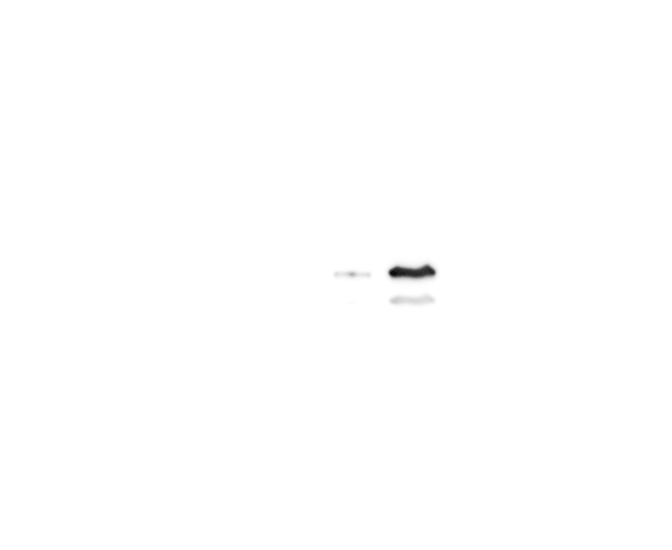

Supplement: File S1. Western Blot Data [file mmc3.zip › Western Blot Data/WB/Fig5F-YBX1 IP.tif]

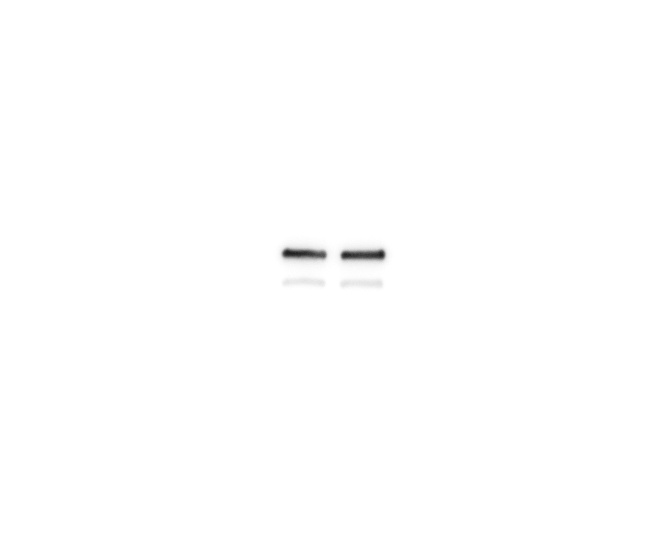

Supplement: File S1. Western Blot Data [file mmc3.zip › Western Blot Data/WB/Fig5F-YBX1 Input.tif]

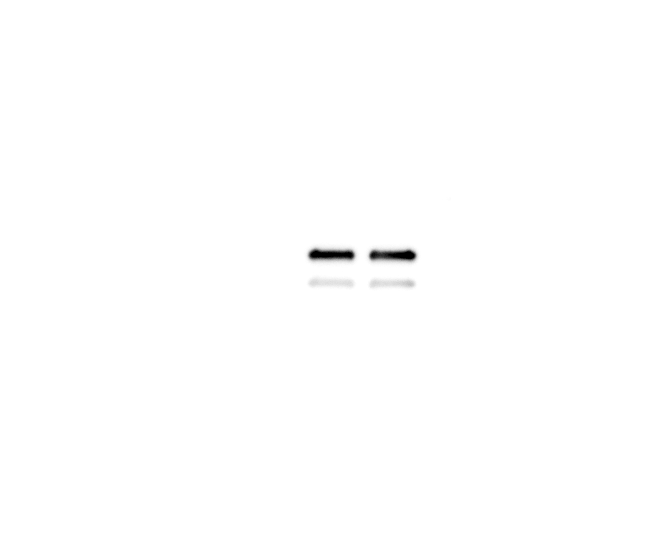

Supplement: File S1. Western Blot Data [file mmc3.zip › Western Blot Data/WB/Fig6A--cytopiasmGAPDH.tif]

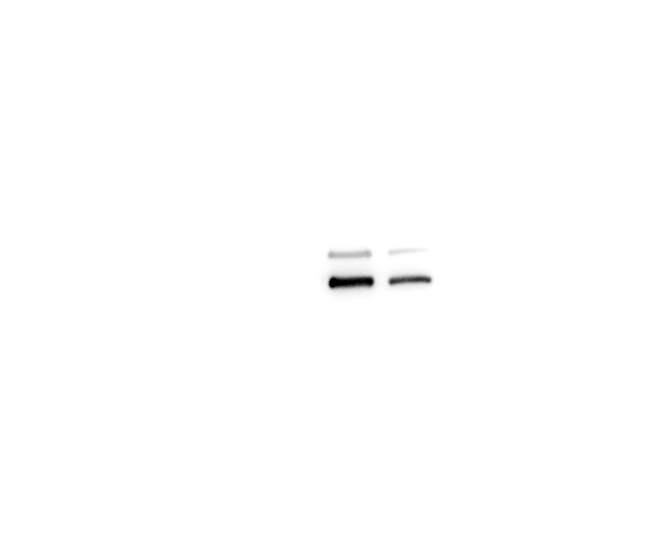

Supplement: File S1. Western Blot Data [file mmc3.zip › Western Blot Data/WB/Fig6A--cytopiasmYBX1 .tif]

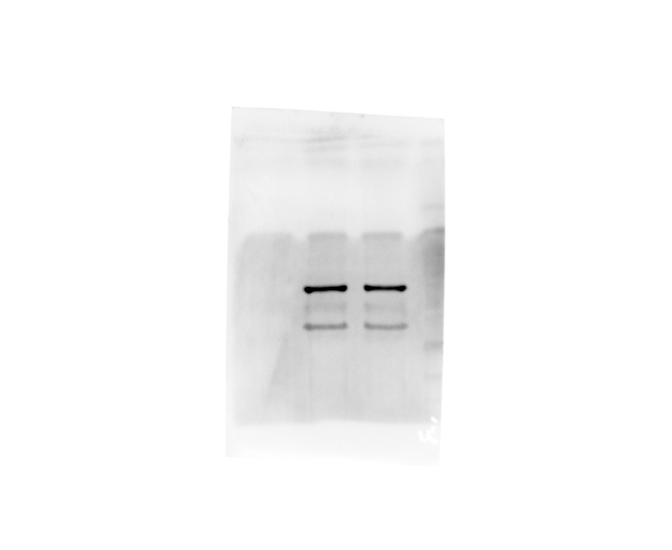

Supplement: File S1. Western Blot Data [file mmc3.zip › Western Blot Data/WB/Fig6A-nucleus-H3.tif]

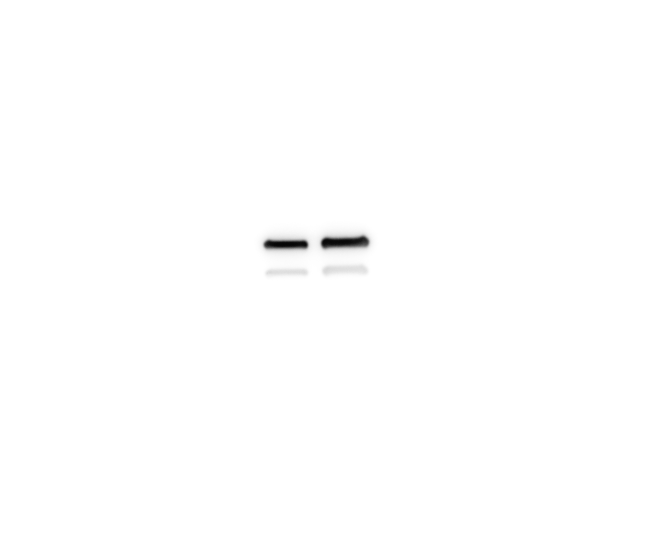

Supplement: File S1. Western Blot Data [file mmc3.zip › Western Blot Data/WB/Fig6A-nucleus-YBX1.tif]

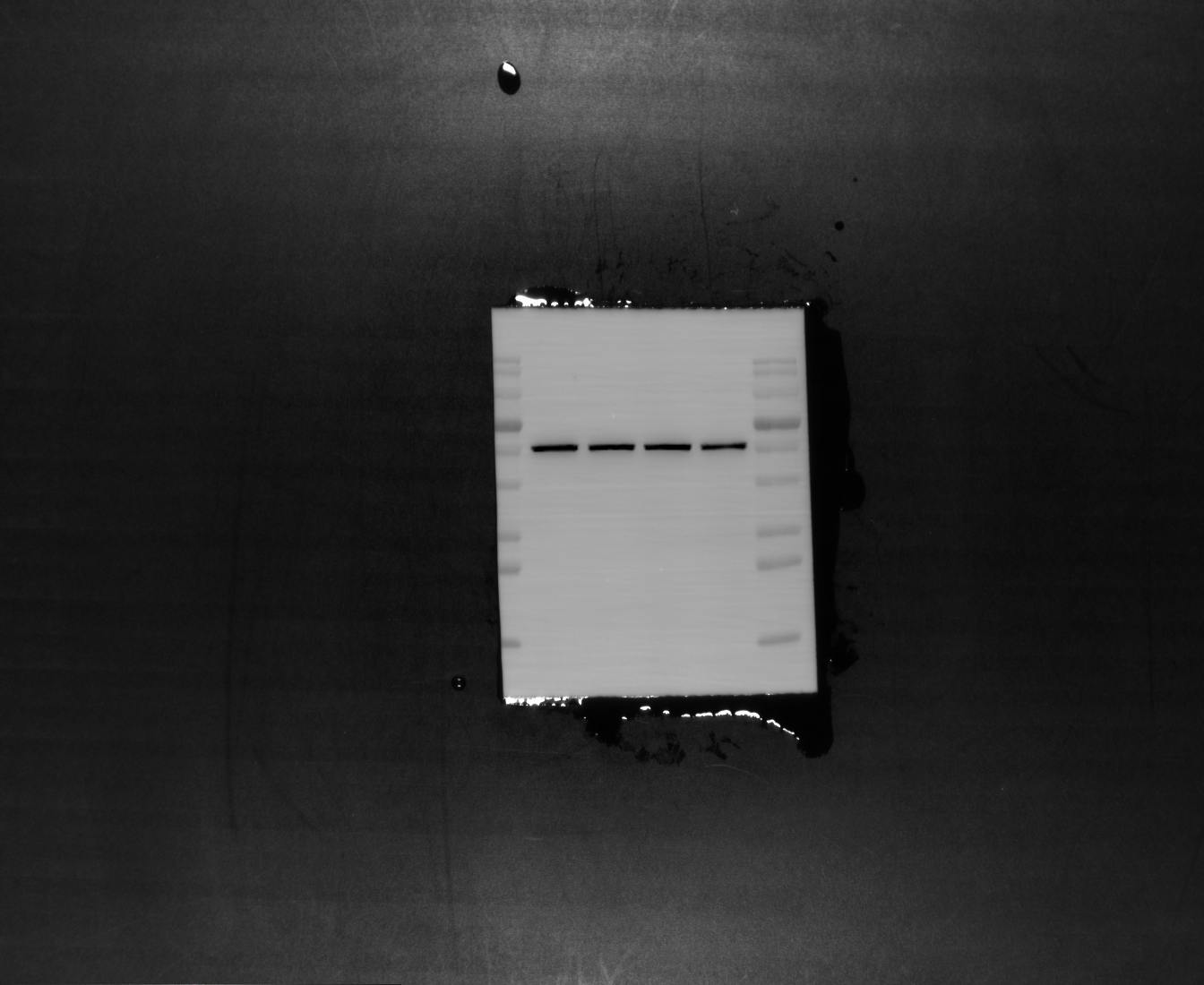

Supplement: File S1. Western Blot Data [file mmc3.zip › Western Blot Data/WB/Fig6C-Bx-PC-3-Lamin B1.Tif]

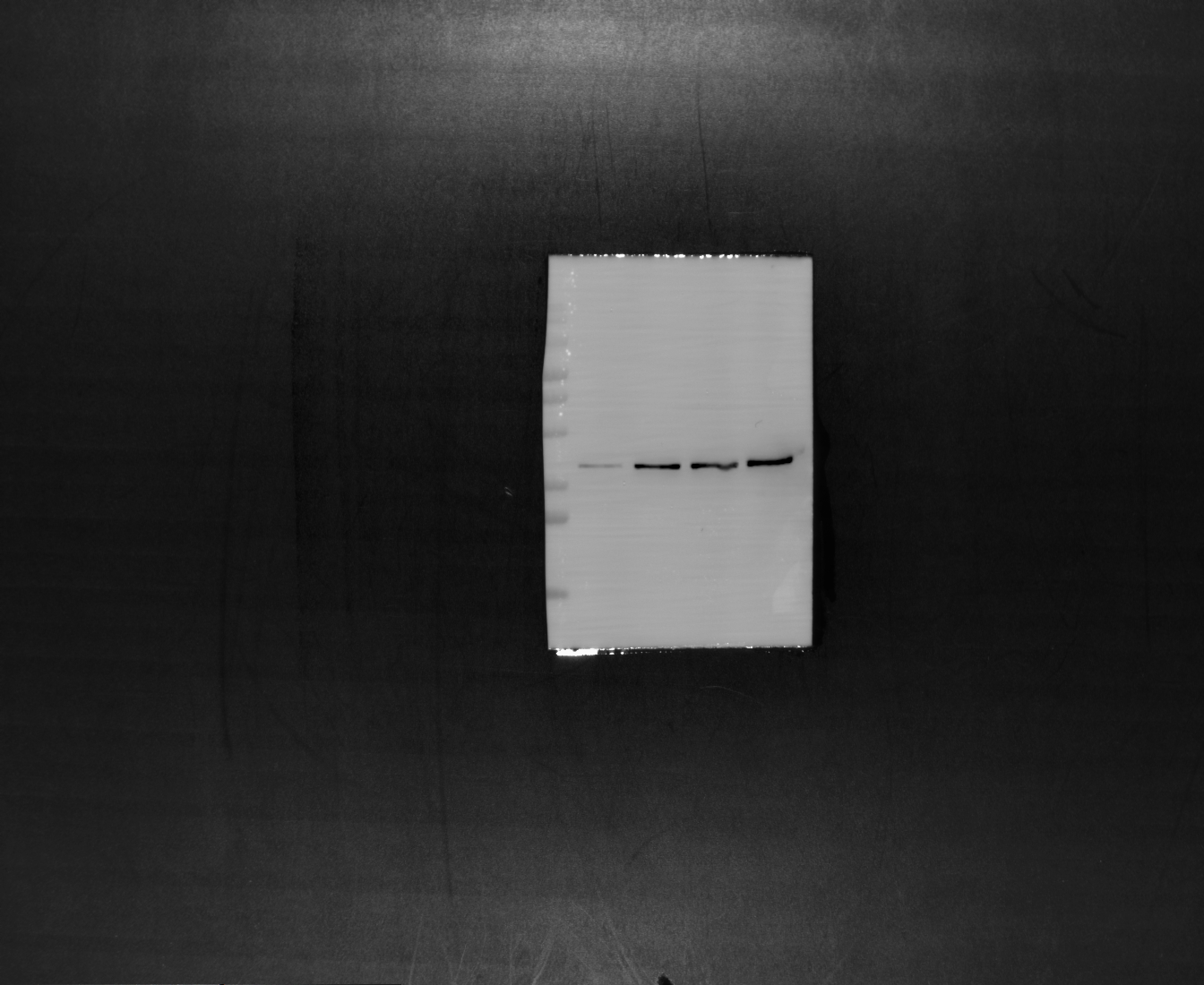

Supplement: File S1. Western Blot Data [file mmc3.zip › Western Blot Data/WB/Fig6C-Bx-PC-3-NC--YBX1.Tif]

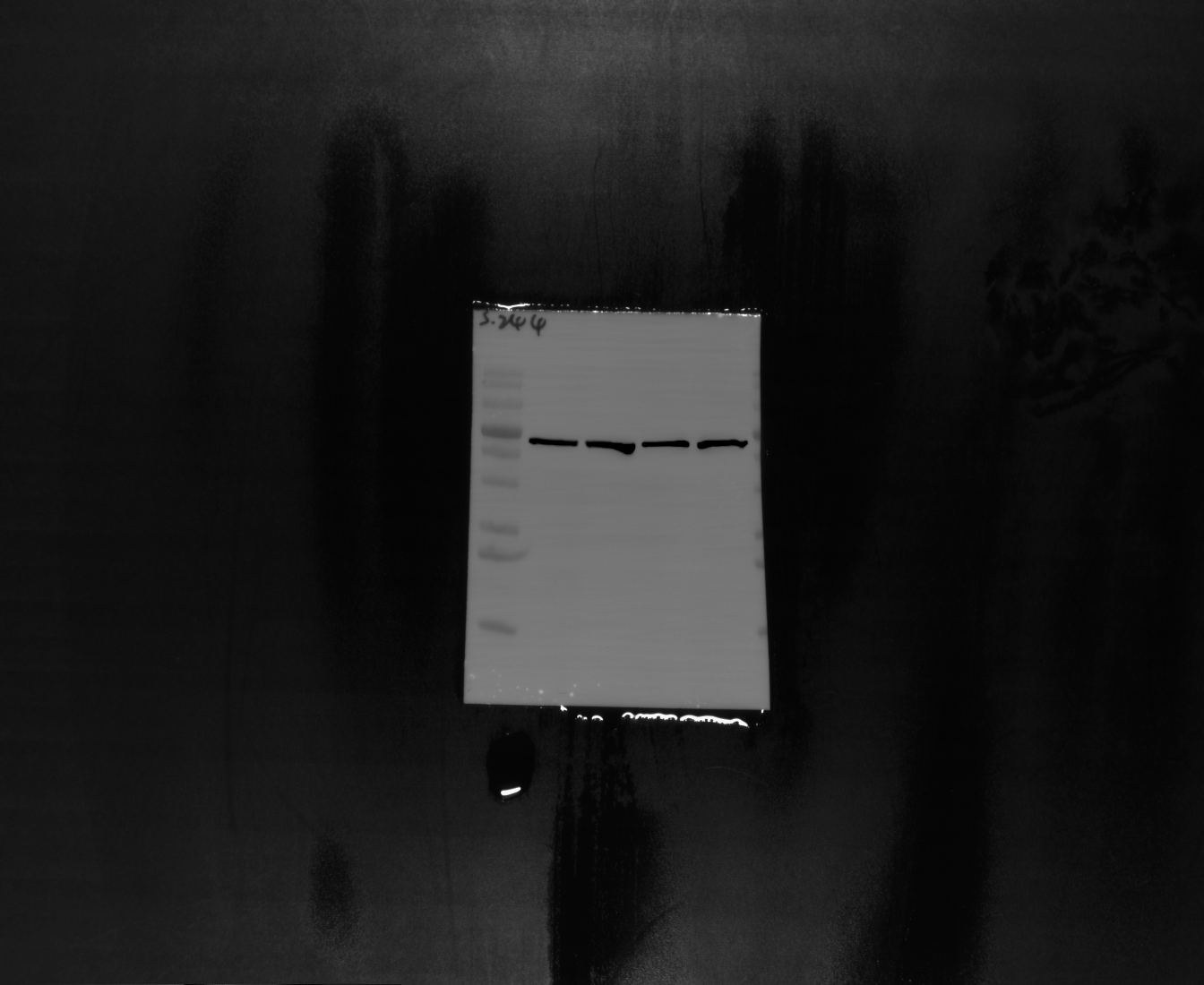

Supplement: File S1. Western Blot Data [file mmc3.zip › Western Blot Data/WB/Fig6C-Bx-PC-3-NC-Lamin B1 Merge.Tif]

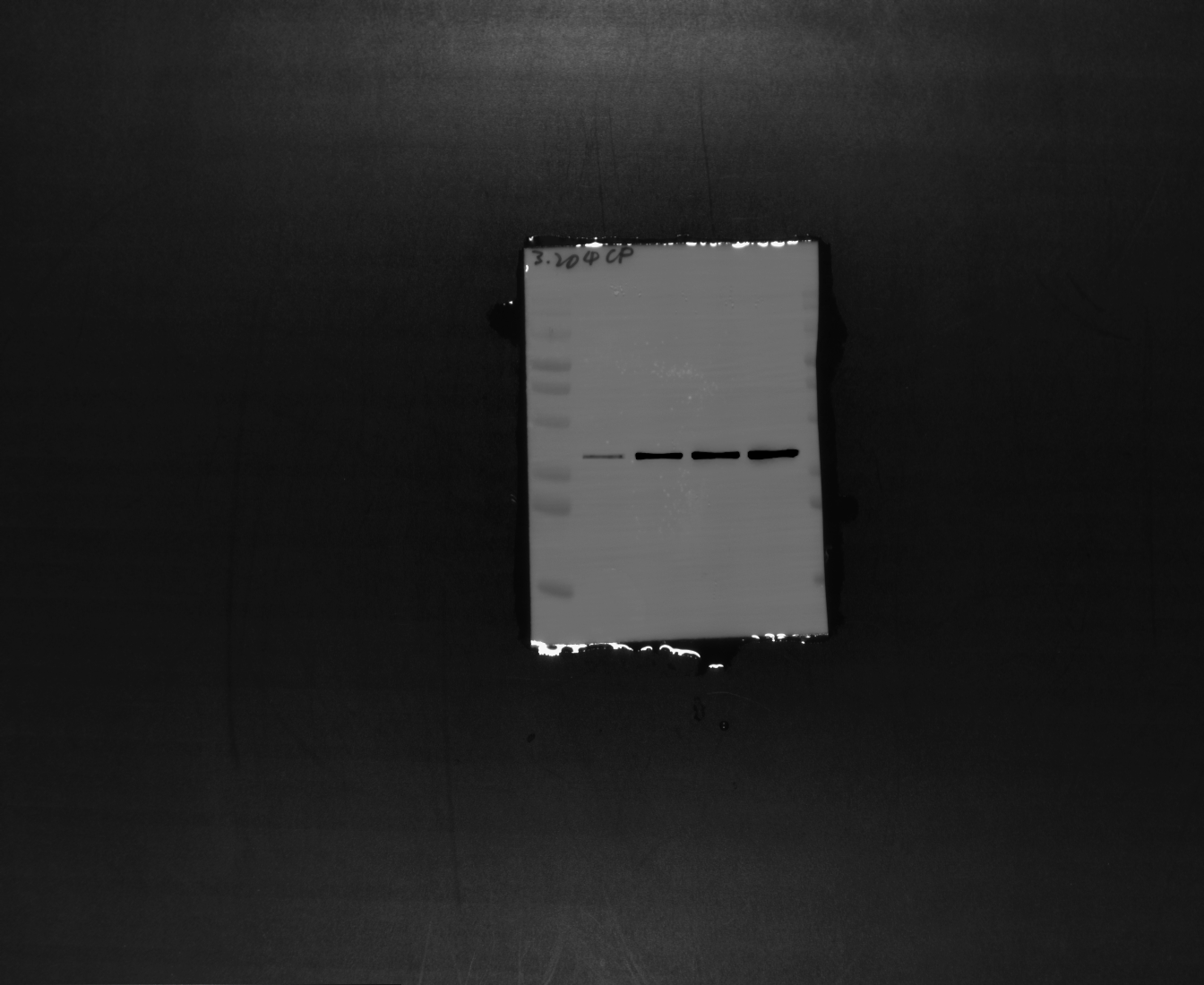

Supplement: File S1. Western Blot Data [file mmc3.zip › Western Blot Data/WB/Fig6C-Bx-PC-3-YBX1.Tif]

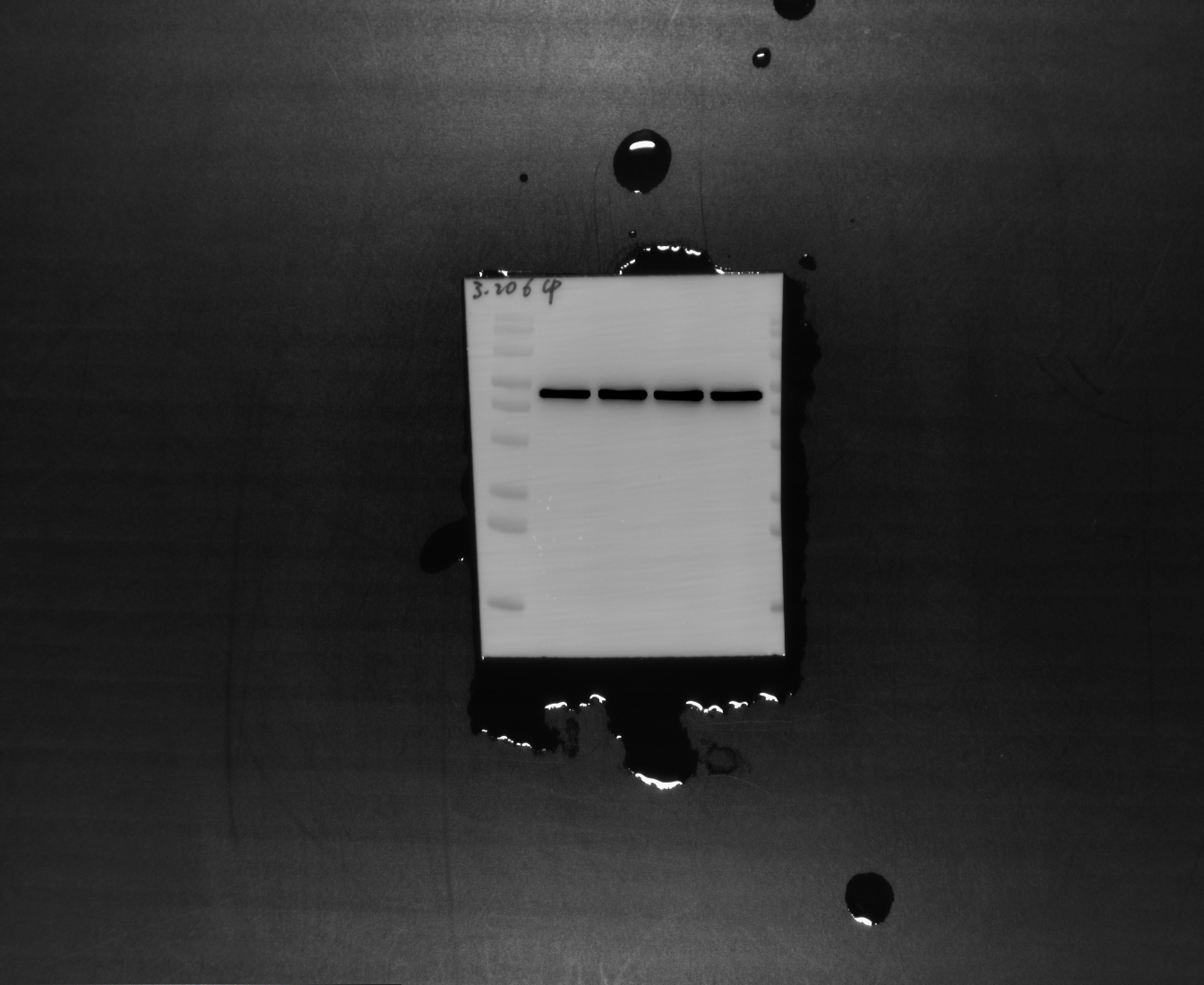

Supplement: File S1. Western Blot Data [file mmc3.zip › Western Blot Data/WB/Fig6C-PL45-Lamin B1.Tif]

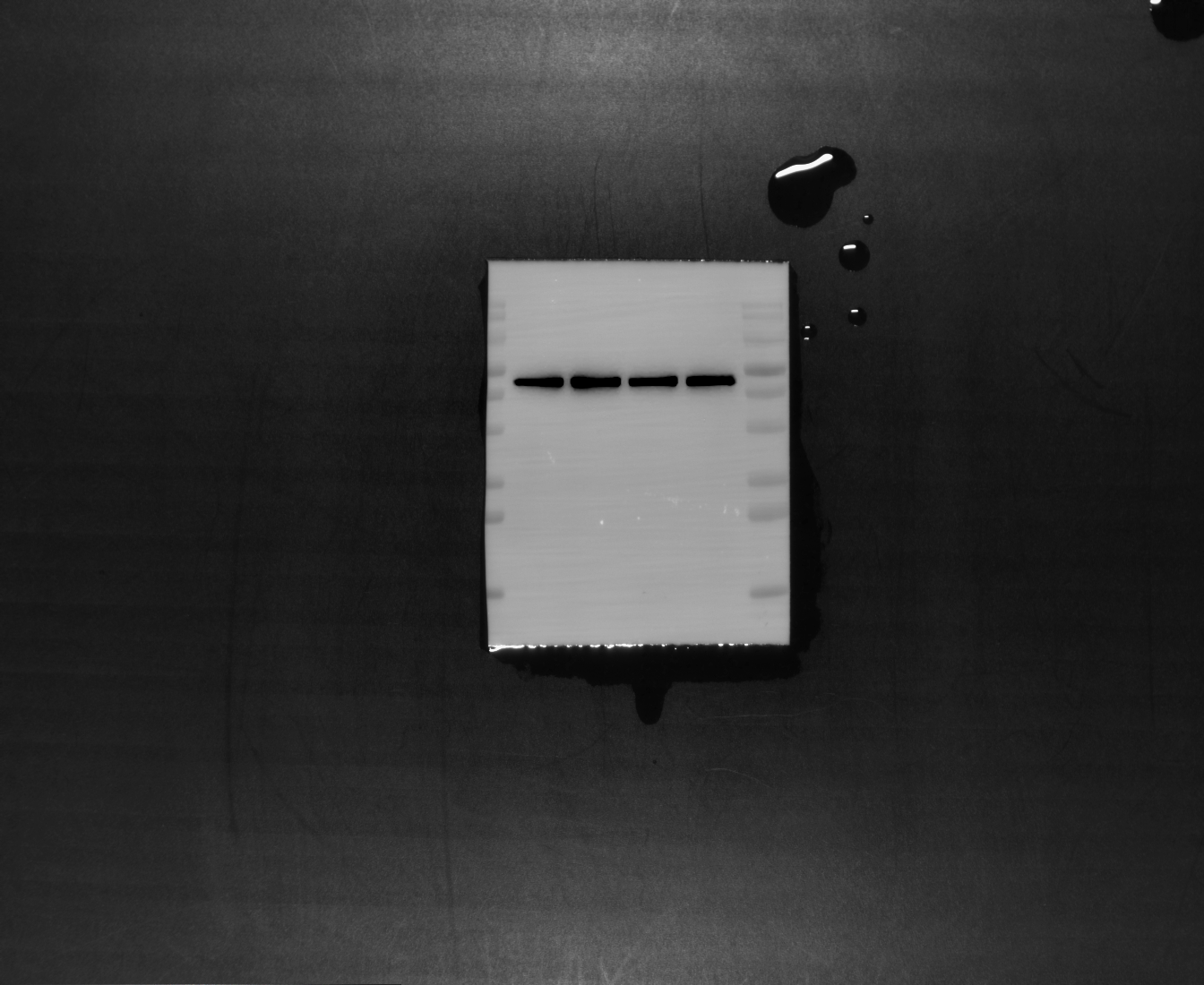

Supplement: File S1. Western Blot Data [file mmc3.zip › Western Blot Data/WB/Fig6C-PL45-LaminB1Menin.Tif]

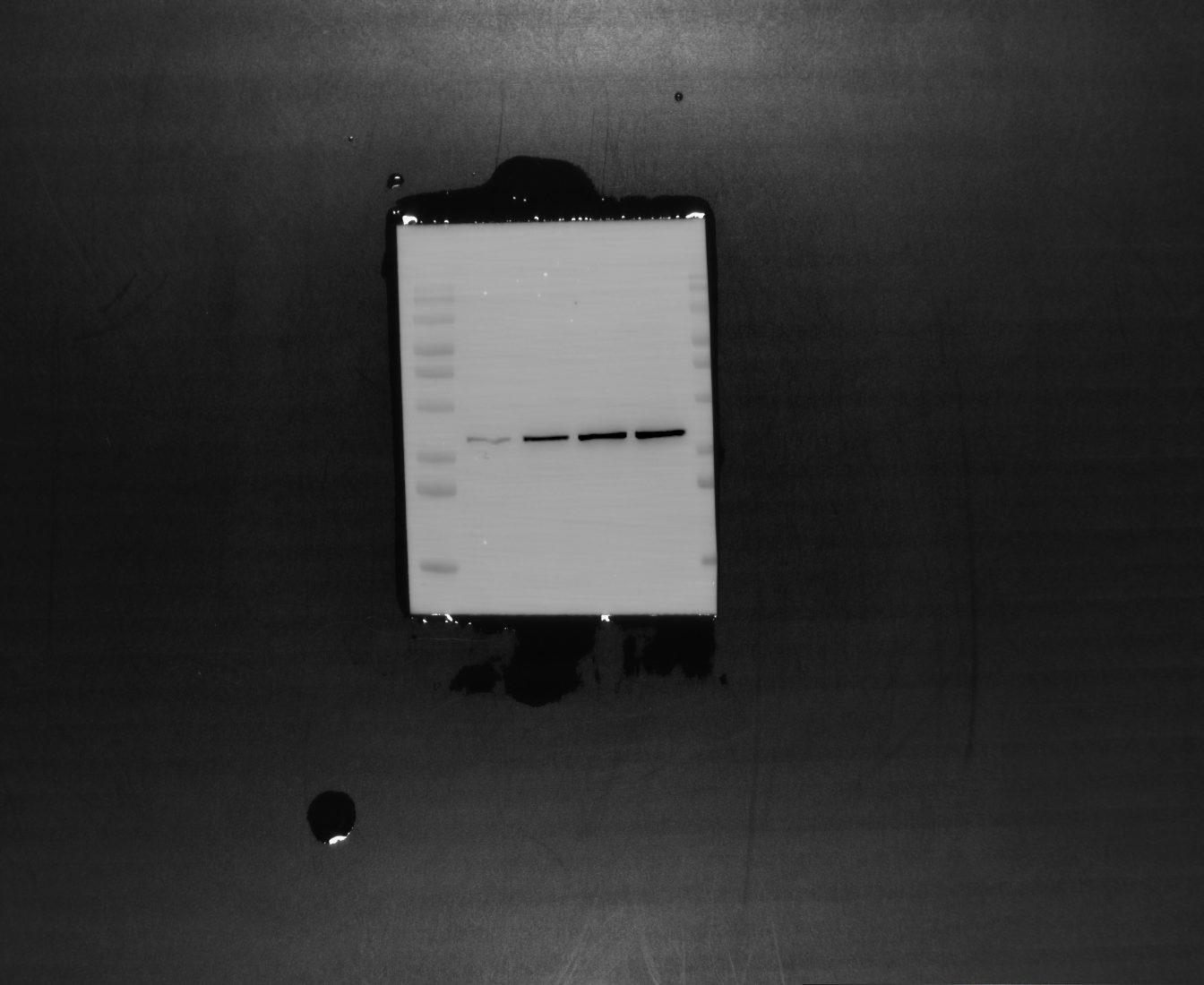

Supplement: File S1. Western Blot Data [file mmc3.zip › Western Blot Data/WB/Fig6C-PL45-NC-YBX1.Tif]

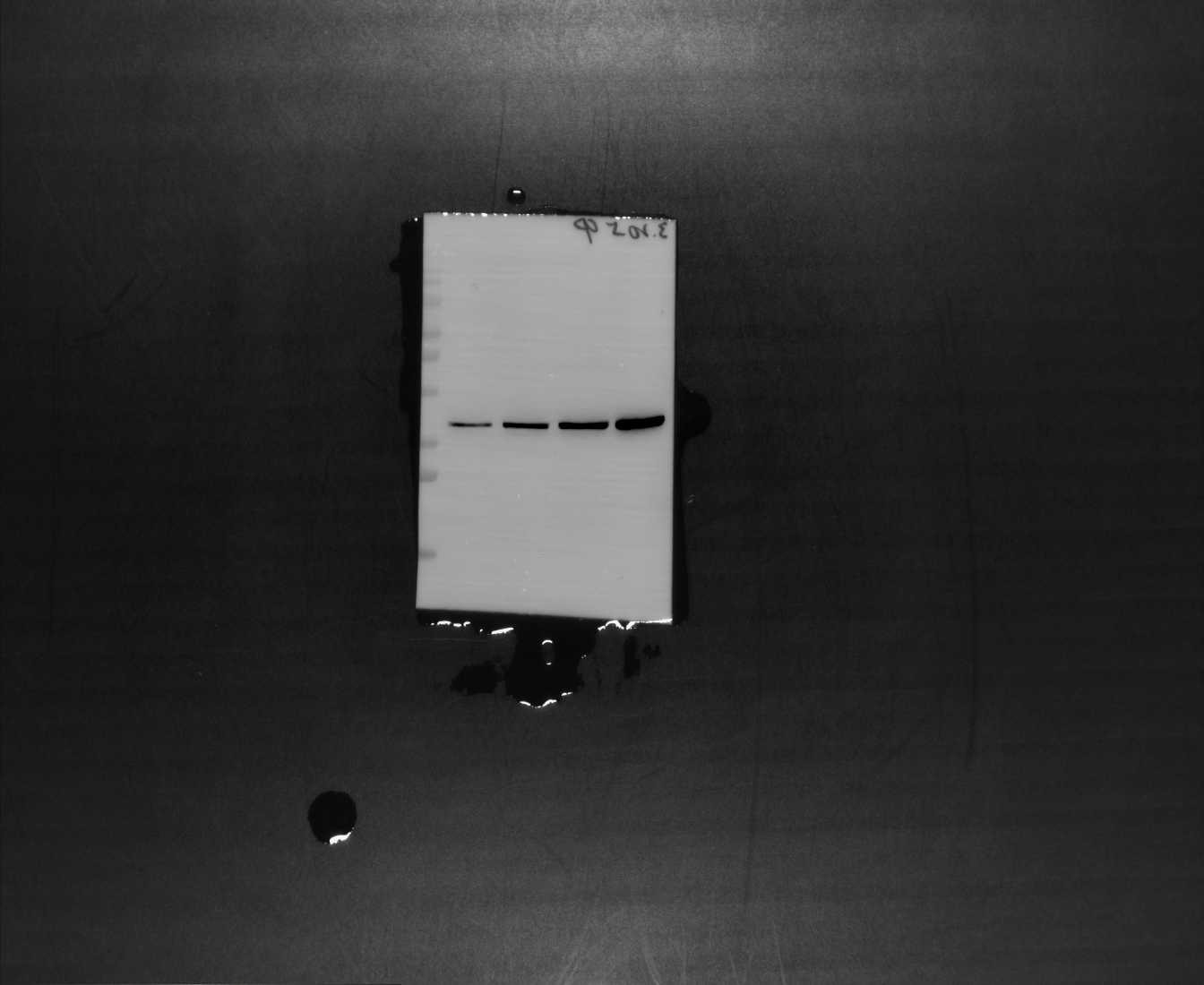

Supplement: File S1. Western Blot Data [file mmc3.zip › Western Blot Data/WB/Fig6C-PL45-OE-YBX1.Tif]

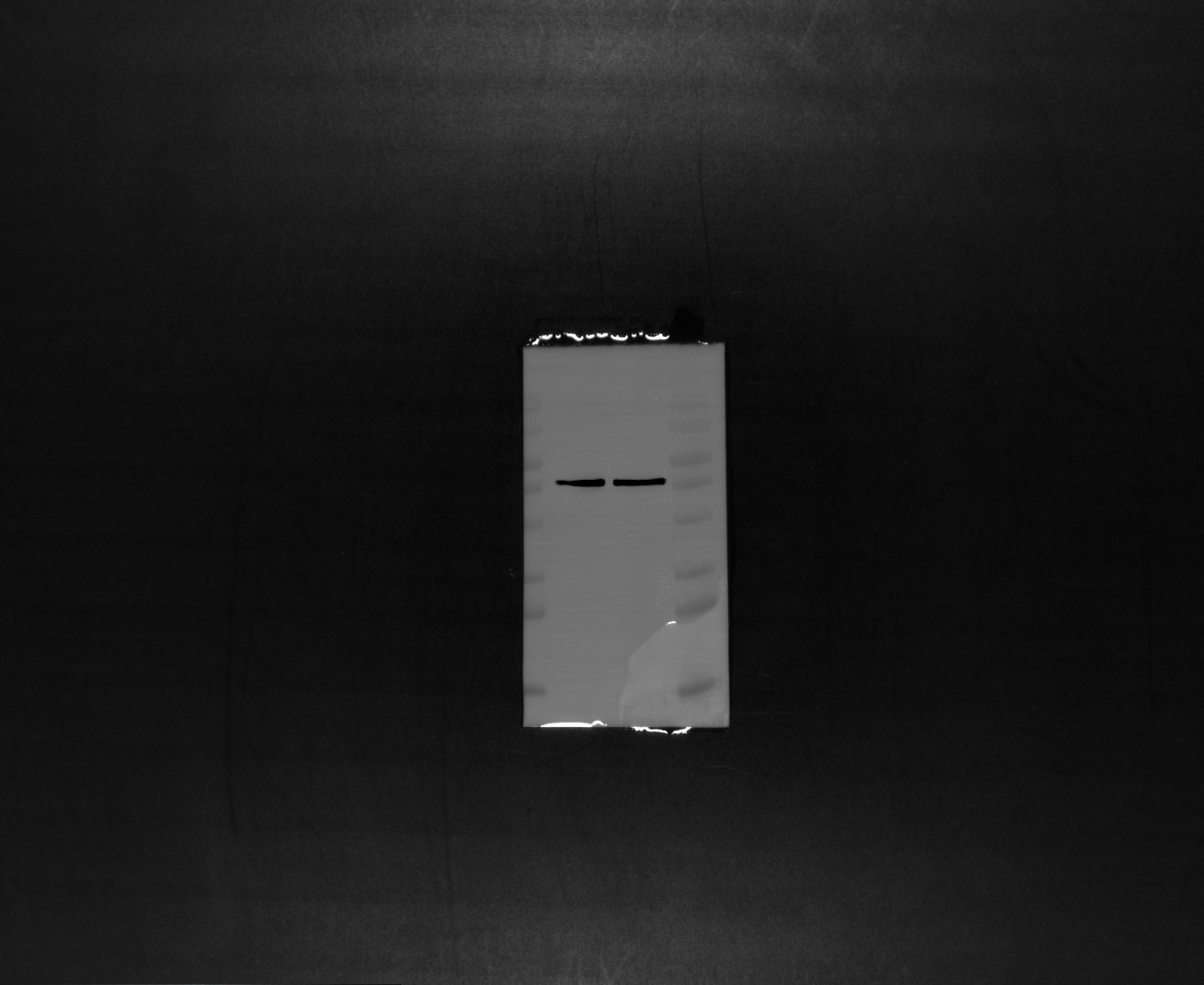

Supplement: File S1. Western Blot Data [file mmc3.zip › Western Blot Data/WB/Fig6D-BxPC-3-Input-LaminB1.Tif]

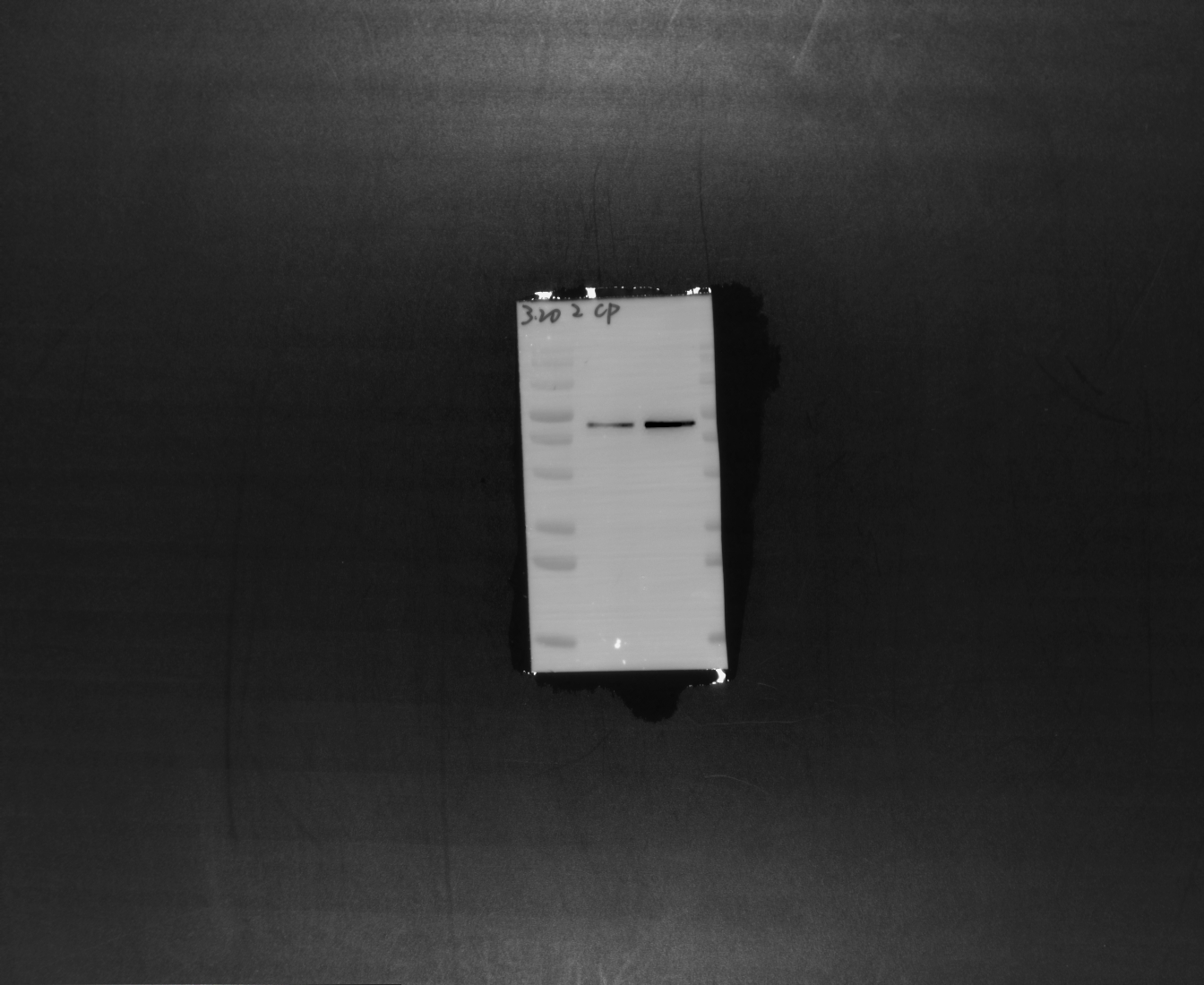

Supplement: File S1. Western Blot Data [file mmc3.zip › Western Blot Data/WB/Fig6D-BxPC-3-Ip-Menin.Tif]

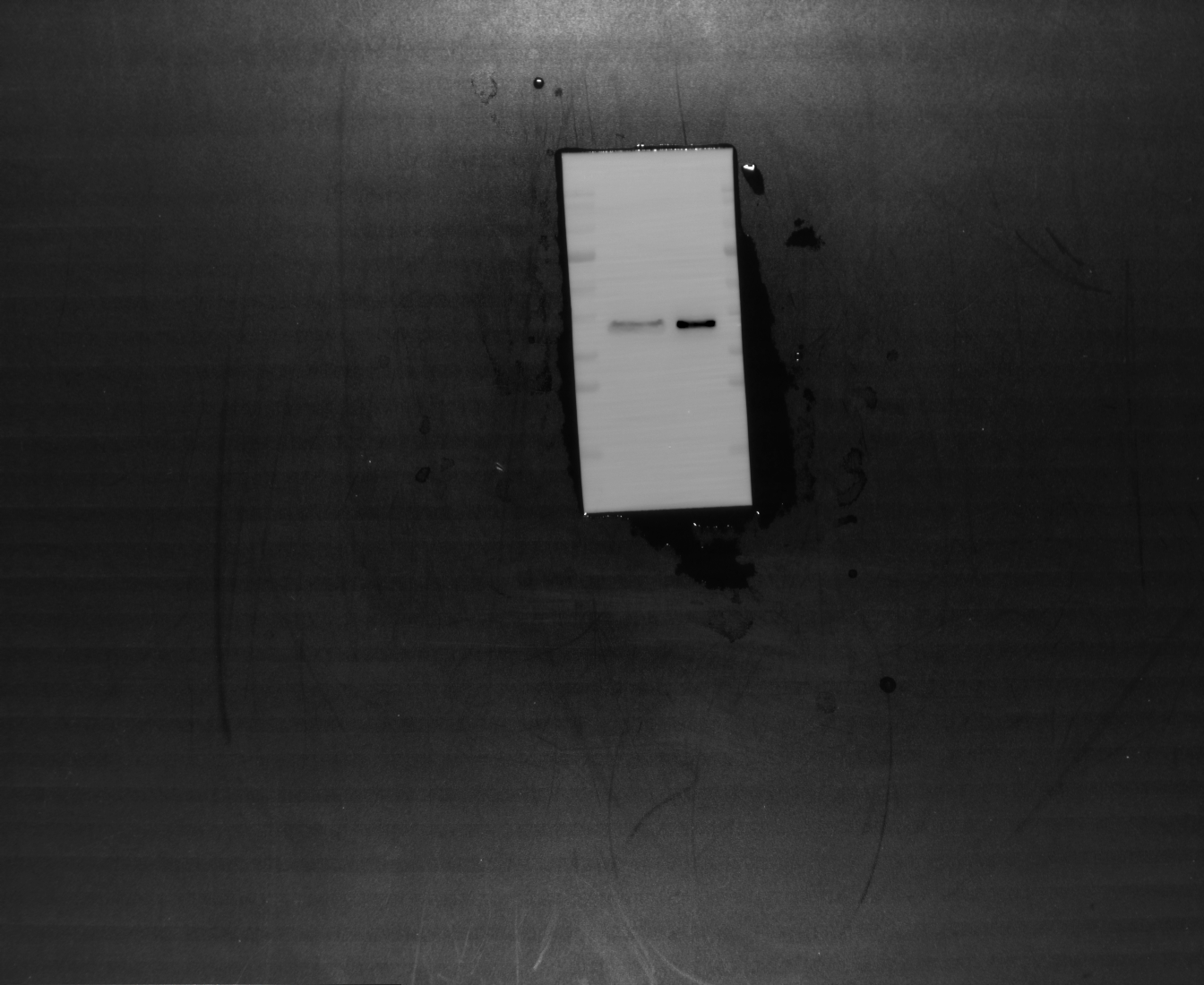

Supplement: File S1. Western Blot Data [file mmc3.zip › Western Blot Data/WB/Fig6D-BxPC-3-Ip-YBX1.Tif]

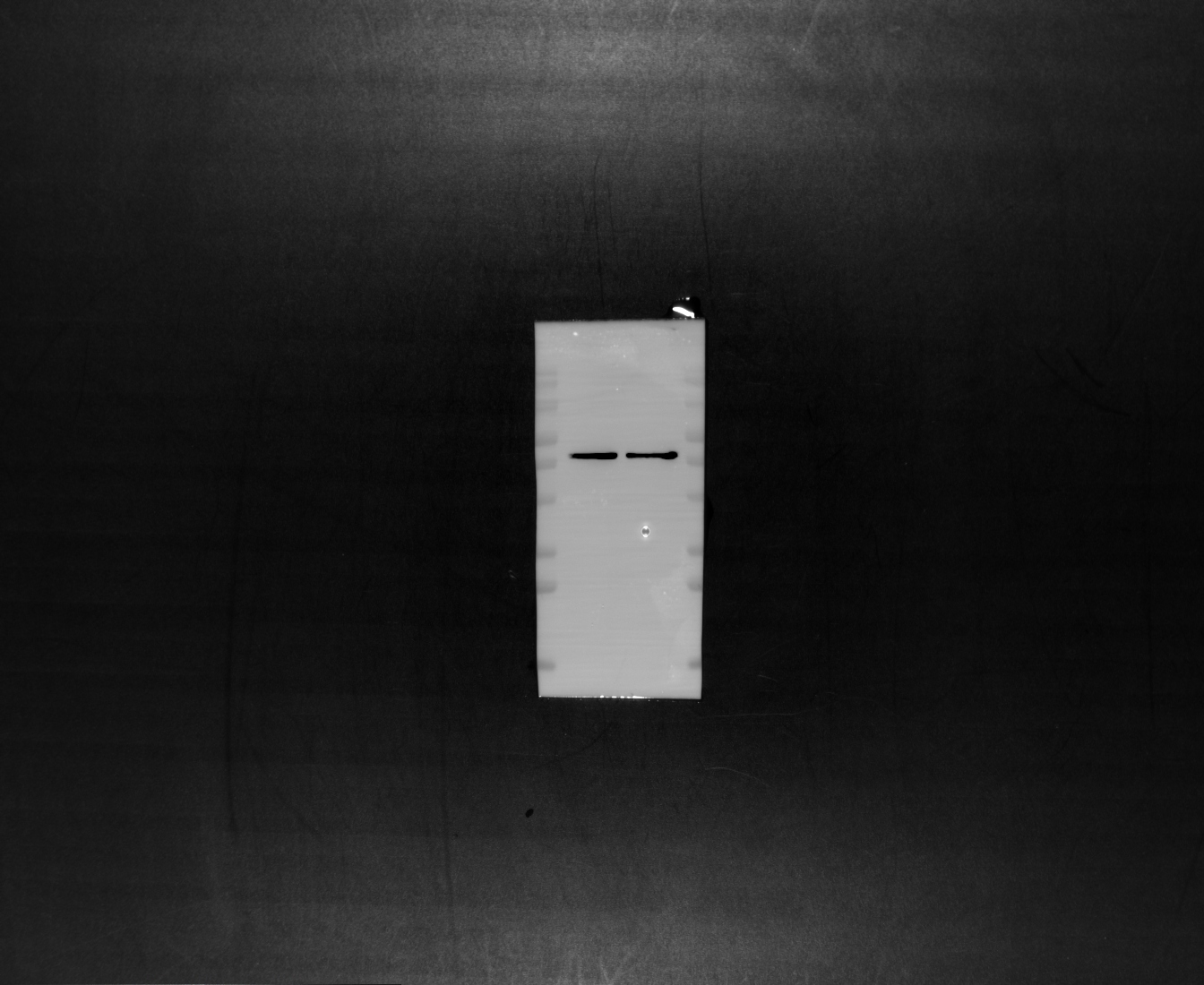

Supplement: File S1. Western Blot Data [file mmc3.zip › Western Blot Data/WB/Fig6D-PL45-Input-LaminB1.Tif]

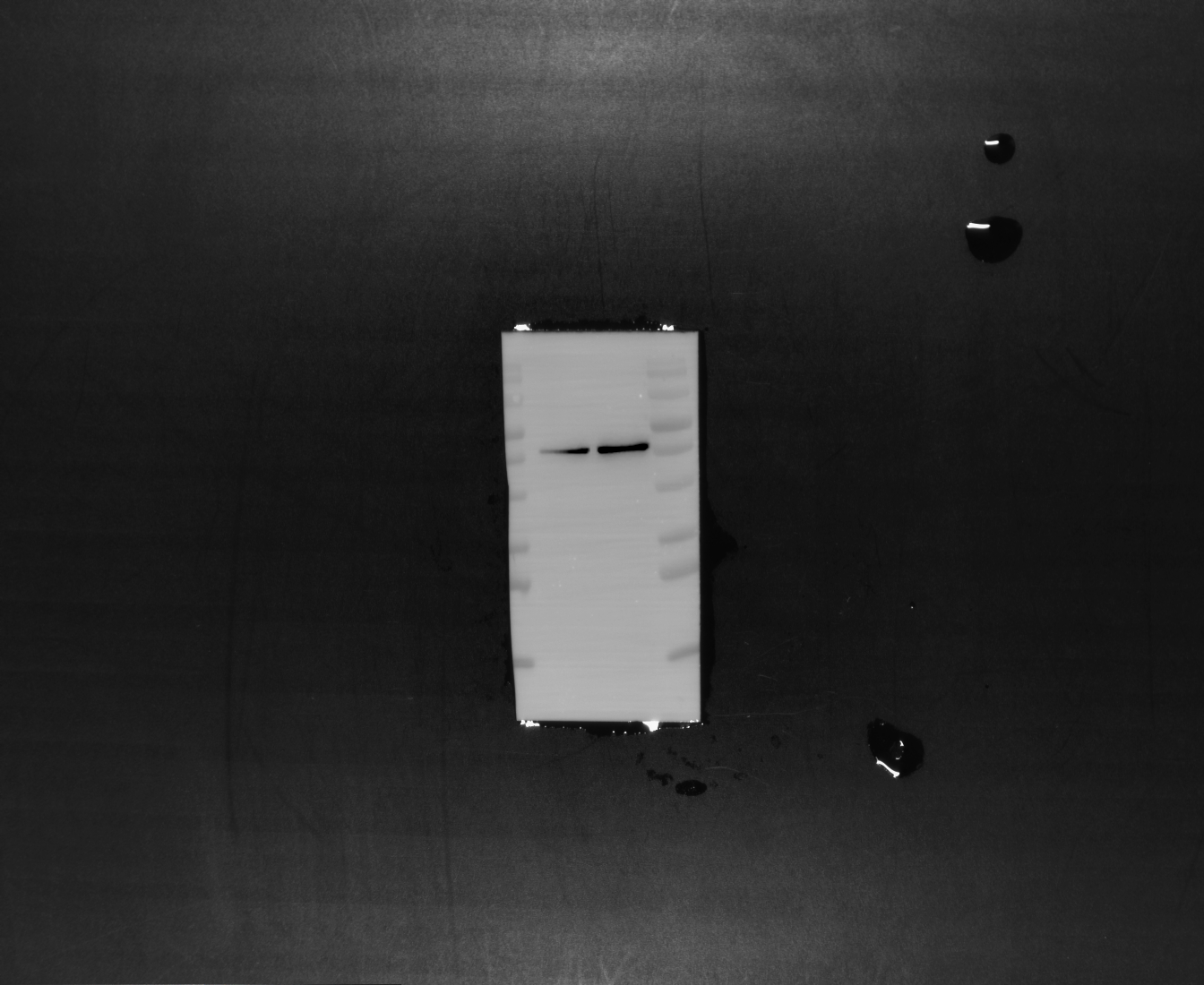

Supplement: File S1. Western Blot Data [file mmc3.zip › Western Blot Data/WB/Fig6D-PL45-Input-Menin.Tif]

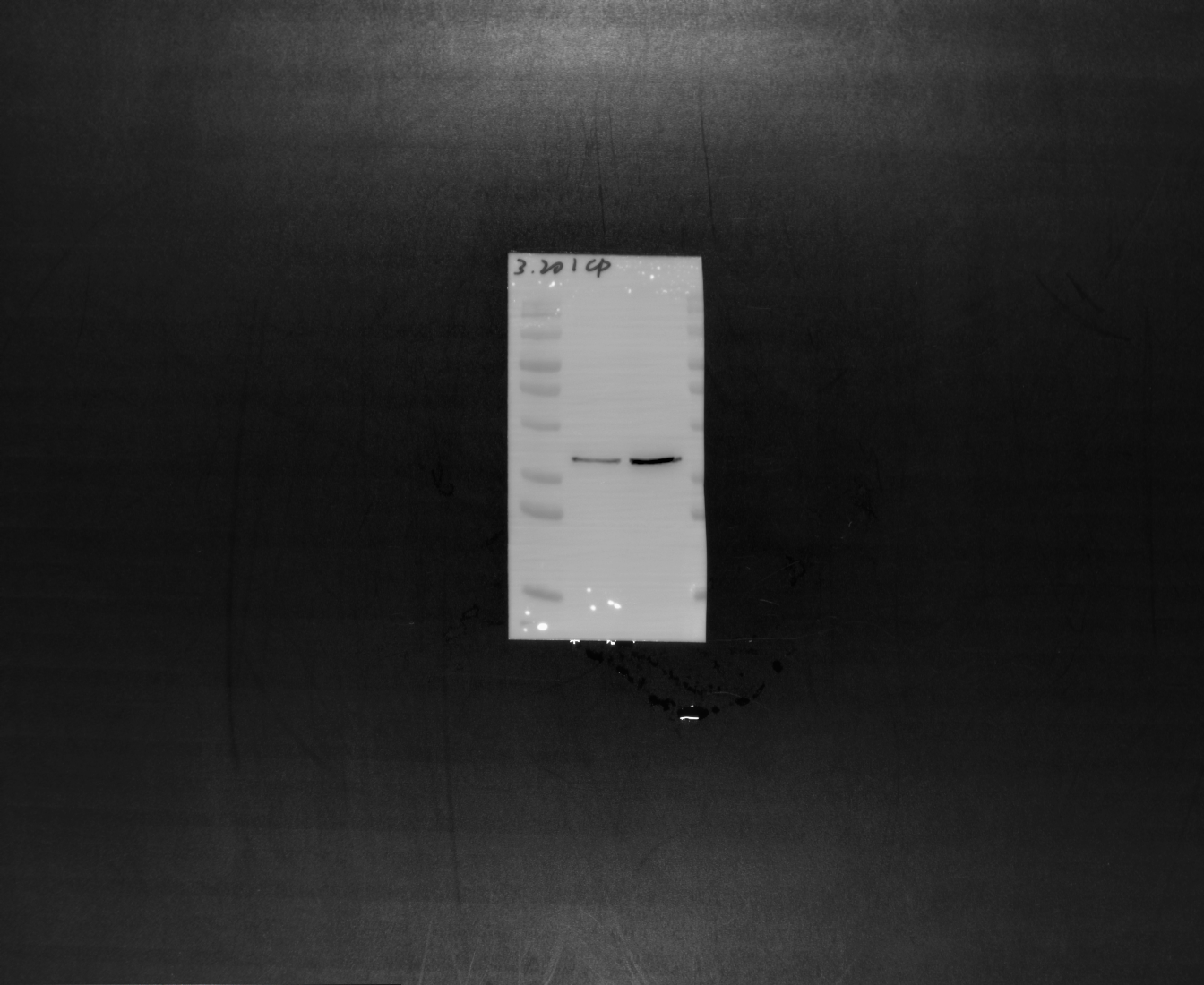

Supplement: File S1. Western Blot Data [file mmc3.zip › Western Blot Data/WB/Fig6D-PL45-IpYBX1.Tif]

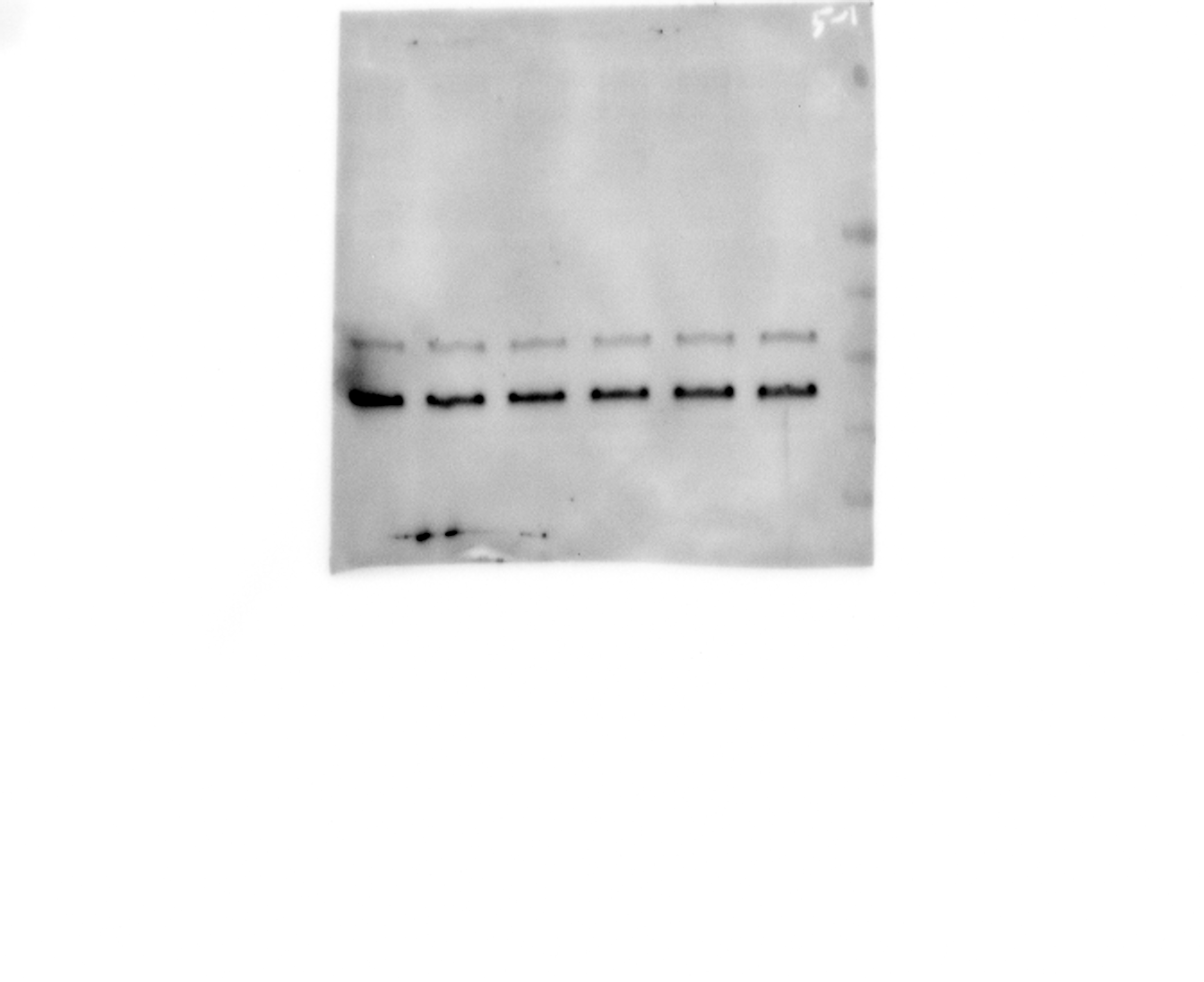

Supplement: File S1. Western Blot Data [file mmc3.zip › Western Blot Data/WB/Fig7E-GAPDH.Tif]

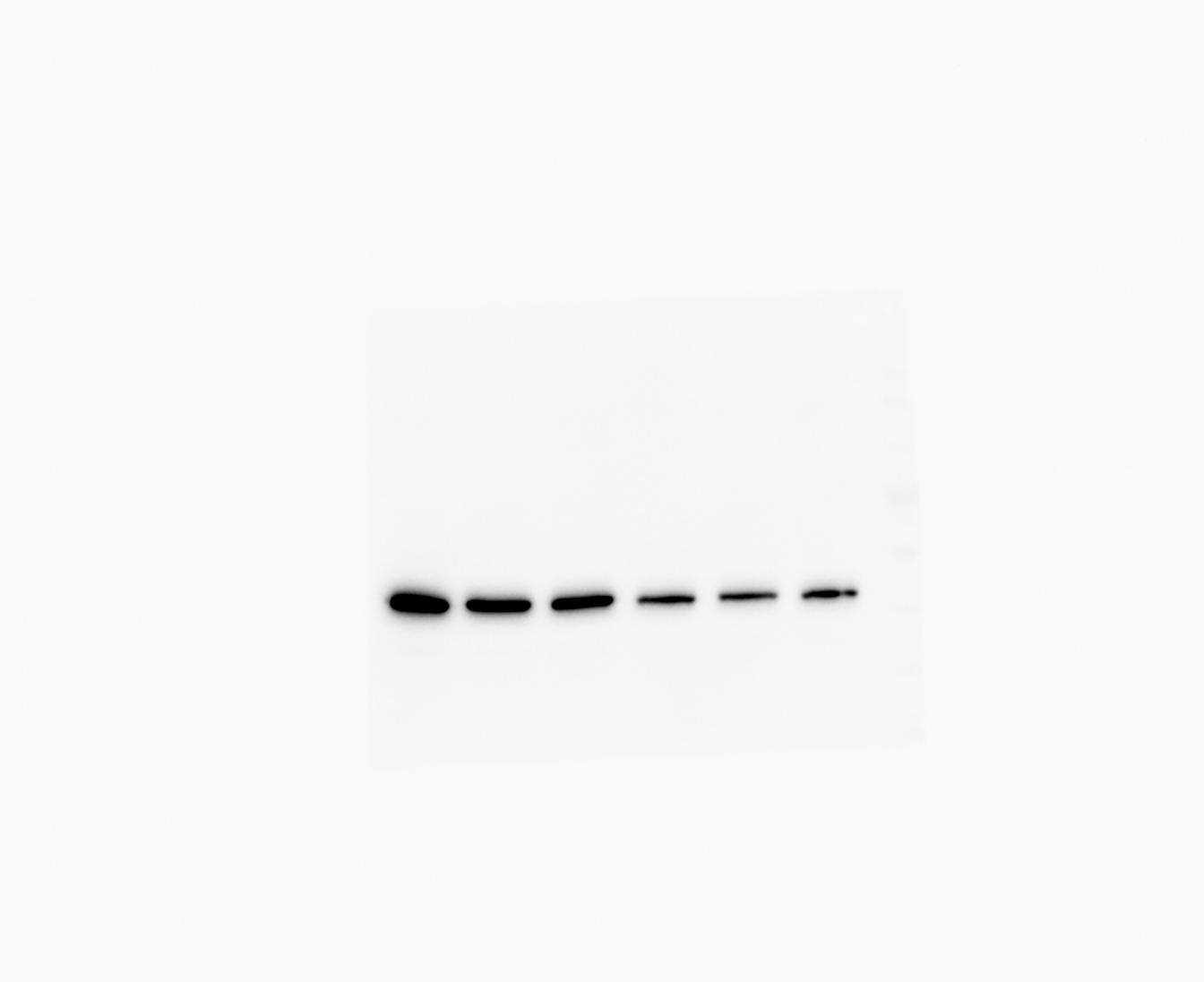

Supplement: File S1. Western Blot Data [file mmc3.zip › Western Blot Data/WB/Fig7E-GLUT1.Tif]

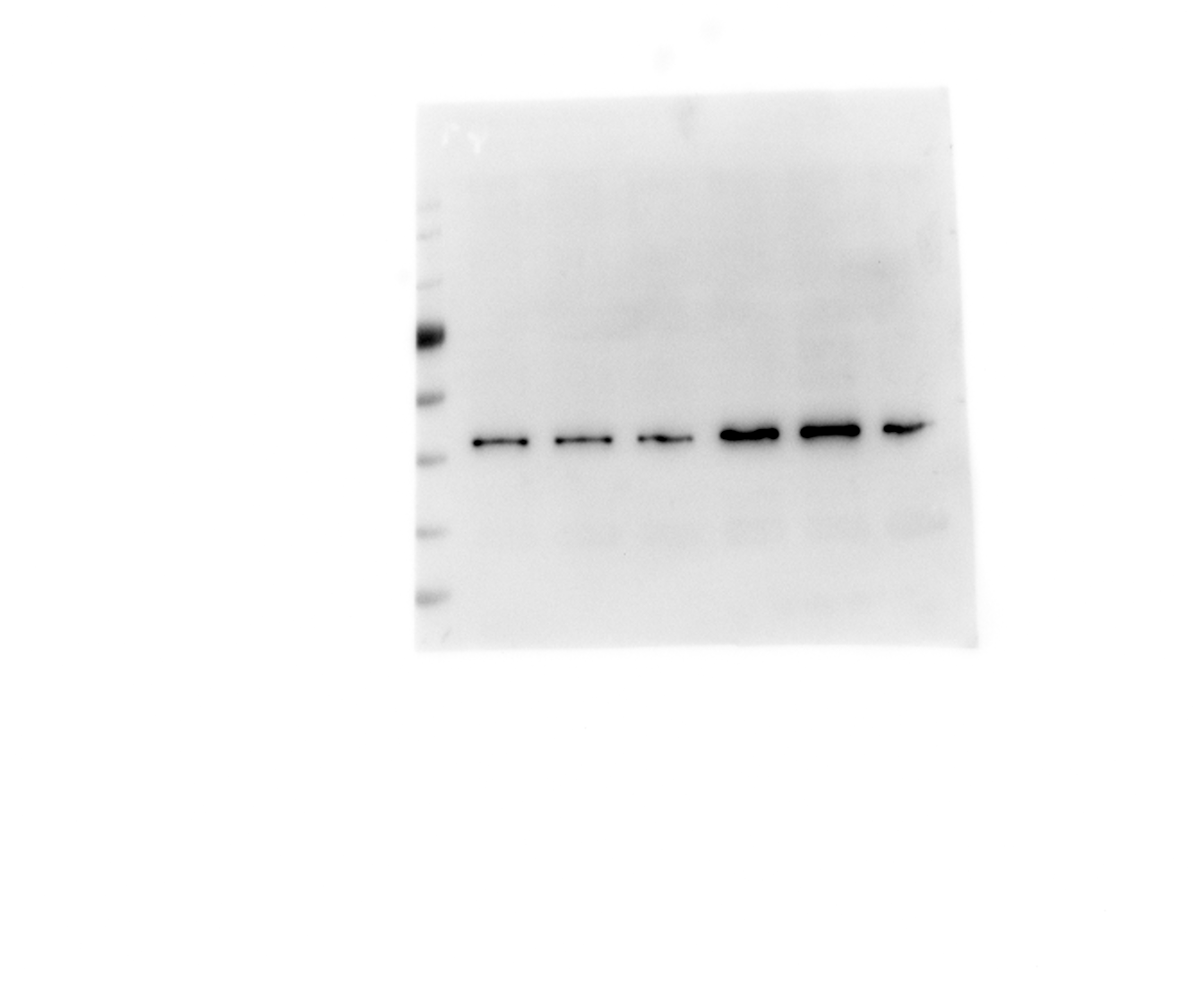

Supplement: File S1. Western Blot Data [file mmc3.zip › Western Blot Data/WB/Fig7E-HKDC1.png]

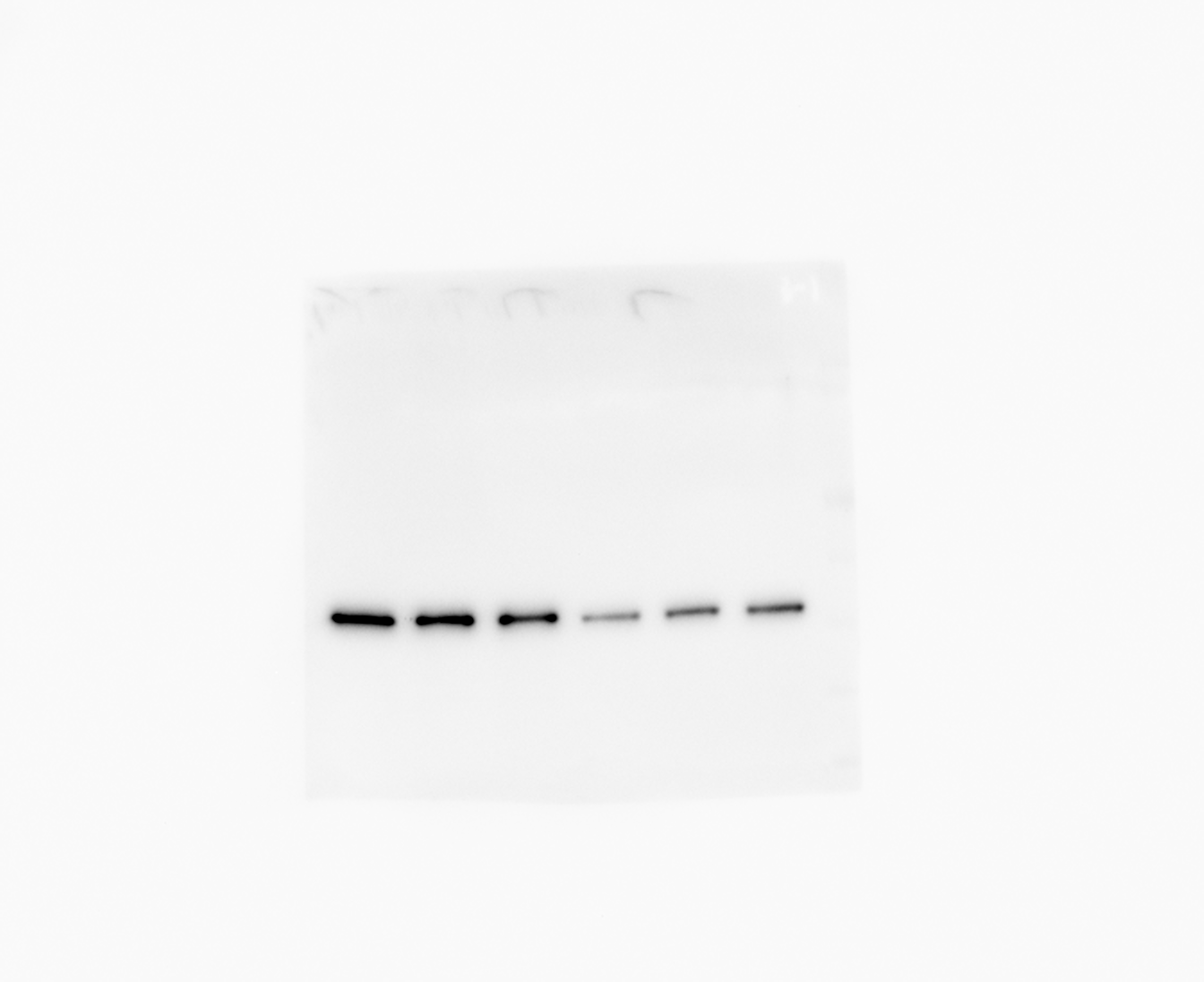

Supplement: File S1. Western Blot Data [file mmc3.zip › Western Blot Data/WB/Fig7E-LDHA.Tif]

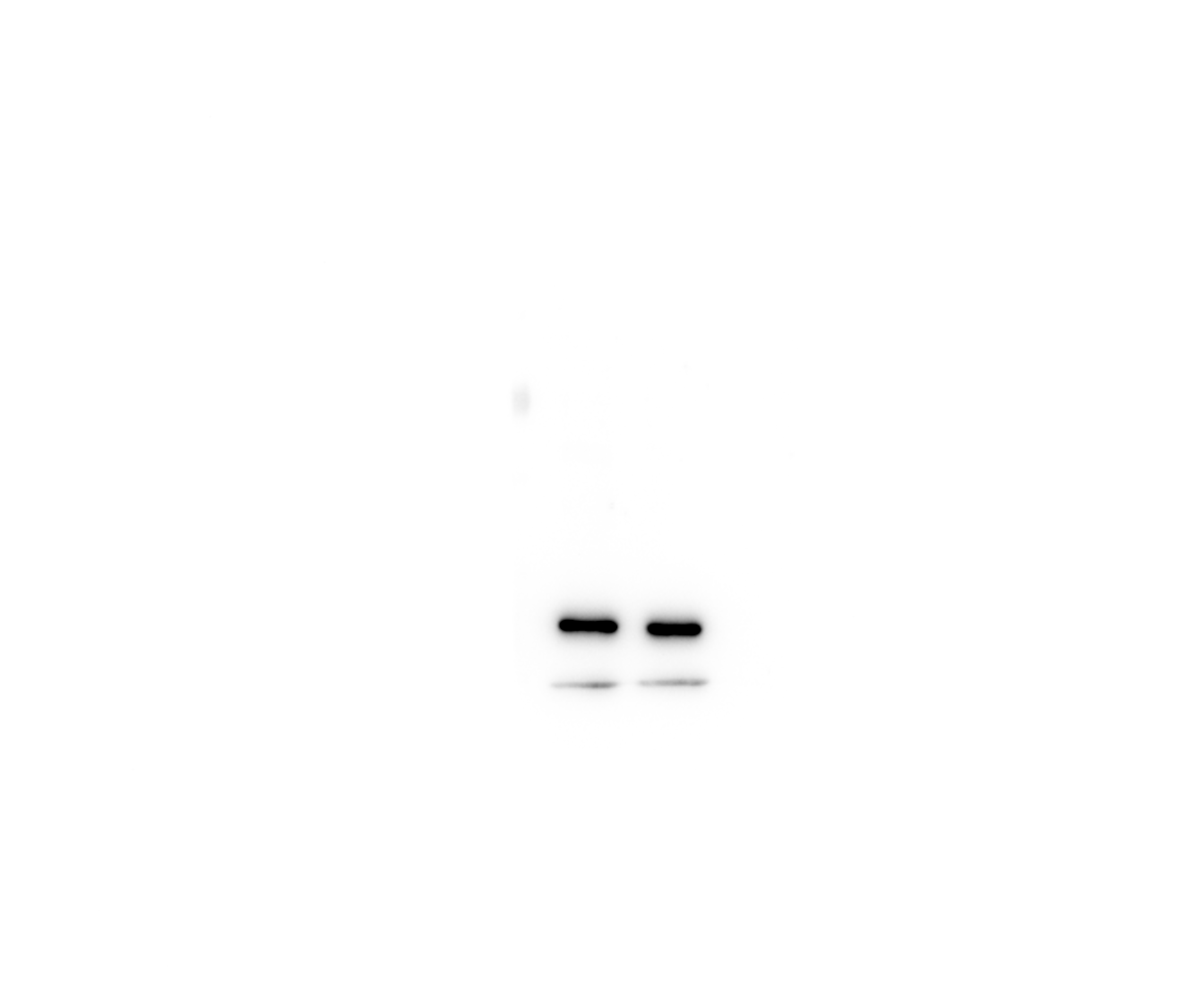

Supplement: File S1. Western Blot Data [file mmc3.zip › Western Blot Data/WB/FigS1-BxPC-3-GAPDH.Tif]

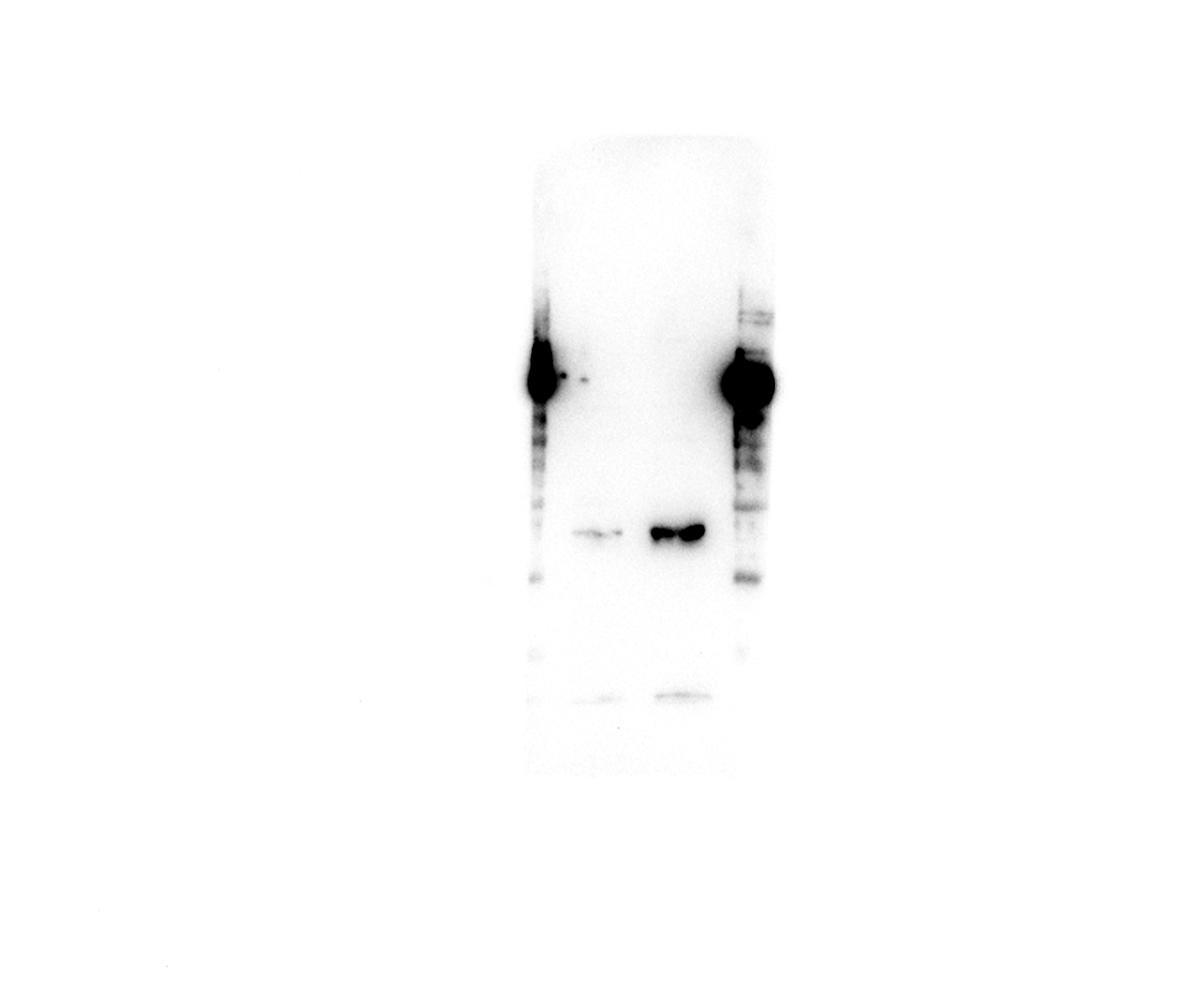

Supplement: File S1. Western Blot Data [file mmc3.zip › Western Blot Data/WB/FigS1-BxPC-3-MEN1.Tif]

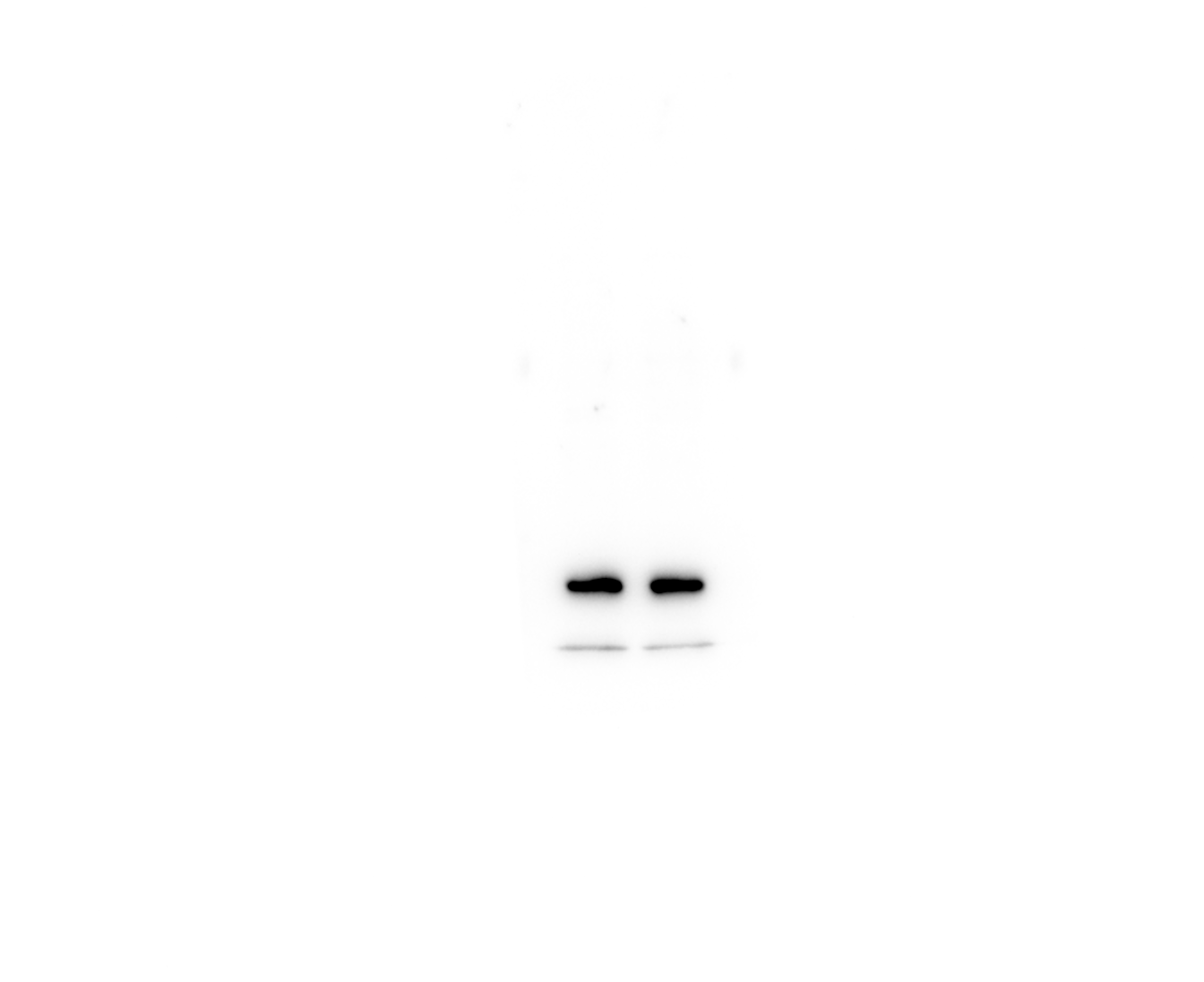

Supplement: File S1. Western Blot Data [file mmc3.zip › Western Blot Data/WB/FigS1-PL45-GAPDH.Tif]

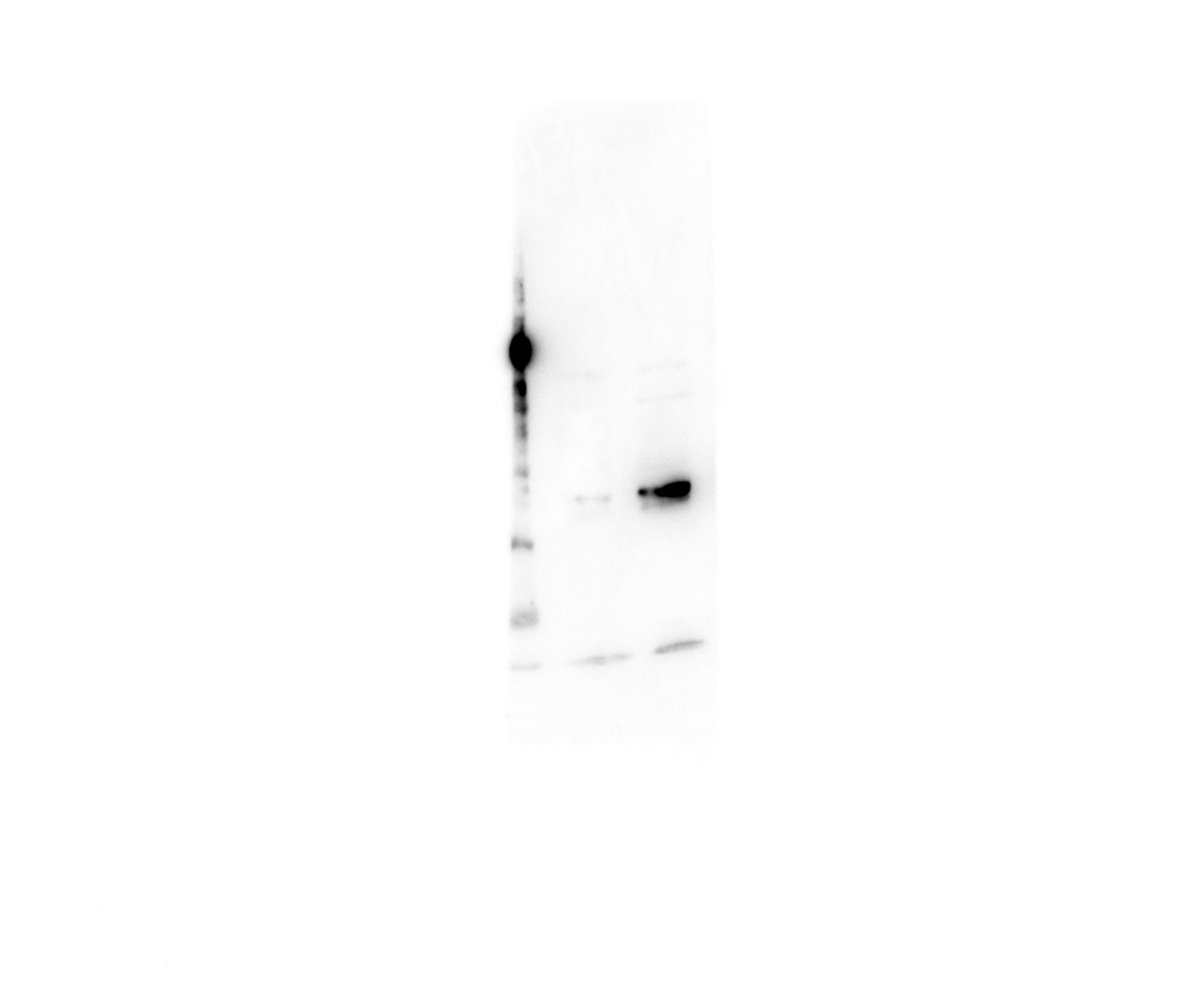

Supplement: File S1. Western Blot Data [file mmc3.zip › Western Blot Data/WB/FigS1-PL45-MEN1.Tif]

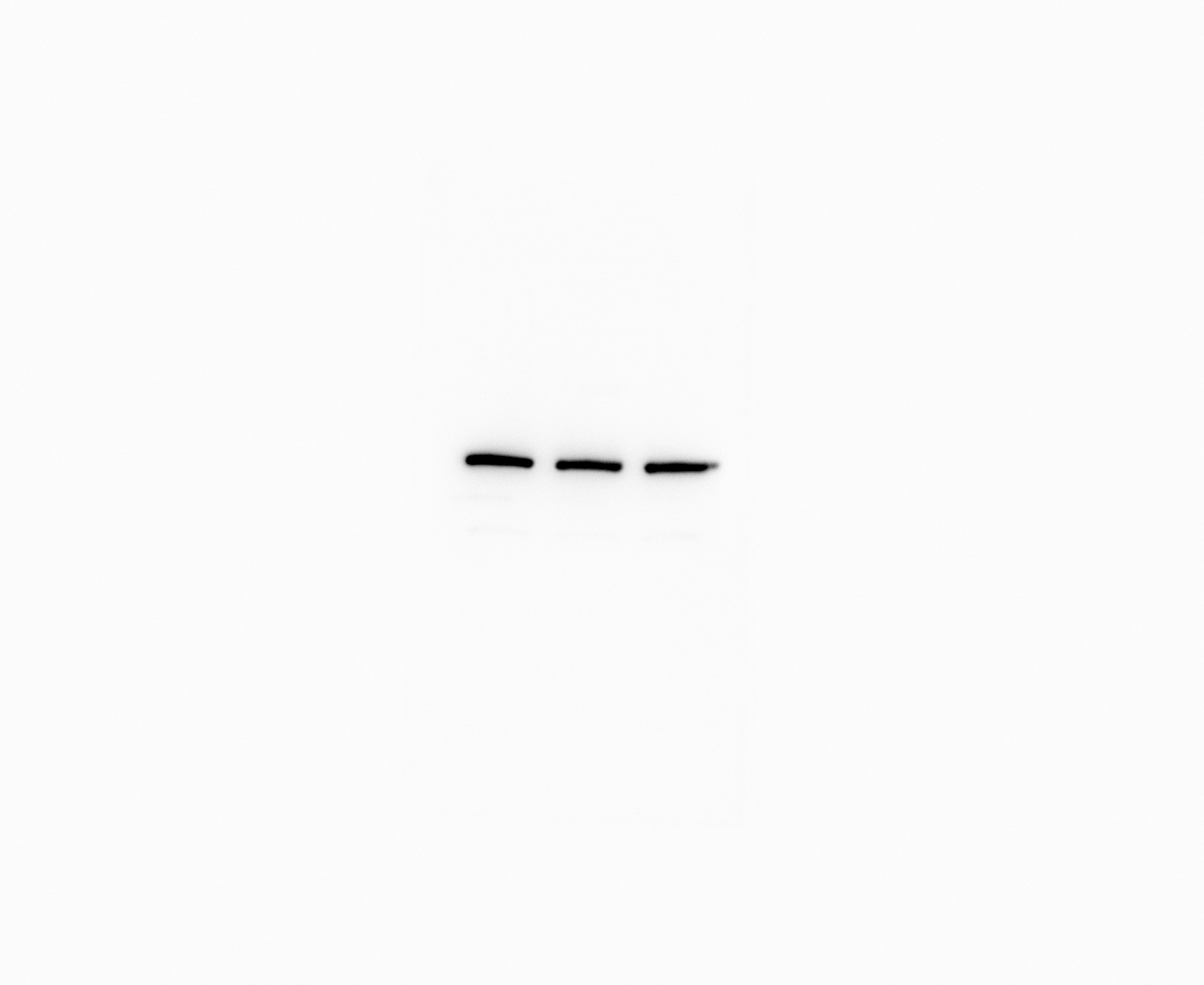

Supplement: File S1. Western Blot Data [file mmc3.zip › Western Blot Data/WB/FigS2-BxPC-3-GAPDH.tif]

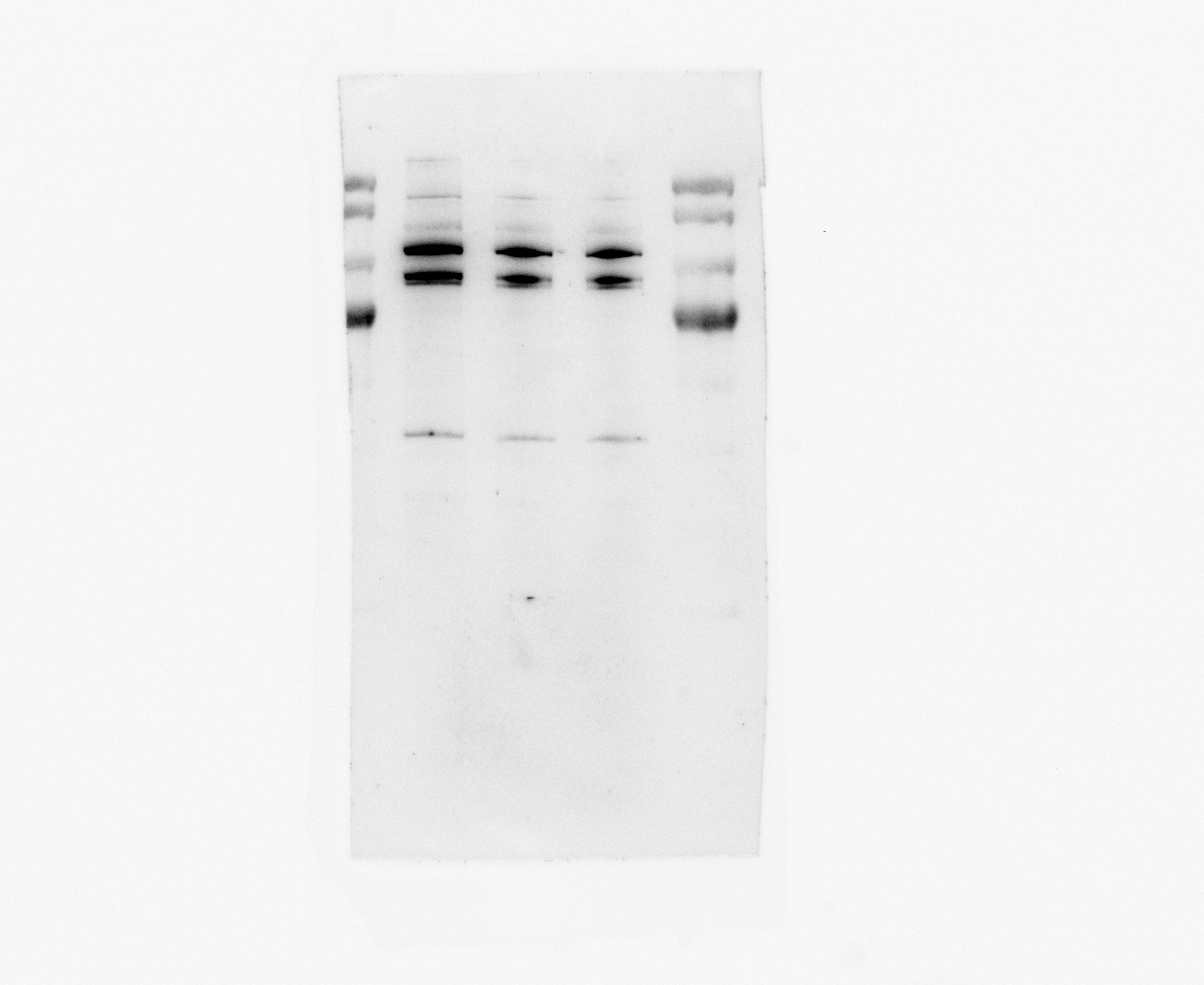

Supplement: File S1. Western Blot Data [file mmc3.zip › Western Blot Data/WB/FigS2-BxPC-3-HKDC1.tif]

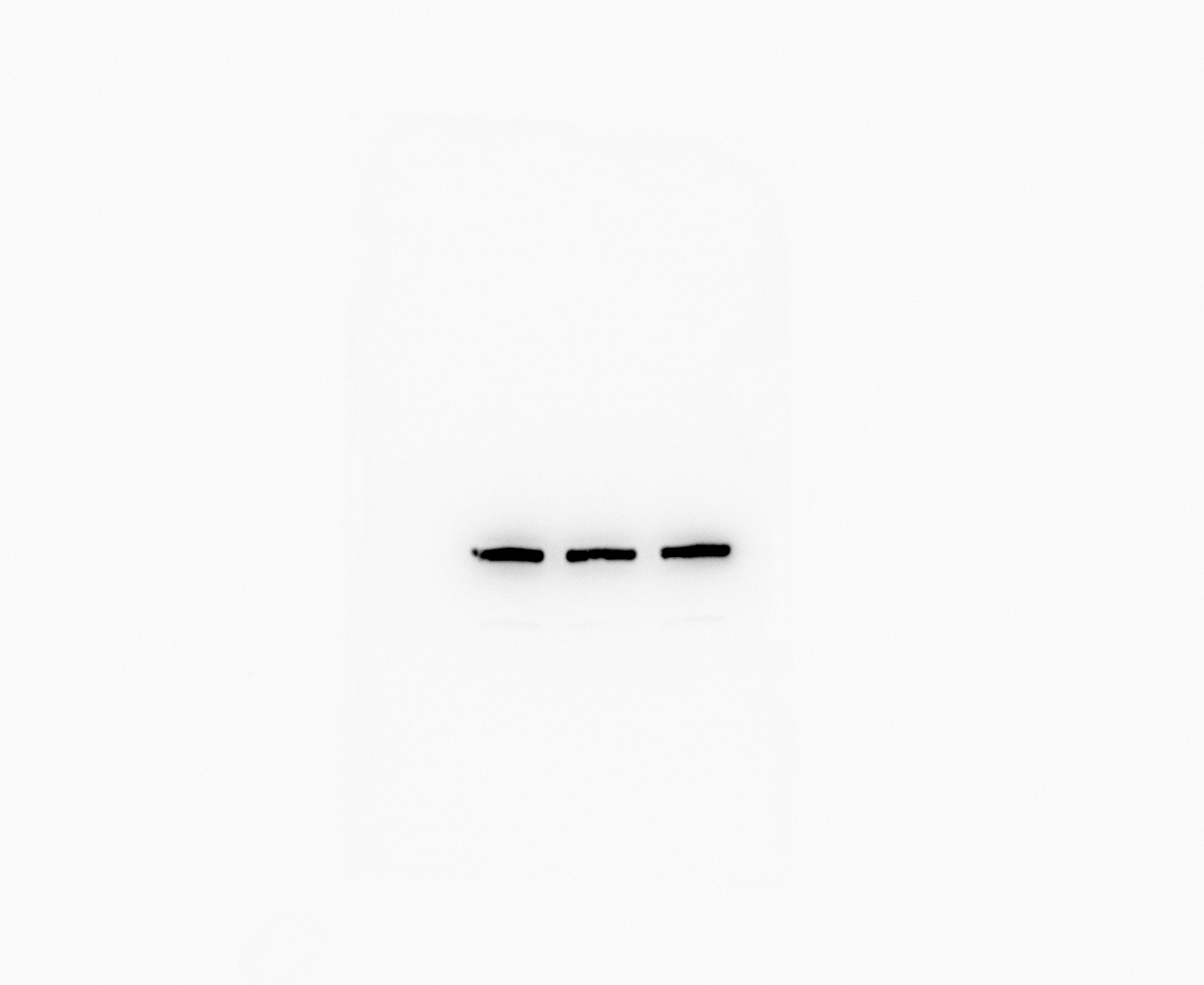

Supplement: File S1. Western Blot Data [file mmc3.zip › Western Blot Data/WB/FigS2-PL45-GAPDH.tif]

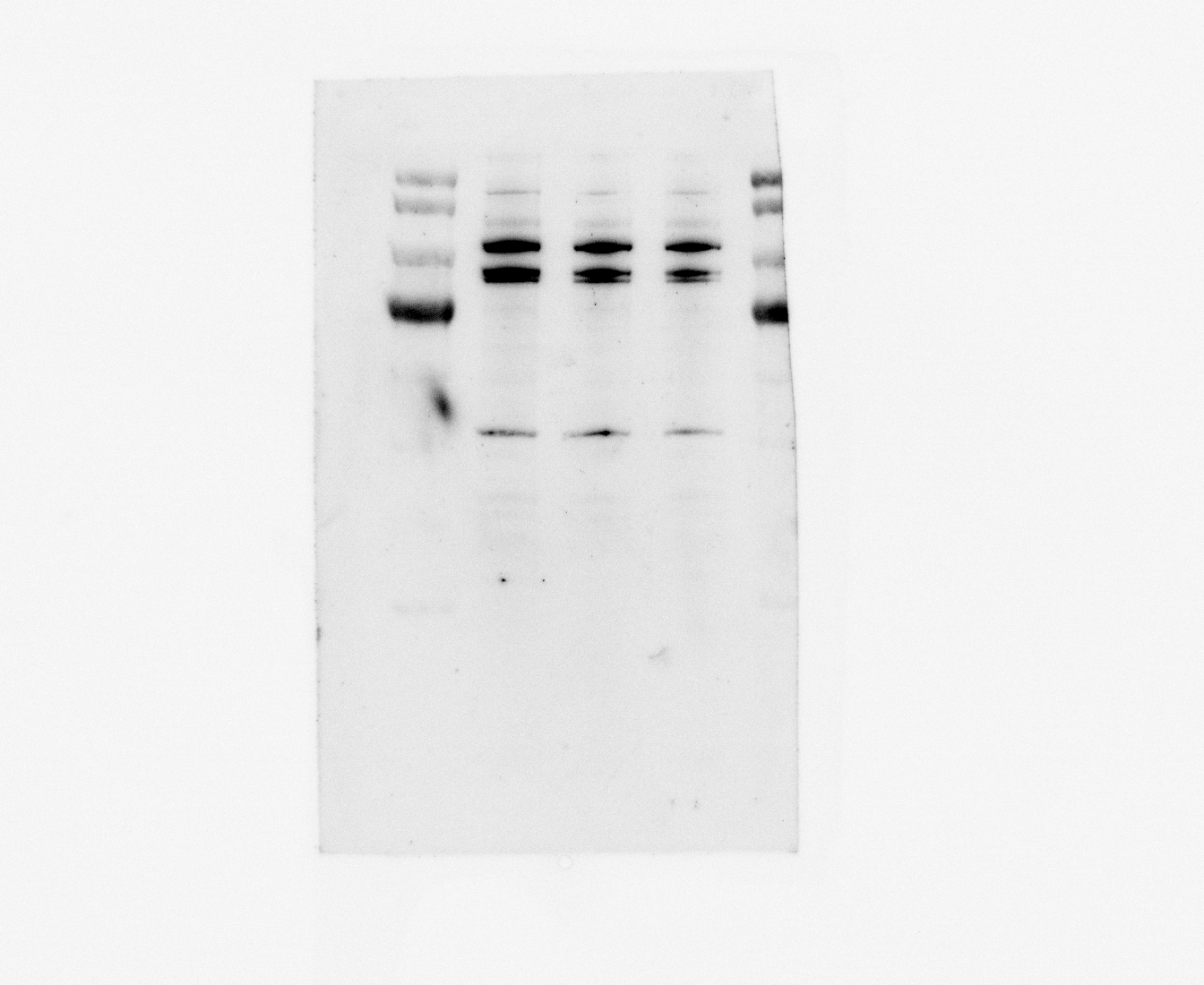

Supplement: File S1. Western Blot Data [file mmc3.zip › Western Blot Data/WB/FigS2-PL45-HKDC1.tif]

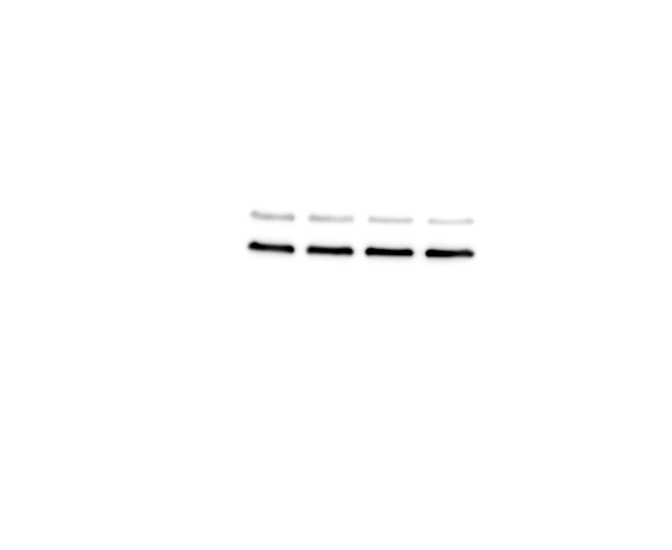

Supplement: File S1. Western Blot Data [file mmc3.zip › Western Blot Data/WB/FigS3-GAPDH.tif]

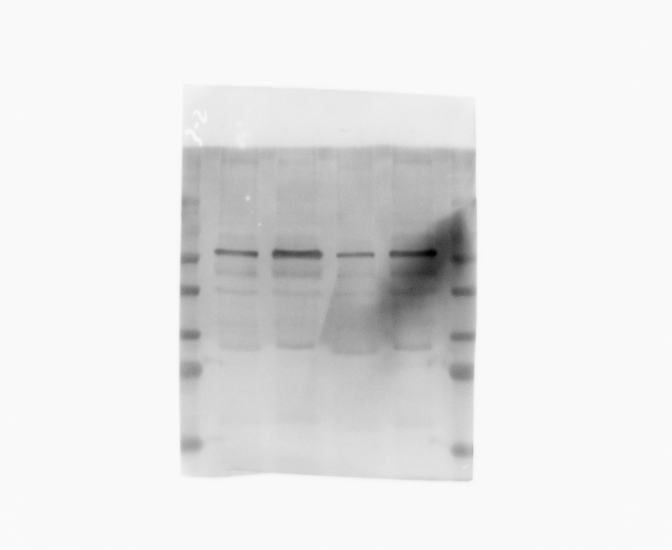

Supplement: File S1. Western Blot Data [file mmc3.zip › Western Blot Data/WB/FigS3-HKDC1.tif]
